# Novel Chemical Scaffolds to Inhibit the Neutral Amino Acid Transporter B^0^AT1 (SLC6A19), a Potential Target to Treat Metabolic Diseases

**DOI:** 10.3389/fphar.2020.00140

**Published:** 2020-02-28

**Authors:** Aditya Yadav, Nishank Shah, Praveen Kumar Tiwari, Kiran Javed, Qi Cheng, Indrapal Singh Aidhen, Stefan Bröer

**Affiliations:** ^1^ Research School of Biology, Australian National University, Canberra, ACT, Australia; ^2^ Department of Chemistry, Indian Institute of Technology Madras, Chennai, India

**Keywords:** phenylketonuria, steatohepatitis, non-alcoholic steatohepatitis, solute carrier, high throughput screening, HTS

## Abstract

Lack of B^0^AT1 (SLC6A19) partially protects mice against the onset of non-alcoholic steatohepatitis (NASH). To achieve a similar outcome through pharmacological treatment, we improved previously identified inhibitors of B^0^AT1 by medicinal chemistry and identified second generation inhibitors by high through-put screening. Modified diarylmethine compounds inhibited B^0^AT1 with IC_50_ values ranging from 8–90 μM. A second generation of inhibitors was derived from high-throughput screening and showed higher affinity (IC_50_ of 1–15 μM) and strong selectivity against amino acid transporters with similar substrate specificity, such as ASCT2 (SLC1A5) and LAT1 (SLC7A5). All compounds were unrelated to B^0^AT1 substrates, but were likely to bind in the vicinity of the substrate binding site.

## Introduction

Transport of amino acids by epithelial cells of the intestine and kidney plays an important role in organismic amino acid homeostasis (Cynober, 2002; Broer and Broer, 2017). Moreover, amino acids are important signalling molecules that regulate protein biosynthesis, autophagy, ribosome biogenesis, gluconeogenesis, neurotransmitter levels, release of hormones, and many other functions. As a result, amino acid transporters are increasingly investigated as potential drug targets (Broer, 2018). The epithelial neutral amino acid transporter B^0^AT1 (SLC6A19) was initially identified as the molecule mutated in the rare condition Hartnup disorder (Kleta et al., 2004; Seow et al., 2004). While the disorder was originally described with more severe clinical symptoms (Baron et al., 1956), it is now evident that the vast majority of individuals with Hartnup disorder remain clinically asymptomatic, which has been attributed to improved diets (Wilcken et al., 1977; Broer, 2009). B^0^AT1 is expressed in the apical membrane of intestinal and renal epithelial cells (Kleta et al., 2004; Broer et al., 2011). Thus, mutations of B^0^AT1 cause intestinal malabsorption and renal aminoaciduria. Renal aminoaciduria is restricted to neutral amino acids and defines the disorder in the absence of other clinical symptoms. Typically, plasma amino levels are normal or at the lower end of the normal range in individuals with Hartnup disorder (Levy, 2001) and this is also observed in fasting B^0^AT1 knock-out mice (Javed et al., 2018). However, upon nutrient intake, amino acid absorption is delayed and differences in plasma neutral amino acid levels are observed (Singer et al., 2012; Jiang et al., 2015; Javed and Broer, 2019). In B^0^AT1 knock-out mice this generates a metabolic phenotype that is dominated by two effects. First, delayed absorption causes an increased amino acid load in distal sections of the small intestine, where the majority of enteroendocrine cells are located (Jiang et al., 2015). This enhances the release of incretins glucagon-like peptide 1 (GLP-1) and gastric inhibitory peptide (GIP). Secondly, delayed arrival of amino acids in the portal vein cause an amino acid restriction phenotype in the liver, which is probably intensified by the loss of amino acids in the urine (Jiang et al., 2015). Protein restriction is now considered an important metabolic signal that at least in part is mediated by the release from liver of the endocrine hormone FGF21 (De Sousa-Coelho et al., 2012; Maida et al., 2016; Maida et al., 2018). FGF21 enhances lipid metabolism in liver and other tissues (Badman et al., 2007). As a result FGF21 is now investigated as a hormone to treat fatty liver associated diseases (Fisher et al., 2014). In agreement with protein restriction occurring in the liver of B^0^AT1 knock-out mice, high plasma levels of FGF21 and reduced triglyceride levels in liver and blood plasma are observed (Jiang et al., 2015). Thus, inhibition of B^0^AT1 could be used to induce endogenous FGF21, thereby reducing lipotoxicity and dyslipidemia. B^0^AT1 knock-out mice show additional features, such as browning of adipose tissue and increased glucose consumption by the heart (Jiang et al., 2015).

More recently, B^0^AT1 has also been suggested as a target to normalize amino acid imbalance in specific inherited disorders such as phenylketonuria (PKU) and urea cycle disorders (Belanger et al., 2018). In phenylketonuria, plasma levels of phenylalanine are highly elevated due to mutation of the enzyme phenylalanine hydroxylase, which initiates the degradation of the amino acid by converting it to tyrosine. This causes disturbance of amino acid homeostasis and neurotransmitter synthesis in the brain. Untreated phenylketonuria causes intellectual disability, as a result, individuals with the disease have to adhere strictly to a diet devoid of phenylalanine. B^0^AT1 is the only transporter for phenylalanine in the apical membrane of small intestine enterocytes and kidney tubules (Broer, 2008). Similar to humans, plasma phenylalanine levels in the *Pah^enu2^* mouse model of PKU are highly elevated. This translates into elevated levels of phenylalanine in the brain and reduced levels of neurotransmitters serotonin, dopamine, and norepinephrine (Belanger et al., 2018). Elevated levels of reactive astrocytes were found, replicating human PKU. Introducing a B^0^AT1 knock-out in Pah^enu2^ mice normalized phenylalanine levels in brain and plasma and reduced the neuropathological phenotype. Thus, efficient inhibitors of B^0^AT1 could be used to treat metabolic diseases such as NASH, NAFLD, phenylketonuria, urea cycle deficiency, and related disorders.

The pharmacology of B^0^AT1 has improved due to a better understanding of the biochemistry of the protein. Nimesulide (IC_50_ 23 μM) and related compounds were identified as inhibitors of B^0^AT1 using native transporter purified from rat brush-border membranes and reconstituted into proteoliposomes (Pochini et al., 2014). In order to develop inhibitors of the human B^0^AT1, recombinant expression was optimized in CHO cells (Cheng et al., 2017). Recombinant expression of B^0^AT1 requires co-expression with its accessory proteins collectrin (Danilczyk et al., 2006; Malakauskas et al., 2007) or angiotensin-converting enzyme 2 (ACE2) (Kowalczuk et al., 2008). These proteins are required for surface expression in kidney (collectrin) and intestine (ACE2), but are also required for catalytical activity (Fairweather et al., 2015). Notably, functional expression of B^0^AT1 in mammalian cells requires high expression levels of collectrin (Danilczyk et al., 2006; Cheng et al., 2017), while only minute amounts are required in *Xenopus laevis* oocytes (Fairweather et al., 2015). The functional protein mediates the symport of 1Na^+^ together with any neutral amino acid, resulting in inward currents. The currents are small in the absence of collectrin/ACE2 (Bohmer et al., 2005; Camargo et al., 2005), but can be up to 20 times larger in their presence (Kowalczuk et al., 2008).

This knowledge was used to generate a membrane potential assay with a fluorescence readout (FLIPR), which can be applied to high throughput screens (Cheng et al., 2017; Danthi et al., 2019). High-resolution structures of B^0^AT1 homologues, such as LeuT (Yamashita et al., 2005), the *Drosophila* dopamine transporter (Penmatsa et al., 2013), and the human serotonin transporter (Coleman et al., 2016) have also allowed to generate homology models useful for *in silico* screening (Cheng et al., 2017). HTS and *in silico* screening have resulted in additional inhibitors, such as benztropine (IC_50_ 44 μM) (Cheng et al., 2017) and cinromide (IC_50_ 0.3 μM). Two of the established B^0^AT1 inhibitors were initially developed for other targets, such as COX-2 (Nimesulide) and muscarinic acetylcholine receptor (Benztropine). The target for the anticonvulsant cinromide remains unknown.

Here we describe targeted synthetic and non-targeted screening approaches to explore novel scaffolds that can inhibit B^0^AT1.

## Methods

### Cell Lines and Media

CHO-B^0^AT1-collectrin Cells (CHO-BC) were generated, cultured, and used as described recently (Cheng et al., 2017). Expression levels of B^0^AT1 in CHO-BC cells decline over time. As a result, passages <10 were used for experiments. The cells were maintained in Ham’s F-12 glutamax media with 10% (v/v) fetal bovine serum (FBS, Heat inactivated, 10082147 Gibco), 1mM glutamine, 300μg/ml hygromycin B and 275 μg/ml geneticin.

143B TK^-^ cells (human osteosarcoma cell line) were maintained in DMEM/Ham’s F-12 medium (Sigma) supplemented with 10% (v/v) FBS and 2mM glutamine. The cells were passaged at about 80–90% confluence and the medium was changed every 3–4 days. All cells were kept in a humidified incubator at 37°C and 5%CO_2_.

### Chemical Synthesis

**Figure 1:**
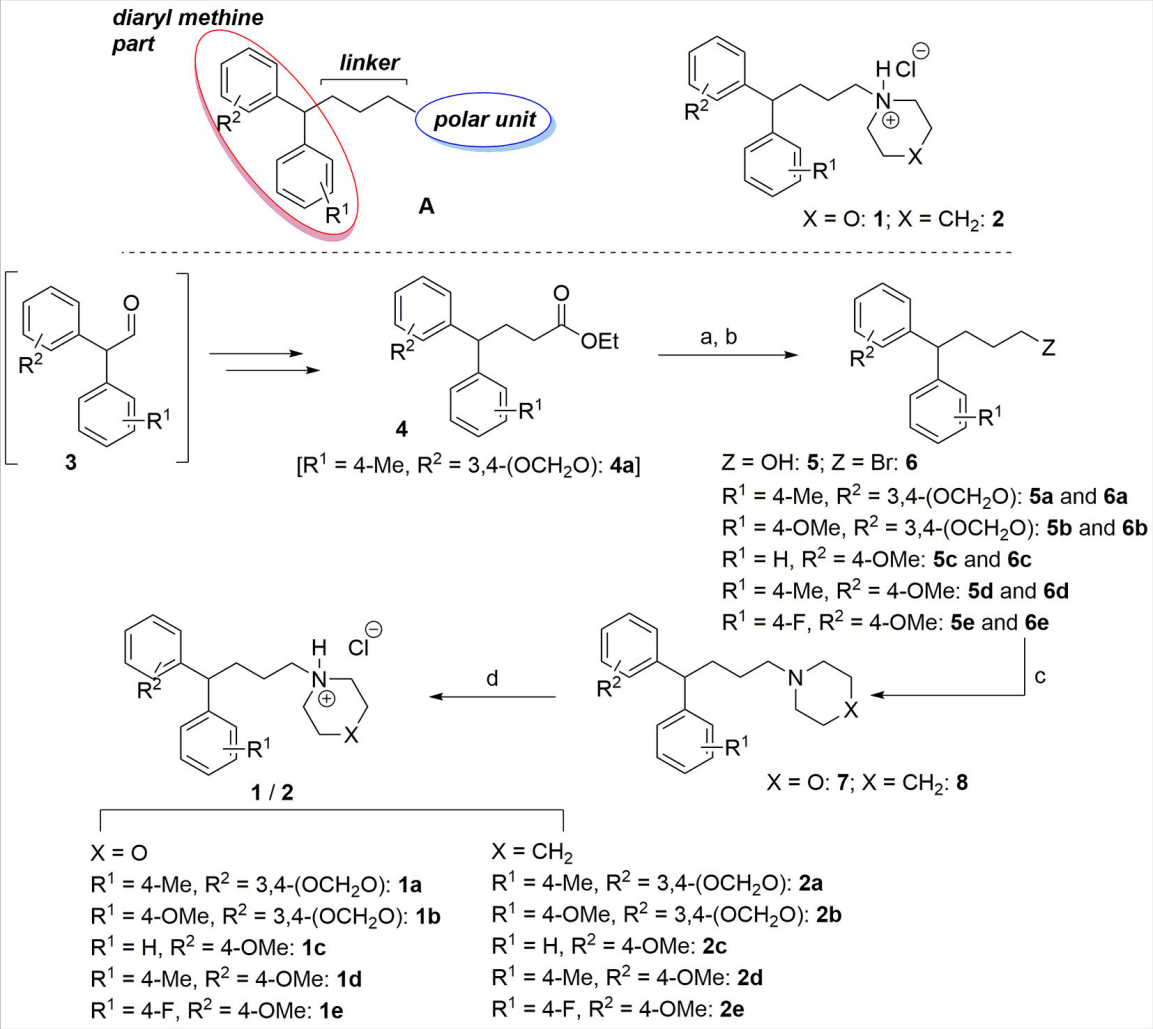
Proposed possible B^0^AT1 transporter inhibitors **1** and **2** and their synthesis. Reaction condition: (a) LiAlH_4_, THF, 0°C-rt, 6–7 h, 85–90%; (b) PPh_3_, CBr_4_, CH_2_Cl_2_, 0°C-rt, 24 h, 86–92%; (c) morpholine/piperidine, K_2_CO_3_, acetone, reflux, 20–22 h, 76–93%; (d) 4M HCl/Dioxane, CH_2_Cl_2_, rt, 24 h, 80–93%.

A schematic overview of the synthesis is shown in **Figure 1**. The general pharmacophore **A** features a *linker* connecting a *diaryl methine part* with the *polar unit,* which is either a morpholine (**1**) or piperidine (**2**) ring. Diaryl acetaldehydes **3** were utilized as starting material for a two carbon extension using a Wittig-olefination reaction, followed by a reduction reaction to form the ester derivative **4** as described previously (Tiwari et al., 2017). To replace the ester by the polar unit, compound **4a** [in the example R^1^ = 4-Me, R^2^ = 3,4-(OCH_2_O)] was first reduced using LiAlH_4_ in dry THF at 0°C, which generated 4,4-diaryl butanol-1 **5a** (85% yield). To generate the corresponding bromo-compound **6a,** a solution of the alcohol derivative **5a** in CH_2_Cl_2_ with CBr_4_ and PPh_3_ was stirred for 24 h (86% yield). Subsequently, the bromine in compound **6a** could be replaced by secondary amines, in this case morpholine and piperidine, under reflux in acetone with K_2_CO_3_ as a base. The alkylated amine products **7a** and **8a** were obtained as viscous liquids with yields of 88 and 93%, respectively. Finally, amine hydrochloride salts **1a** and **2a** were prepared by stirring amine compound **7a** and **8a** in 4M HCl in 1,4-dioxane at room temperature for 24 h (**Figure 1**), as described recently (Dubowchik et al., 2004).

Based on these optimized reaction conditions, amine hydrochloride salts **1b-e** and **2b-e**, containing variations of aryl groups at the methine center, were prepared with similar yields. All amine hydrochloride salts prepared as white solids containing racemic mixtures were pure enough to be used for biological *in vitro* assays. Identity of the compounds was verified by NMR and Mass Spectroscopy (**Supplementary Information**).

### Transport Assay Using FLIPR

Membrane depolarisation induced by amino acid transport was monitored using a FLIPR^®^ kit (Molecular Devices, R8042 BLUE DYE). Before the assay, CHO-BC cells were seeded out at a density of 60,000 cells/well and maintained in a flat transparent bottom 96-well plate (Corning) overnight. After washing three times with Hank’s balanced salt solution (136.6 mM NaCl, 5.4 mM KCl, 0.44 mM KH_2_PO_4_, 2.7mM Na_2_HPO_4_, 1.26mM CaCl_2_, 0.5mM MgCl_2_, 0.4 mM MgSO_4_, 10mM HEPES, pH 7.5, supplemented with 5 mM glucose, abbreviated HBSS + G), cells in each well were incubated for 30 min at room temperature in 50 µl of HBSS pH 7.5 containing 5 mM glucose and compounds as indicated, together with 50 µl of dye-loading buffer provided with the kit. Subsequently, the fluorescence signal was detected every 10 s before and after the addition of leucine or isoleucine at a final concentration of 1.5 mM. Fluorescence was detected at room temperature using a PerkinElmer multimode Plate Reader, with an excitation wavelength of 530 nm and emission wavelength of 565 nm.

### High Through-Put Screen (HTS)

HTS was performed with low passage CHO-BC cells on a diversity (20,000 compounds) library accessed through the Drug Discovery Centre of the Children’s Cancer Institute Australia (WECC library assembled and curated by the Walter and Eliza Hall Institute). The FLIPR assay was used with small modifications to adapt it to a 384-well format. All compounds were screened at a final concentration of 10 μM. Initial hits were confirmed, and IC_50_ determined for selected compounds. For further testing selected compounds were obtained from Chembridge (San Diego, USA) or Enamine (Kyiv, Ukkraine).

### Transport Assay Using Radiolabelled Amino Acids

CHO-BC cells with passage numbers from 3 to 10 were used in radio-labelled uptake assays. Before the assay, the cells were seeded out in 35 mm dishes (Corning) and maintained for 48–72 h until reaching 80–90% confluence. To initiate transport, the culture medium was removed and the cells washed three times with HBSS + G. Subsequently, cells were incubated with HBSS + G, radiolabelled substrates (150 μM L-[U-^14^C]leucine or 150 μM L-[U-^14^C]isoleucine) and inhibitors as indicated in the figures or table in a 37°C water bath for 6 min. To terminate transport, cells were washed three times with ice-cold HBSS pH 7.5. For measuring sodium-independent uptake, NaCl in Hank’s buffer was replaced by NMDG-Cl. Cells were then harvested by homogenizing with 500 µl of 0.1 M HCl. An aliquot of 400 µl was used for scintillation counting and the remainder for protein quantification. To investigate inhibition of ASCT2, uptake of 100 μM [^14^C]glutamine was measured in 143B cells in the absence and presence of inhibitor (at 10 μM and 50 μM). To measure inhibition of LAT1, uptake of [^14^C]leucine was measured in HBSS + G in which NaCl was replaced by NMDG-Cl in 143B cells in the absence and presence of inhibitor (at 10 μM and 50 μM).

### Transport Assay Using Sections of Inverted Intestine

For uptake assays with inverted sections of mouse small intestine, 5–7 months old C57/BL6J female mice were sacrificed by cervical dislocation (Animal ethics protocol A2016/41, Australian National University). The small intestine was removed and the intestinal lumen was rinsed with ice-cold 0.9% NaCl. Subsequently, the small intestine was inverted on a metal rod to expose the mucosa. The inverted intestine was then cut into 1 cm pieces and fitted onto enzyme spatulas, which were immersed in HBSS pH 7.5 supplemented with 1 mM glutamine and protease inhibitor (Roche Complete). After washing twice in modified HBSS pH 7.5 (containing 10% NaCl + 90% NMDG-Cl for Na^+^-dependent uptake and 100% NMDG-Cl for Na^+^-independent uptake, both buffers supplemented with 1 mM glutamine) to remove residual compounds, the segments were pre-incubated in the same buffer in the presence of 50 µM of transport inhibitor and protease inhibitors for 15 min. At the end of the pre-incubation, the segments were transferred for 30 s into uptake buffer at 37°C (HBSS pH 7.5 supplemented with 150 μM [^14^C]leucine and 50 µM transport inhibitor). For Na^+^-dependent uptake regular HBSS was used, for Na^+^-independent uptake NaCl was replaced by NMDG-Cl. Subsequently, the segments were rinsed three times in ice-cold HBSS pH 7.5 and lysed in 400 µl of 10% SDS. After 2–3 h, the lysed tissues were analyzed by scintillation counting.

### Homology Model and Docking Protocol

The B^0^AT1 homology model was generated using the SWISS-MODEL server and the LeuT outward open (PDP: 3TT1) and occluded structures (PDB: 2A65). The peptide sequences of B^0^AT1 and LeuT show 24% identical residues and 40% similar residues. Similarity in transmembrane domains is significantly higher than in loops. Only 3 amino acids differ around the substrate binding site. The two homology models were validated by comparing to models generated by various homology modelling programs (HHpred, MODELLER, Phyre2, I-TASSER). No significant difference was observed between the structural alignments of these models. Unrestrained equilibration was conducted for 200 ns in a neutral system with the transporter in a 1-palmitoyl-2-oleoylglycerophosphocholine (POPC) bilayer surrounded by water (TIP3P) and 150 mM NaCl.

AutoDock Tools (Morris et al., 2009) incorporating the Gasteiger PEOE method (Gasteiger and Marsili, 1980) was used to assign partial charges to all atoms in the LeuT-based outward-open B^0^AT1 structure, LeuT-based occluded structure and to all test-ligand structures. Since AutoDock does not explicitly support the Na^+^ atom type, parameters were approximated to Ca^2+^. Charge for Na^+^ was manually set to +1. For docking the outside open homology model was used without equilibration. Otherwise, one sodium ion would depart from the binding site of the outside open transporter. Moreover, the outside open state would tend to converge towards the occluded state as no substrate or inhibitor was present. Using AutoGrid, the docking search space was set to the region in between and inclusive of the Na^+^/substrate binding site 1 and the Arg/Asp gates at the top of the binding pocket. All docking runs were carried out locally using AutoDock 4.2.

### Animal Handling

Animal experiments were approved by the Animal experimentation ethics committee of the Australian National University (A2016/41). Both male and female C57/Bl6J mice (age 6–8 weeks) (Jiang et al., 2015) were held in individually ventilated cages (<5 animals per cage) under specified pathogen-free conditions under a 24 h light/dark cycle. After one week of acclimatization, C57Bl6J mice (n = 6) and SLC6A19ko mice (n = 6) were placed on a high fat diet plus high fructose/glucose (23.1g/L fructose and 18.9g/L glucose in drinking water) for 4 months to accelerate the development of non-alcoholic steatohepatitis (Liu et al., 2018). As a control, 5 C57Bl6J mice were given standard chow diet and plain water. The body weight and diet consumption were monitored twice weekly and general health was checked daily. Mice were sacrificed after 4 months by cervical dislocation and liver samples were collected for histopathological analysis.

### Liver Histopathological Analysis

Fresh mouse liver was fixed in 10% neutral buffered formalin, embedded into wax and 4 µm thick sections were evaluated for the development of NASH after staining in hematoxylin and eosin (H&E). Scores were given for steatosis (0–3), lobular inflammation (0–3), and ballooning (0–2). The pathologist was blinded to the treatment group.

### Statistical Analysis

Data are shown as mean ± S.D., transport activities indicated as Na^+^-dependent net uptake, were calculated by subtracting the uptake activity in the absence of Na^+^ from the total uptake rate. The fluorescent signals measured in the FLIPR assay were normalized to baseline readings before the addition of substrates. Data are presented as percentage of baseline at each time point. IC_50_ was calculated using the equation: $y=y_{\max}\left(1-\frac{\left[x\right]}{[x]+IC_{50}}\right)$ . The number of experimental (e) and technical (n) repeats is indicated in the figure legends.

## Results

### Reduced Steatosis in SLC6A19ko Mice

**Figure 2:**
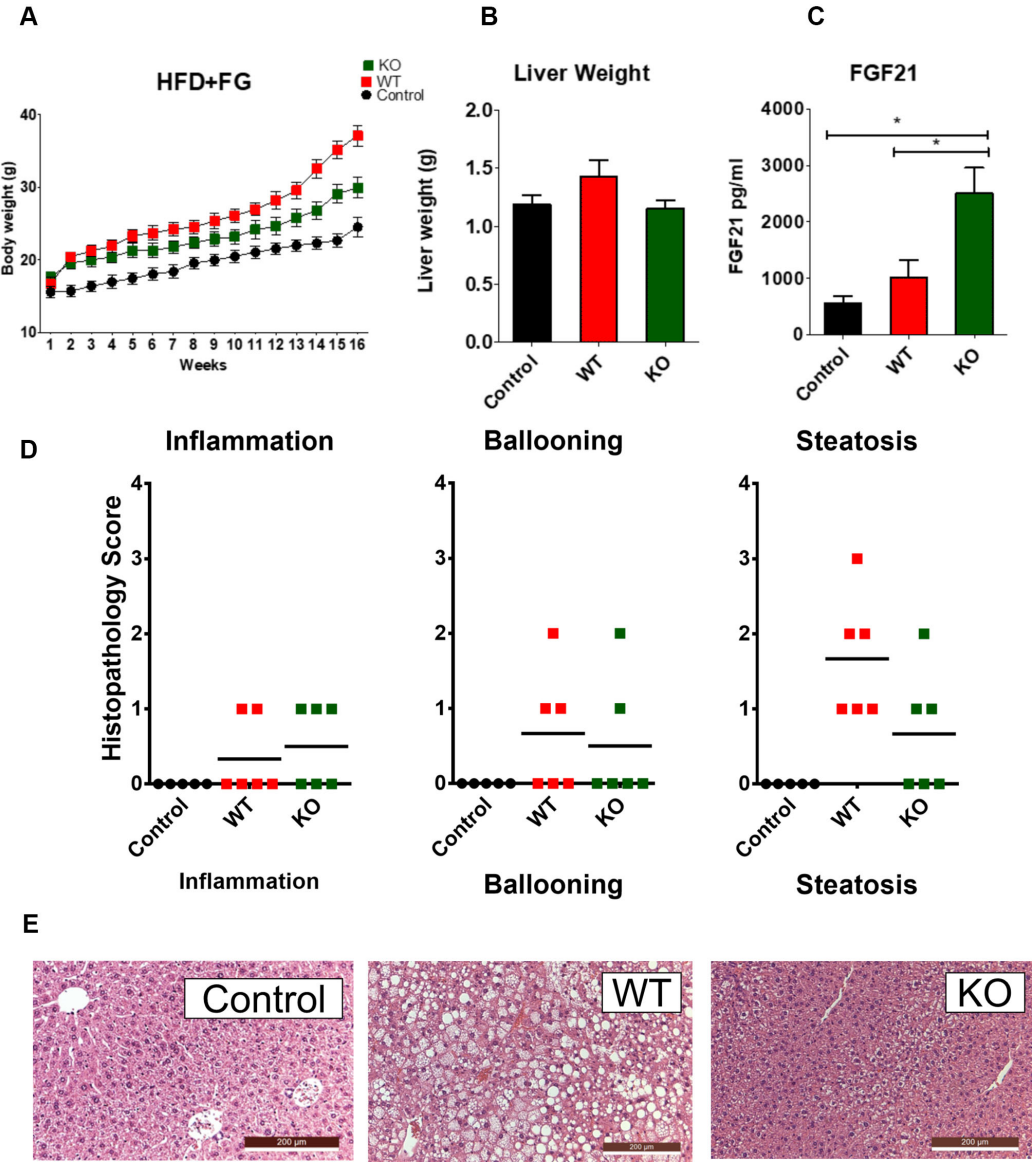
Protection against non-alcoholic steatohepatitis in SLC6A19ko mice. Wildtype (C57Bl6/J) and SLC6A19ko mice (in the same genetic background) were placed on a high-fat/fructose diet for 16 weeks. Body weight was monitored twice weekly **(A)**. Animals were sacrificed after 16 weeks for measurement of liver weight **(B)**, plasma levels of FGF21 **(C)**, and liver histological analysis **(D**, **E)**. To quantify steatohepatitis, scores were given for tissue inflammation, ballooning of hepatocytes and steatosis **(D)**. Sample sections, stained with haematoxylin and eosin, are shown for each experimental group **(E)**. Animals are displayed as individual data points. *different from control at p < 0.05.

Previously, we showed that liver triglycerides were significantly reduced in SLC6A19ko mice, most likely due to conversion into ketone bodies (Jiang et al., 2015). To investigate whether this mechanism protected against development of NASH, we placed animals on a high fat and fructose/glucose supplemented diet for 16 weeks and analyzed liver anatomy and histology at the end of the experiment (**Figure 2**). SLC6A19ko mice gained less weight on the diet (**Figure 2A**) and liver weight was similar to that of wild-type mice on a control diet (**Figure 2B**). The high-fat diet induced production of FGF21 in both animal groups, but SLC6A19ko animals produced about 2.5-times more FGF21 than wild-type animals (**Figure 2C**). Histological analysis revealed lower scores for SLC6A19ko mice, some of which were indistinguishable from mice on a control diet (**Figures 2D, E**). The diet intervention caused limited inflammation and cell death (ballooning) in both groups, but steatosis was pronounced in wild-type animals and reduced in SLC6A19ko animals (p = 0.06). These results extend our previous observations and provide the rationale for the development of specific and selective B^0^AT1 inhibitors.

### Diarylmethine Compounds as inhibitors of B^0^AT1

**Figure 3:**
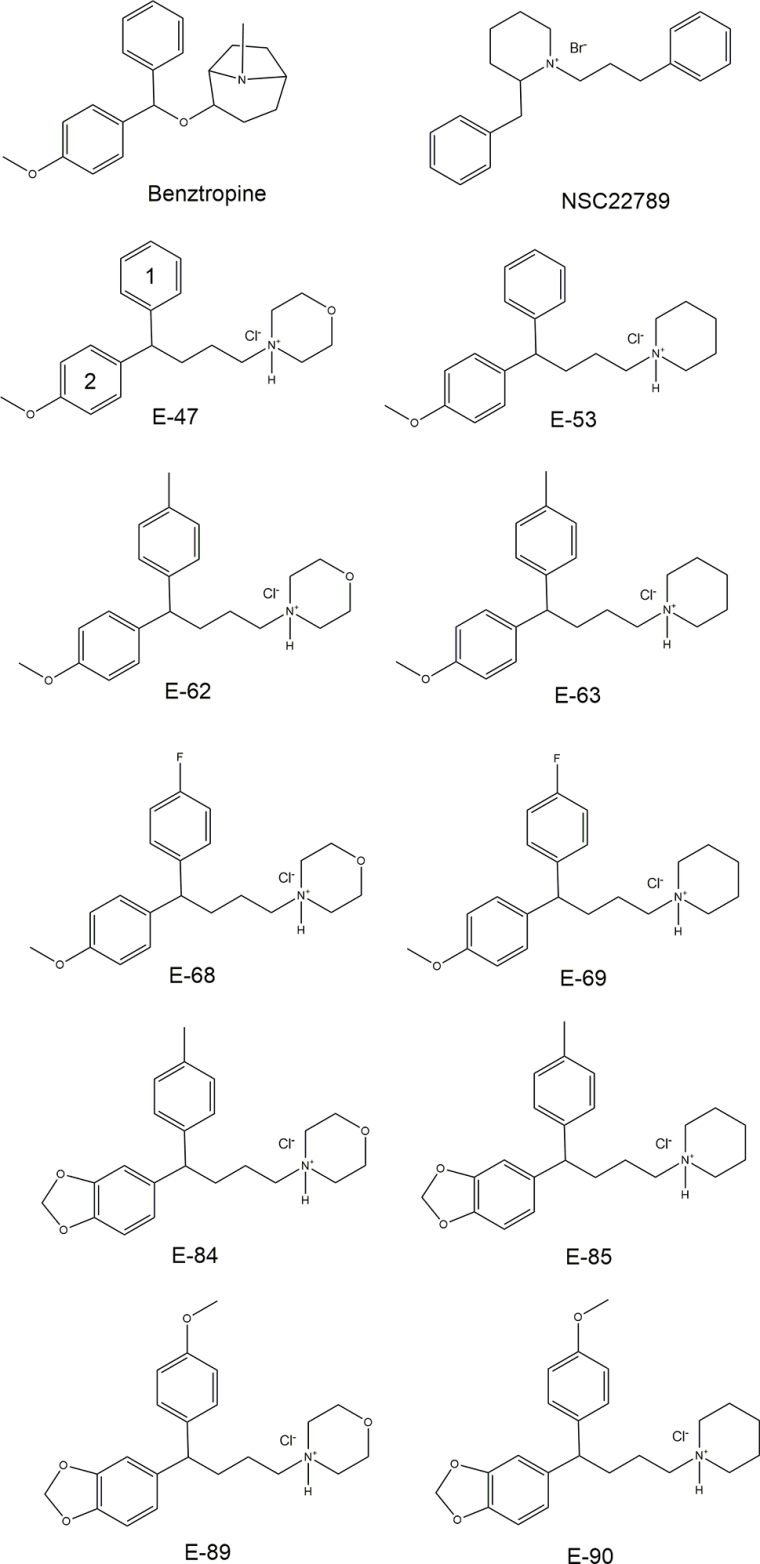
Structural comparison of proposed B^0^AT1 inhibitors with previously identified inhibitors Benztropine and NSC22789. Functional characterisation through inhibition of B^0^AT1 (IC_50_) in two different assays is documented in **Table 1**.

Previously we identified a group of novel B^0^AT1 inhibitors through *in silico* screening, exemplified by NSC22786 and benztropine (**Figure 3**). These compounds share certain characteristics such as two aromatic rings, and a tertiary nitrogen incorporated into an aliphatic ring structure. Based on these properties we designed a series of chemical homologues with systematic variation of the basic pharmacophore. The resulting group of 10 compounds was tested using FLIPR assay and radioactive uptake assays. The structures of these compounds are shown in **Figure 3** and the corresponding IC_50_-values in both assays are listed in **Table 1**. Inspection of the IC_50_-values shows that compounds E-89 and E-90 performed relatively poorly and shared a methoxy-group in para position of aromatic ring 1. Interestingly, the best performing compounds E-47, E-62, and E-53 have a methoxy-group on aromatic ring 2. Given that the diarylmethine domain can rotate freely, it appears beneficial to have one aromatic ring without substitution and one with, a property shared by benztropine. Polar substituents on both aromatic rings further increases IC_50_-values. E-85 does not conform to this basic structure, but only performed well in the flux assay. There is no clear discrimination between a piperidine or morpholine-based structure of the polar domain, suggesting that the positively charged nitrogen is most critical. Overall the new B^0^AT1 inhibitors had a slightly better IC_50_ than NSC22789, but were comparable to benztropine, which is readily commercially available. In summary, systematic variation of a scaffold based on overall similarity to benztropine and NSC22789 produced inhibitors of similar quality as benztropine, but did not generate structures that were a significant improvement.

**Table 1:** Inhibition of B^0^AT1 by modified diarylmethines. All compounds were tested using a membrane potential detecting FLIPR assay and a classic radioactive flux assay.

Compound	IC_50_ (FLIPR) μM	IC_50_ (Flux) μM
Benztropin	44 ± 9	71 ± 8
NSC22789	90 ± 21	78 ± 16
E-47	21 ± 6	17 ± 9
E-53	31 ± 15	8 ± 4
E-62	20 ± 1	16 ± 8
E-63	32 ± 14	39 ± 10
E-68	33 ± 9	19 ± 6
E-69	94 ± 20	46 ± 7
E-84	31 ± 3	19 ± 4
E-85	36 ± 11	8 ± 2
E-89	39 ± 4	34 ± 7
E-90	74 ± 19	25 ± 8

### Identification of New B^0^AT1 Inhibitors by HTS

**Figure 4:**
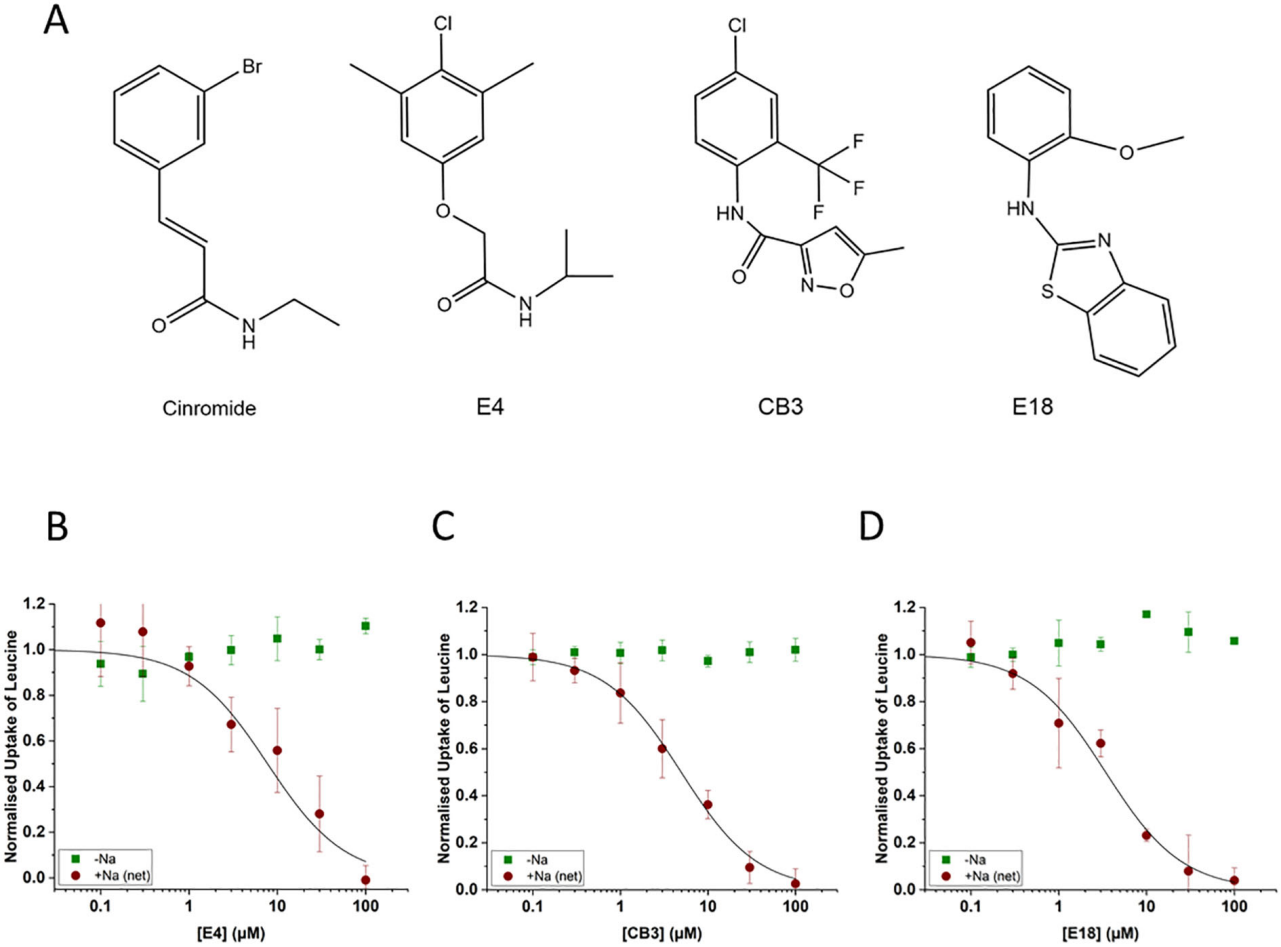
Properties of second-generation inhibitors of B^0^AT1. Inhibitors E4, CB3 and E18 were identified by high-throughput screening. The established B^0^AT1 inhibitor cinromide is shown for comparison **(A)**. Inhibition of B^0^AT1 activity [red symbols **(B–D)**] was tested in CHO-BC cells using a FLIPR assay (n = 3, e = 3). The assay allows to test the specificity of the inhibitors against the endogenous LAT1 transporter [green symbols **(B–D)**].

To identify novel scaffolds with better properties, we performed a systematic screen of a 20,000-compound diversity library at a final concentration of 10 μM. We previously established a HTS and cell line for automated screening of large libraries (Cheng et al., 2017). The screen resulted in 50 compounds for which an IC_50_ was determined. Further testing revealed three compounds (named CB3, E4 and E18) that were investigated in more detail (**Figure 4** and **Table 2**). These compounds inhibited B^0^AT1 mediated isoleucine transport with an IC_50_ of 1.9–13.7 μM. To further evaluate the efficacy of our inhibitors, we compared them to cinromide, which inhibited B^0^AT1 with an IC_50_ of 0.5 μM, in line with data reported recently (Danthi et al., 2019). Notably, cinromide is structurally similar to E4.

**Table 2:** Inhibition of B^0^AT1 by second generation inhibitors. All new compounds were tested using a membrane potential detecting FLIPR assay and a classic radioactive flux assay.

Compound	IC_50_ (FLIPR) µM	IC_50_ (Flux) µM
CB3	1.9 ± 0.6	4.9 ± 0.4
E4	13.7 ± 0.9	7.7 ± 1.9
E18	4.2 ± 0.9	3.4 ± 0.3
Cinromide	0.5 ± 0.08	n.d.

### Characterisation of Novel B^0^AT1 Inhibitors

CHO-BC cells stably express human B^0^AT1 and collectrin. When leucine or isoleucine are used as substrates, they are transported by B^0^AT1 and the endogenous neutral amino acid transporter LAT1. Both transporters are of similar capacity, but are easily discriminated because LAT1 is Na^+^-independent, while B^0^AT1 is Na^+^-dependent. The data in **Figure 4** clearly demonstrate that all three inhibitors are selective for B^0^AT1 (red symbols) in this assay, while the endogenous LAT1 remains fully active (green symbols).

**Figure 5:**
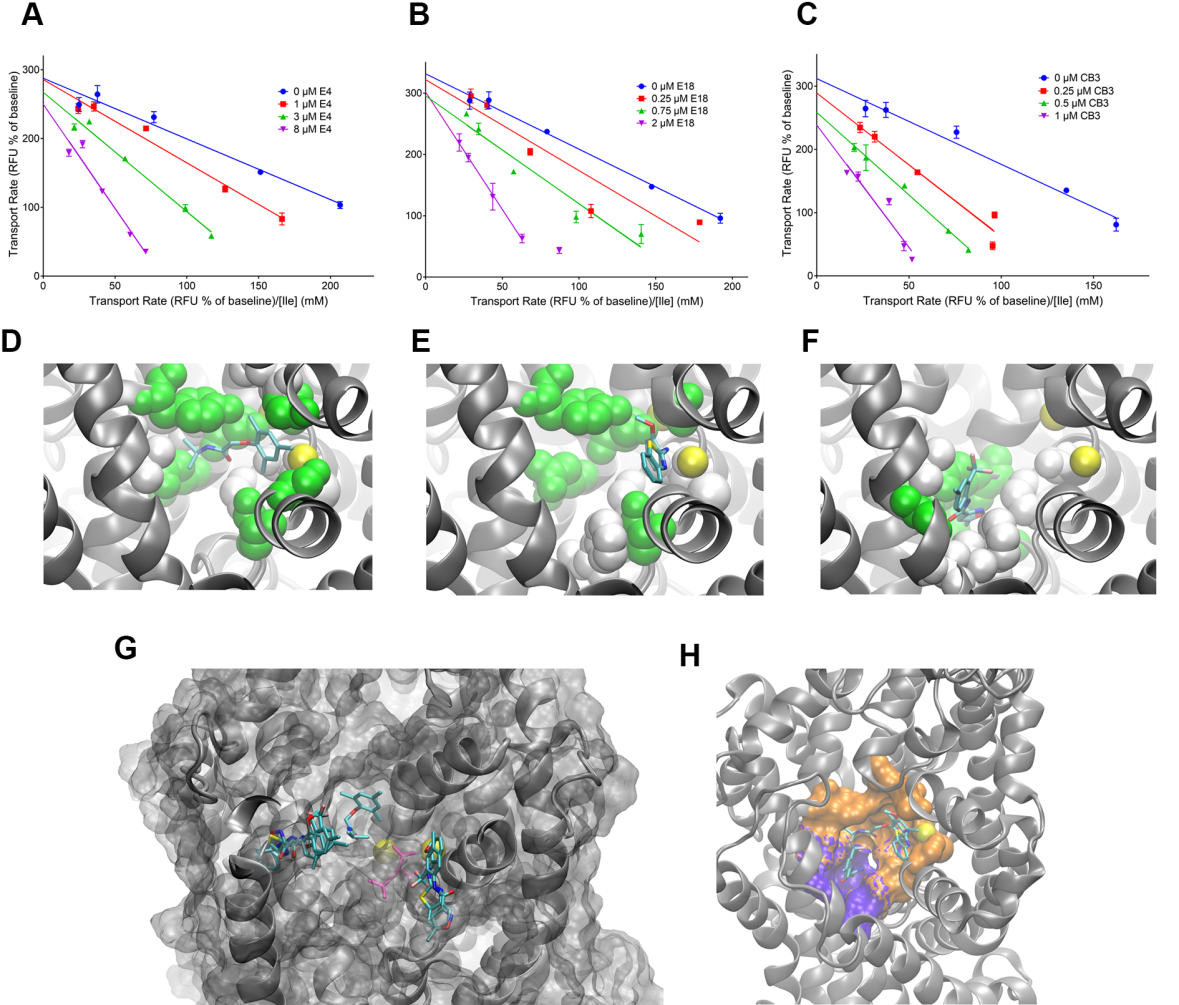
Mechanistic aspects of B^0^AT1 inhibitors. Inhibition of B^0^AT1 by compounds E4 **(A)**, E18 **(B)** and CB3 **(C)** (n = 3, e = 3) was determined by FLIPR assay in CHO-BC cells. To determine the mode of inhibition, the kinetic properties of leucine uptake were determined in the presence of three different inhibitor concentrations. Docking poses of compounds E4 **(D)**, E18 **(E)** and CB3 **(F)** are shown for comparison. Amino acid side-chains of the binding pocket are shown in green, cosubstrate Na^+^_1_ is shown in yellow (foreground), Na^+^_2_ is only partially visible in the background and may not exist in B^0^AT1. **(G)** Docking of E4, E18, and CB3 to the outside occluded conformation. Leucine is shown in magenta in the orthostatic binding site for comparison, the site was unoccupied during docking of the inhibitors. **(H)** Docking to the outside-open conformation suggests the presence of an orthostatic (orange) and allosteric binding site (blue) in the wider substrate binding cavity of B^0^AT1.

To understand the mode of inhibition, we determined K_M_-values at three different inhibitor concentrations using FLIPR assay and plotted Eadie-Hofstee transformed data (**Figure 5**). Fully parallel lines indicate a change of V_max_ only, while fully converging lines (at x = 0; y = Vmax) indicate a change of K_M_ only with increasing concentrations of the inhibitor. A classic competitive inhibitor only increases the K_M_ of the substrate, but does not affect V_max_ (converging lines), classic allosteric inhibitors inhibit the turnover of the transporter without affecting substrate binding (parallel lines). The data suggested a largely competitive mode of inhibition for compounds E4 (**Figure 5A**) and E18 (**Figure 5B**) and a more mixed model for CB3 (**Figure 5C**). To rationalize the mode of inhibition, we performed docking studies using a homology model of B^0^AT1 based on the outside-open conformation of LeuT. The outside-open conformation is the most likely state of the transporter to bind inhibitors, and in this state the binding site is significantly larger than in the occluded conformation allowing multiple binding poses, of which the most frequent pose is depicted (**Figures 5D–F**). In the homology structure the two Na^+^ are shown as yellow spheres. Both, LeuT Na^+^-site 1 and -site 2 are conserved in B^0^AT1, but only one site is used functionally. This is most likely site 1 as it binds the α-carboxyl-group of the amino acids (O’mara et al., 2006). None of the inhibitors had a carboxyl-group to interact with Na^+^_1_. Instead, compounds E4 and E18 showed a cation-pi interaction with Na^+^_1_ and thus are likely to compete with a substrate amino acid (**Figures 5D, E**). Compound CB3 had its most likely pose further away from Na^+^_1_, suggesting a possible non-competitive mode of inhibition (**Figure 5F**). Many transport inhibitors are substrate analogues that are too large to fit into the occluded substrate binding cavity, thereby blocking the conformational change required for transport (Broer, 2018). In docking studies with a homology model of the LeuT occluded structure we could not compute a docking score for E4, E18, or CB3 at the substrate binding site (**Figure 5G**). Instead the compounds preferred binding to different sites in the vestibule, including the proposed Substrate-binding site 2 (Shi et al., 2008), which was the preferred docking site for compound E4. In the outside open conformation, the volume of the binding site increases, encompassing an orthostatic binding site (**Figure 5H**, orange), occupied by E4, E18, or the substrate and an allosteric binding site (**Figure 5H**, blue), which is compatible with substrate binding.

**Figure 6:**
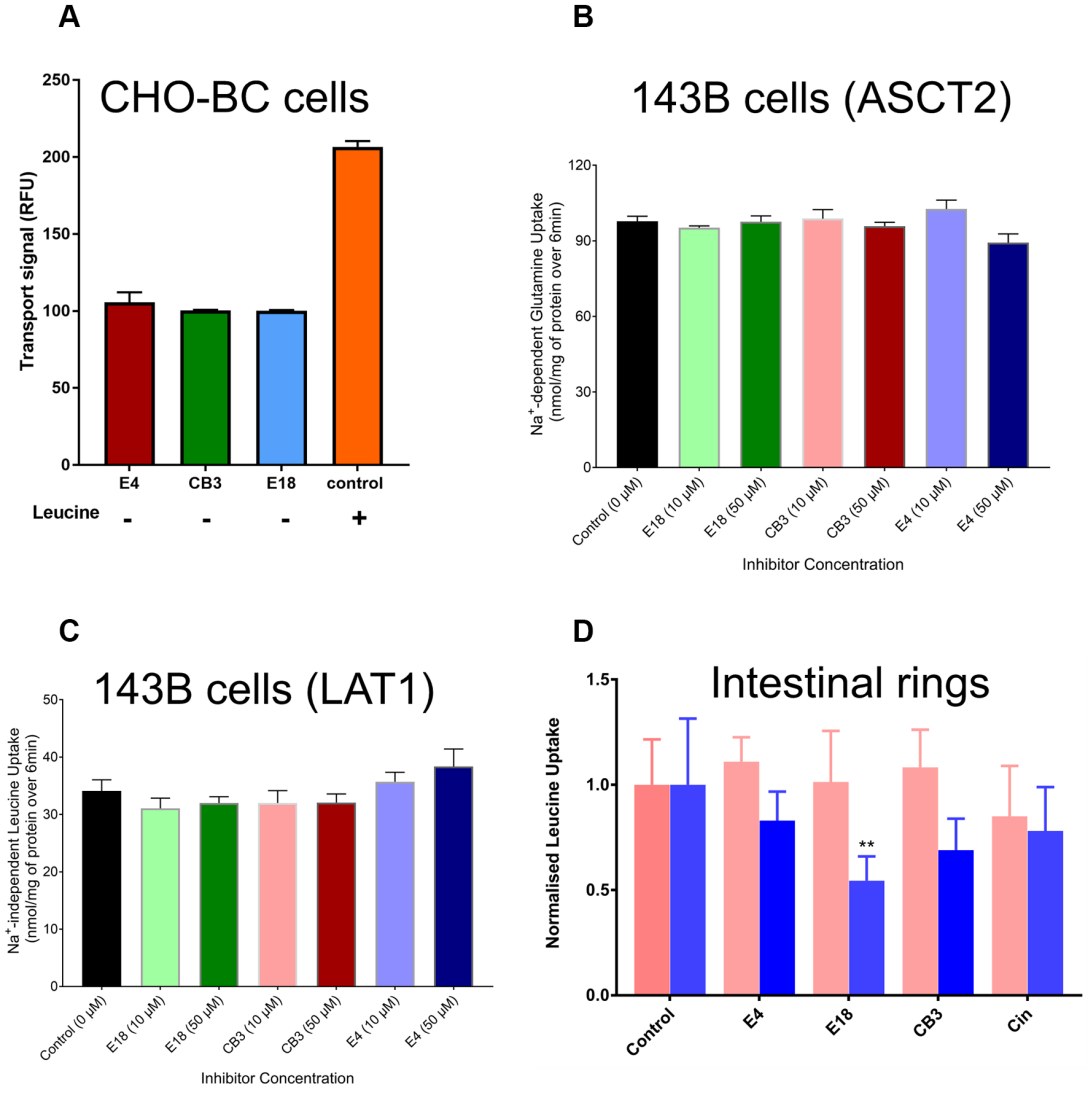
Specificity and efficacy of B^0^AT1 inhibitors. When tested alone, compounds E4, E18, and CB3 (30 μM, n = 3, e = 2) did not increase fluorescence above the baseline (100 RFU) in the FLIPR assay in CHO-BC cells **(A)**. Although structurally unrelated to the substrates of the transporter, compounds E4, E18, and CB3 are highly specific. They failed to inhibit Na^+^-dependent glutamine uptake in 143B cells, which is largely mediated by ASCT2 **(B)** or Na^+^-independent leucine uptake in 143B cells, which is mediated by LAT1 **(C)** (n = 3, e = 3). Panel **(D)** shows the potential of E4, E18, and CB3 (50 μM) to inhibit Na^+^-dependent (B^0^AT1, blue bars) and Na^+^-independent (rbat/b^0,+^AT salmon coloured bars) uptake of leucine in inverted sections of mouse intestine (n = 3, e = 3); **p < 0.01 as compared to the control.

The lack of a docking score in the occluded substrate binding site suggests that the three compounds do not generate sufficient weak bonds to trigger a conformational change of the transporter. This was supported by FLIPR assay with inhibitors in the absence of substrate, which failed to elicit a transport signal in CHO-BC cells (**Figure 6A**).

As shown above the three inhibitors did not inhibit the endogenous LAT1 transporter. To investigate the specificity of the compounds further, we tested them in human 143B osteosarcoma cells, which we have extensively characterised previously and used for pharmacological analysis (Broer et al., 2018). Na^+^-dependent glutamine transport in 143B cells is largely mediated by ASCT2 (SLC1A5). Na^+^-independent transport of leucine, by contrast, is mediated entirely by LAT1. When tested at two concentrations above the IC_50_ neither of the compounds inhibited Na^+^-dependent glutamine transport (**Figure 6B**), suggesting selectivity against ASCT2. Similarly, neither compound inhibited Na^+^-independent transport of leucine (**Figure 6D**), suggesting selectivity against LAT1.

To test whether the novel B^0^AT1 inhibitors block the transporter in a more physiological environment, we studied leucine uptake in inverted sections of mouse intestine (**Figure 6C**). We established previously that Na^+^-dependent leucine uptake (blue bars) in this system is mediated by B^0^AT1 (Broer et al., 2011). In addition leucine uptake has a Na^+^-independent transport component (salmon coloured bars), which is most likely mediated by rbat/b^0,+^AT. Because of variability between animals, all data were normalized to uninhibited controls from the same animal. Compound E4 and the structurally related cinromide showed limited capacity to inhibit B^0^AT1 in this *ex vivo* preparation, whereas E18 and CB3 were more potent.

In summary we have confirmed previously identified scaffolds as inhibitors of B^0^AT1 and in addition present novel scaffolds with better inhibitory capacity in an *ex vivo* preparation.

## Discussion

In this study we extend previous observations (Jiang et al., 2015) that lack of SLC6A19 reduces liver triglycerides, most likely by conversion into ketone bodies. Most SLC6A19ko animals showed a significant protection against NASH, suggesting a potential of B^0^AT1 inhibitors to treat this metabolic disorder. Moreover, they could also be used to remediate amino acid imbalance in rare inherited disorders such as phenylketonuria (Belanger et al., 2018).

In a previous study we developed a membrane potential-sensing fluorescent assay (FLIPR) that is suitable for high through put screening of B^0^AT1 inhibitors (Cheng et al., 2017). In this study we used this assay to screen a diversity library of 20,000 compounds (WECC) that has been carefully curated to cover a diverse range of chemical scaffolds and at the same time excludes chemical substructures with high reactivity and other undesirable properties (Lackovic et al., 2014). Hits were further characterised using FLIPR and radioactive uptake assays. Both methods gave comparable results. Due to the workflow of the FLIPR assay compounds are pre-incubated for 30 min with the cells, which can result in lower IC_50_-values (e.g. **Table 1**). Since the FLIPR provides an indirect signal of transport we generally consider flux assays to be the gold standard for inhibitor characterisation. In a screen performed by a team from Sanofi/Genzyme, cinromide and related compounds were identified in a library of 3,440 compounds (Danthi et al., 2019). We confirmed the efficacy of cinromide in our assay, allowing us to compare it to the leads derived from our HTS. The suitability of the WECC library is illustrated by the identification of compound E4, which is largely similar to cinromide. Cinromide and E4 have a pseudo peptide bond, which combines elements of the amino and carboxyl-group of the genuine substrates of the transporter. Although some similarity can be construed, compounds CB3 and E18 present significantly different pharmacophores. Nevertheless, an aromatic ring and a secondary or tertiary amine can be found in all compounds tested in this study. Although these elements are also found in benztropine, it appears that a single aromatic ring is preferred over the diarylmethine structure. Notably a single aromatic ring is structurally closer to native substrates of the transporter, such as phenylalanine and tyrosine. The α-carboxyl-group of the amino acid substrates is essential for ion coordination of the Na^+^-cotransport mediated by LeuT (Yamashita et al., 2005). The lack of a carboxyl-group in any of the compounds could be a key factor of their inhibitory action. Notably we were unable to compute a docking score for any of the compounds using a homology model of the occluded transporter, potentially because they fail to coordinate Na^+^_1_. Compounds E4 and E18 are nevertheless likely to compete with the substrate due to a cation-pi interaction with Na_1_ in the outside open conformation, which would interfere with substrate binding. We did not analyze the effect of our lead compounds on Na^+^-binding, because Na^+^-Km of B^0^AT1 changes with substrate concentration (Bohmer et al., 2005) and as a result it would be difficult to see whether changes of the Na^+^-Km would be caused by lack of ion coordination or displacement of substrate.

In addition to competing at the substrate binding site, a second inhibitor binding site has been identified in the serotonin transporter (Coleman et al., 2016). The two sites have been labelled orthostatic, i.e. where the substrate is located and allosteric, i.e. in the vicinity of the orthostatic site and were also detected in the GABA/betaine transporter BGT1 (Jorgensen et al., 2017). Transport inhibitors, in contrast to enzyme inhibitors bind to the ground state of the transporter, i.e. the outside open conformation. The transition state of the transporter is formed by an induced fit around the substrate (Kramer, 1994; Broer, 2018). Transport inhibitors prevent the adoption of the transition state either by being too bulky (Singh et al., 2008) or by providing insufficient interactions to reduce the activation energy to form the occluded state. The latter is illustrated here by the lack of a carboxyl-group. The allosteric site allows relatively compact molecules to block assumption of the transition state, because it is located in the vestibule, which closes during the adoption of the transition state (Penmatsa and Gouaux, 2014). Due to the chemical difference to the orthostatic binding site, compounds can be chemically quite different to the substrate. Accordingly, rational drug design based on substrate analogues is more likely to generate competitive inhibitors, while HTS, particular when performed in the presence of high substrate concentrations, is more likely to generate allosteric inhibitors. This is an important consideration for drugs that need to bind in the intestinal environment, where amino acid concentrations are high after nutrient intake. The bioavailability of the three lead compounds remains to be determined. The competitive and instantaneous mode of inhibition suggests that compounds do not enter the cells to inhibit the transporter. Moreover, none of the compounds was a substrate of the B^0^AT1. This does not exclude absorption in the intestine by other transporters, a prerequisite for inhibition of renal B^0^AT1. In summary, this second generation of B^0^AT1 inhibitors are promising compounds to modulate amino acid transport in epithelial cells but need to be further improved to achieve the required efficacy *in vivo*.

## Data Availability Statement

The raw data supporting the conclusions of this article will be made available by the authors, without undue reservation, to any qualified researcher.

## Ethics Statement

The animal study was reviewed and approved by the Animal Experimentation Ethics Committee at the Australian National University.

## Author Contributions

AY, NS and QC performed all pharmacological experiments, analyzed data, and edited the manuscript. PT synthesized and analyzed chemical compounds. KJ performed the nutrition experiments and analyzed data. IA designed chemical compounds and edited the manuscript. SB developed the study design, analyzed data and wrote the manuscript. AY and NS contributed equally to this study.

## Funding

Work in the laboratory of the authors was funded by Australian Research Council Grant: DP180101702 and National Health and Medical Research Council Grant: 1128442. The high-throughput screen was funded by a Grant from Diabetes Australia.

## Conflict of Interest

The authors declare that the research was conducted in the absence of any commercial or financial relationships that could be construed as a potential conflict of interest.

## Abbreviations

HBSS, Hank’s balanced salt solution; HTS, High through put screening; NASH, non-alcoholic steatohepatitis; PKU, phenylketonuria.
