# Corrigendum: Novel chemical scaffolds to inhibit the neutral amino acid transporter B^0^AT1 (SLC6A19), a potential target to treat metabolic diseases

**DOI:** 10.3389/fphar.2024.1485054

**Published:** 2025-03-07

**Authors:** Aditya Yadav, Nishank Shah, Praveen Kumar Tiwari, Kiran Javed, Qi Cheng, Indrapal Singh Aidhen, Stefan Bröer

**Affiliations:** ^1^ Research School of Biology, Australian National University, Canberra, ACT, Australia; ^2^ Department of Chemistry, Indian Institute of Technology Madras, Chennai, India

**Keywords:** phenylketonuria, steatohepatitis, non-alcoholic steatohepatitis, solute carrier, high throughput screening, HTS

In the published article, there was an error in Figure 4 as published. The displayed structure of compound E4 in the article and on the Enamine site (Catalog ID T5320580) is that of (2-(4-chloro-2,6-dimethylphenoxy)-*N*-isopropylacetamide). Subsequent research showed that this compound is largely inactive as an inhibitor (Xu et al., 2024), while it was evaluated in the original high throughput screen as a potent inhibitor. An IC_50_ of 13.7 µM (FLIPR assay) was determined with the ordered compound as shown in **Table 2** of the original article and confirmed by radioactive flux assay (IC_50_ 7.7 µM). To resolve the discrepancy, we performed structural analysis and showed that the compound in the HTS collection was in fact (2-(4-chloro-3,5-dimethylphenoxy)-*N*-isopropylacetamide). The corrected Figure 4 and its corrected caption (‘Properties of second-generation inhibitors of B^0^AT1. Inhibitors E4, CB3 and E18 were identified by high-throughput screening. The established B^0^AT1 inhibitor cinromide is shown for comparison **(A)**. Inhibition of B^0^AT1 activity [red symbols **(B–D)**] was tested in CHO-BC cells using a FLIPR assay (n = 3, e = 3). The assay allows to test the specificity of the inhibitors against the endogenous LAT1 transporter [green symbols **(B–D)**].') appear below. The docking experiment presented in **Figure 5D** remains correct, although it was performed with (2-(4-chloro-2,6-dimethylphienoxy)-N-isopropylacetamide). Subsequent research has, however, shown that the active compound binds to an allosteric site on the transporter (Xu et al., 2024). To avoid confusion, we have since renamed the active compound JX98.

**FIGURE 4:**
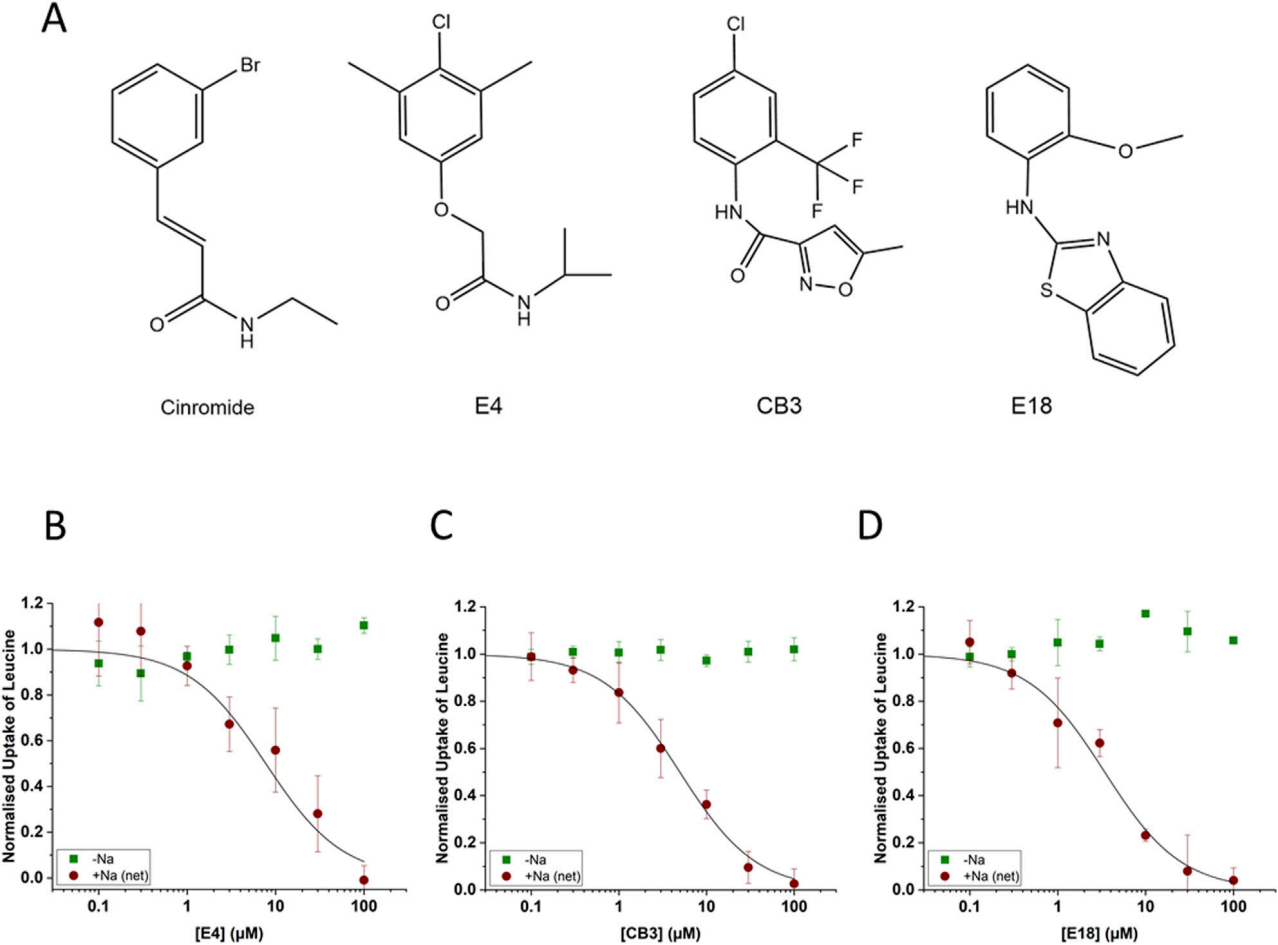
Properties of second-generation inhibitors of B^0^AT1. Inhibitors E4, CB3 and E18 were identified by high-throughput screening. The established B^0^AT1 inhibitor cinromide is shown for comparison **(A)**. Inhibition of B^0^AT1 activity [red symbols **(B–D)**] was tested in CHO-BC cells using a FLIPR assay (n = 3, e = 3). The assay allows to test the specificity of the inhibitors against the endogenous LAT1 transporter [green symbols **(B–D)**].

The authors apologize for this error and state that this does not change the scientific conclusions of the article in any way. The original article has been updated.
