# Supplementary material for: Novel Chemical Scaffolds to Inhibit the Neutral Amino Acid Transporter B0AT1 (SLC6A19), a Potential Target to Treat Metabolic Diseases
Source: Front Pharmacol. 2020 Feb 28;11:140. doi: 10.3389/fphar.2020.00140 (PMC7059793; doi:10.3389/fphar.2020.00140)
Supplement: Supplementary file 1 [file DataSheet_1.pdf]

Experimental Section:

General information: All reactions were carried out in oven dried glassware. Solvents used for column chromatography were LR grade. Thin layer chromatography was performed on aluminium plates coated with silica gel 60. Visualization was observed by U.V. light or dipping into 2,4-Dinitrophenylhydrazine solution or by dipping into a solution of cerium (IV) sulphate (2.5 g) and ammonium molybdate (6.25 g) in 10% sulphuric acid (250 ml) followed by charring on a hot plate. ^{1}H NMR (400 MHz/500MHz) and ^{13}C NMR (100MHz/125MHz) spectra were recorded in chloroform-*d* ($CDCl_3$) or DMSO-d_6 and chemical shifts are given in part per million (ppm). ^{1}H NMR spectra were referenced to $CDCl_3$ (7.26 ppm) and DMSO-d_6 (2.5 ppm) whereas ^{13}C NMR spectra were referenced to the central line of $CDCl_3$ (77.16 ppm) and DMSO-d_6 (39.50 ppm). The multiplicity are given as, s = singlet, d = doublet, t = triplet, dd = doublets of doublet, m = multiplet and coupling constants J are reported in Hz. IR was determined by JASCO-FT/IR-4100 Spectrometer using NaCl cell. HRMS were recorded on a MICRO-QTOF mass spectrometer by using the ESI technique at 10 eV.

Synthesis of compound 5: The solution of compound **4a** (0.36 g, 1.09 mmol) in dry THF (4 mL), was added slowly to 2 mL suspension of $LiAlH_4$ (83 mg, 2.18 mmol) under N_2 at 0 °C and stirred for 6-7 h. After complete consumption of staring material, reaction was quenched by slow addition of sat aq. solution of NH_4Cl at 0 °C and aq. layer was extracted by EtOAc (3 x 5 mL). All organic layers were collectively dried over Na_2SO_4, concentrated and crude product was purified through silica gel column chromatography using EtOAc:hexanes (1:4) as eluent, which afforded compound **5a** as colorless viscous liquid.

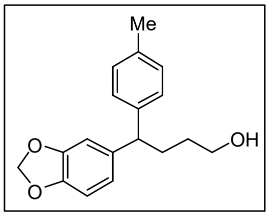

R_f = 0.30 (EtOAc:hexanes, 1.0:9.0); Yield = 0.26 g, (85%); ^{1}H-NMR (500 MHz, $CDCl_3$): δ = 7.12-7.07 (m, 4H, Ar*H*), 6.70 (s, *3*H, Ar*H*), 5.88-5.87 (m, 2H, OC*H*$_2$O), 3.78 (t, J = 8.0 Hz, 1H, C*H*), 3.63 (t, J = 6.5 Hz, 2H, C*H*$_2$), 2.29 (s, 3H, ArC*H*$_3$), 2.07-2.01 (m, 2H, C*H*$_2$), 1.59 (bs, 1H, O*H*), 1.52-1.49 (m, 2H, C*H*$_2$) ppm; ^{13}C-NMR (125 MHz, $CDCl_3$): δ = 147.8 (Ar*C*), 145.8 (Ar*C*), 142.1 (Ar*C*), 139.4 (Ar*C*), 135.8 (Ar*C*), 129.3 (Ar*C*H), 127.6 (Ar*C*H), 120.7 (Ar*C*H), 108.2 (Ar*C*H), 108.22 (Ar*C*H), 100.9 (O*C*H$_2$O), 63.0 (*C*H$_2$OH), 50.5 (*C*H), 32.0 (*C*H$_2$), 31.4 (*C*H$_2$), 21.0 (Ar*C*H$_3$) ppm; IR ($CHCl_3$) ν_{max} = 3400, 2936, 2883, 1503, 1487,

1438, 1118 cm^{-1}; HRMS (ESI): *m/z* calcd for $C_{18}H_{20}O_3$+Na^+: 307.1310 $[M+Na]^+$; found: 307.1288.

Compound 5b:

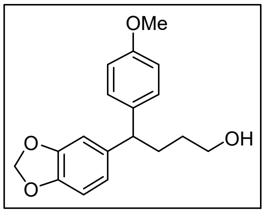

Colorless viscous liquid; R_f = 0.30 (EtOAc:hexanes, 1.0:9.0); Yield: 0.41 g, (85%); ^{1}H-NMR (400 MHz, $CDCl_3$): δ = 7.14-7.11 (m, 2H, Ar*H*), 6.83-6.80 (m, *2*H, Ar*H*), 6.70-6.67 (m, 3H, Ar*H*), 5.88-5.87 (m, 2H, OCH_2O), 3.79-3.75 (m, 4H, C*H* and ArOCH_3), 3.63 (t, *J* = 6.4 Hz, 2H, CH_2), 2.05-1.99 (m, 2H, CH_2), 1.65 (bs, 1H, OH), 1.53-1.48 (m, 2H, CH_2) ppm.; ^{13}C-NMR (100 MHz, $CDCl_3$): δ = 158.0 (Ar*C*), 147.8 (Ar*C*), 145.8 (Ar*C*), 139.5 (Ar*C*), 137.3 (Ar*C*), 128.6 (Ar*C*H), 120.7 (Ar*C*H), 113.9 (Ar*C*H), 108.24 (Ar*C*H), 108.20 (Ar*C*H), 100.9 (O*C*H_2O), 62.9 (O*C*H_2), 55.3 (ArO*C*H_3), 50.0 (*C*H), 32.2 (*C*H_2), 31.4 (*C*H_2) ppm; IR ($CHCl_3$) v_{max} = 3432, 2938, 2885, 1610, 1508, 1486, 1440 cm^{-1}; HRMS (ESI): *m/z* calcd for $C_{18}H_{20}O_4$+Na^+: 323.1259 $[M+Na]^+$; found: 323.1265.

Compound 5d:

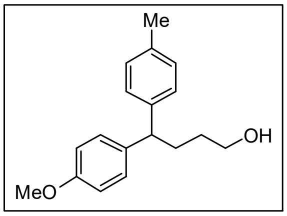

Colorless viscous liquid; R_f = 0.25 (EtOAc:hexanes, 1.0:9.0); Yield = 0.45 g, (90%); ^{1}H-NMR (500 MHz, $CDCl_3$): δ = 7.15-7.06 (m, 6H, Ar*H*), 6.82-6.79 (m, *2*H, Ar*H*), 3.81 (t, *J* = 7.5 Hz, 1H, C*H*), 3.75 (s, 3H, ArOCH_3), 3.63 (t, *J* = 6.5 Hz, 2H, CH_2), 2.28 (s, 3H, ArCH_3), 2.08-2.03 (m, 2H, CH_2), 1.64 (bs, 1H, O*H*), 1.54-1.49 (m, 2H, CH_2) ppm; ^{13}C-NMR (125 MHz, $CDCl_3$): δ = 157.9 (Ar*C*), 142.4 (Ar*C*), 137.4 (Ar*C*), 135.6 (ArC), 129.2 (Ar*C*H), 128.7 (Ar*C*H), 127.7 (Ar*C*H), 113.9 (Ar*C*H), 63.0 (*C*H_2OH), 55.3 (ArO*C*H_3), 49.9 (*C*H), 32.1 (*C*H_2), 31.4 (*C*H_2), 21.0 (Ar*C*H_3) ppm; IR ($CHCl_3$) v_{max} = 3421, 2937, 2870, 2839, 1609, 1583, 1510 cm^{-1}.

Compound 5e:

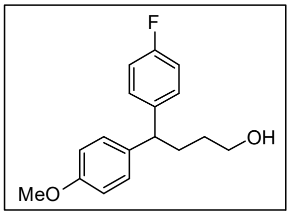

Colorless liquid; R_f = 0.25 (EtOAc:hexanes, 1.0:9.0); Yield = 0.55 g, (86%); ^{1}H-NMR (500 MHz, $CDCl_3$): δ = 7.17-7.11 (m, 4H, Ar*H*), 6.96-6.92 (m, *2*H, Ar*H*), 6.82-6.80 (m, 2H, Ar*H*), 3.83 (t, *J* = 7.5 Hz, 1H, C*H*), 3.76 (s, 3H, $ArOCH_3$), 3.63 (t, *J* = 6.5 Hz, 2H, CH_2), 2.07-2.03 (m, 2H, CH_2), 1.65 (bs, 1H, O*H*), 1.53-1.47 (m, 2H, CH_2) ppm; ^{13}C-NMR (125 MHz, $CDCl_3$): δ = 162.3 (d, *J* = 243.7 Hz, Ar*C*F), 158.1 (Ar*C*), 141.2 (d, *J* = 2.5 Hz, Ar*C*), 136.9 (Ar*C*), 129.2 (d, *J* = 7.5 Hz, Ar*C*H), 128.7 (Ar*C*H), 115.2 (d, *J* = 21.2 Hz, Ar*C*H), 114.0 (Ar*C*H), 62.9 (CH_2OH), 55.3 ($ArOCH_3$), 49.6 (*C*H), 32.2 (CH_2), 31.3 (CH_2) ppm; IR ($CHCl_3$) ν_{max} = 3428, 2938, 2876, 2839, 1606, 1509, 1461 cm^{-1}; HRMS (ESI): *m/z* calcd for $C_{17}H_{19}O_2F+Na^+$: 297.1267 $[M+Na]^+$; found: 297.1289.

Synthesis of compound 6: To a solution of **5a** (0.25 g, 0.88 mmol) in dry CH_2Cl_2 (5 mL) first, solid PPh_3 (0.46 g, 1.76 mmol) was added to reaction mixture followed by addition of CBr_4 (0.31 g, 1.76 mmol) under N_2 at 0 °C. Reaction mixture was stirred for 24 h at rt and monitored by TLC and after complete consumption of staring material, reaction solvent was evaporated. The crude compound was purified through silica gel column chromatography using 5% EtOAc in hexanes as eluent, which afforded compound **6a**.

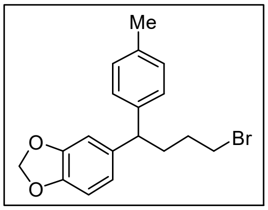

Colorless viscous liquid; R_f = 0.66 (EtOAc:hexanes, 1.0:9.0); Yield = 0.26 g, (86%); ^{1}H-NMR (500 MHz, $CDCl_3$): δ = 7.11-7.07 (m, 4H, Ar*H*), 6.72-6.68 (m, *3*H, Ar*H*), 5.87 (d, *J* = 2.5 Hz, 2H, OCH_2O), 3.79 (t, *J* = 7.5 Hz, 1H, C*H*), 3.39 (t, *J* = 6.5 Hz, 2H, CH_2), 2.29 (s, 3H, $ArCH_3$), 2.14-2.09 (m, 2H, CH_2), 1.84-1.78 (m, 2H, CH_2) ppm; ^{13}C-NMR (125 MHz, $CDCl_3$): δ = 147.8 (Ar*C*), 146.0 (Ar*C*), 141.6 (Ar*C*), 138.9 (Ar*C*), 136.0 (Ar*C*), 129.3 (Ar*C*H), 127.5 (Ar*C*H), 120.7 (Ar*C*H), 108.2 (Ar*C*H), 108.2 (Ar*C*H), 100.9 (OCH_2O), 49.9 (*C*H), 34.3 (CH_2), 33.9 (CH_2), 31.3 (CH_2), 21.1 ($ArCH_3$) ppm; IR ($CHCl_3$) ν_{max} = 1505, 1486, 1438, 1041, 932, 670 cm^{-1}; HRMS (ESI): *m/z* calcd for $C_{18}H_{19}O_2Br+K^+$: 385.0205 $[M+K]^+$; found: 385.0185.

Compound 6b:

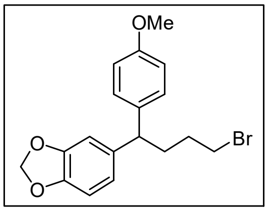

Colorless viscous liquid; R_f = 0.66 (EtOAc:hexanes, 1.0:9.0); Yield = 0.46 g, (89%); ^{1}H-NMR (400 MHz, $CDCl_3$): δ = 7.14-7.11 (m, 2H, Ar*H*), 6.84-6.80 (m, *2*H, Ar*H*), 6.72-6.67 (m, 3H, Ar*H*), 5.89-5.88 (m, 2H, OCH_2O), 3.79-3.76 (m, 4H, C*H* and $ArOCH_3$), 3.39 (t, *J* = 6.4 Hz, 2H, CH_2), 2.13-2.07 (m, 2H, CH_2), 1.84-1.87 (m, 2H, CH_2) ppm; ^{13}C-NMR (100 MHz, $CDCl_3$): δ = 158.1 (Ar*C*), 147.8 (Ar*C*), 145.9 (Ar*C*), 139.0 (Ar*C*), 136.8 (Ar*C*), 128.6 (Ar*C*H), 120.6 (Ar*C*H), 114.0 (Ar*C*H), 108.2 (Ar*C*H), 108.1 (Ar*C*H), 101.0 (OCH_2O), 55.3 ($ArOCH_3$), 49.5 (*C*H), 34.5 (CH_2), 33.9 (CH_2), 31.2 (CH_2) ppm; IR ($CHCl_3$) ν_{max} = 2937, 2897, 1610, 1507, 1487, 1440, 1246 cm^{-1}.

Compound 6c:

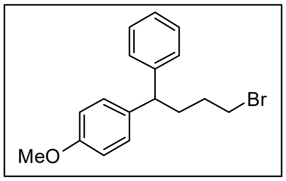

Colorless viscous liquid; R_f = 0.66 (EtOAc:hexanes, 1.0:9.0); Yield = 0.33 g, (80%); ^{1}H-NMR (500 MHz, $CDCl_3$): δ = 7.29-7.25 (m, 2H, Ar*H*), 7.22-7.20 (m, *2*H, Ar*H*), 7.18-7.13 (m, 3H, Ar*H*), 6.83-6.80 (m, 2H, Ar*H*), 3.86 (t, *J* = 8.0 Hz, 1H, C*H*), 3.76 (s, 3H, $ArOCH_3$), 3.40 (t, *J* = 6.5 Hz, 2H, CH_2), 2.18-2.14 (m, 2H, CH_2), 1.85-1.80 (m, 2H, CH_2) ppm; ^{13}C-NMR (125 MHz, $CDCl_3$): δ = 158.1 (Ar*C*), 145.0 (Ar*C*), 136.7 (Ar*C*), 128.8 (Ar*C*H), 128.6 (Ar*C*H), 127.8 (Ar*C*H), 126.3 (Ar*C*H), 114.0 (Ar*C*H), 55.3 ($ArOCH_3$), 49.6 (*C*H), 34.4 (CH_2), 33.9 (CH_2), 31.3 (CH_2) ppm; IR ($CHCl_3$) ν_{max} = 2956, 2937, 2911, 2837, 1609, 1510 cm^{-1}; HRMS (ESI): *m/z* calcd for $C_{17}H_{19}OBr+Na^+$: 341.0517 $[M+Na]^+$; found: 341.0513.

Compound 6d:

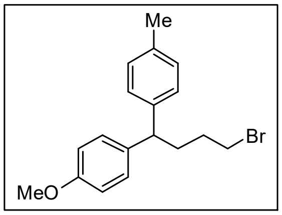

Colorless viscous liquid; R_f = 0.66 (EtOAc:hexanes, 1.0:9.0); Yield = 0.5 g, (92%); ^{1}H-NMR (400 MHz, $CDCl_3$): δ = 7.14-7.06 (m, 6H, Ar*H*), 6.83-6.79 (m, *2*H, Ar*H*), 3.82 (t, *J* = 8.0 Hz, 1H, C*H*), 3.76 (s, 3H, $ArOCH_3$), 3.39 (t, *J* = 6.4 Hz, 2H, CH_2), 2.29 (s, 3H, $ArCH_3$), 2.17-2.11 (m, 2H, CH_2), 1.84-1.77 (m, 2H, CH_2) ppm; ^{13}C-NMR (100 MHz, $CDCl_3$): δ = 158.1 (Ar*C*), 142.0 (Ar*C*), 137.0 (Ar*C*), 135.8 (Ar*C*H), 129.3 (Ar*C*H), 128.7 (Ar*C*H), 127.6 (Ar*C*H), 114.0 (Ar*C*H), 55.3 ($ArOCH_3$), 49.6 (*C*H), 34.4 (CH_2), 34.0 (CH_2), 31.3 (CH_2), 21.1 ($ArCH_3$) ppm; IR ($CHCl_3$) ν_{max} = 2935, 1645, 1609, 1510, 1459, 1440, 1300 cm^{-1}; HRMS (ESI): *m/z* calcd for $C_{18}H_{21}OBr+H^+$: 333.0854 $[M+H]^+$; found: 333.0867.

Compound 6e:

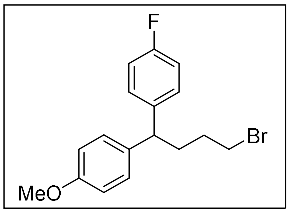

Colorless liquid; R_f = 0.65 (EtOAc:hexanes, 1.0:9.0); Yield = 0.45 g, (76%); ^{1}H-NMR (500 MHz, $CDCl_3$): δ = 7.17-7.15 (m, 2H, Ar*H*), 7.13-7.10 (m, 2H, Ar*H*), 6.98-6.93 (m, *2*H, Ar*H*), 6.84-6.81 (m, 2H, Ar*H*), 3.84 (t, *J* = 7.7 Hz, 1H, C*H*), 3.76 (s, 3H, ArOCH_3), 3.39 (t, *J* = 6.6 Hz, 2H, CH_2), 2.17-2.11 (m, 2H, CH_2), 1.83-1.72 (m, 2H, CH_2) ppm; ^{13}C-NMR (125 MHz, $CDCl_3$): δ = 161.4 (d, *J* = 243.7 Hz, Ar*C*F), 158.2 (Ar*C*), 140.7 (d, *J* = 3.2 Hz, Ar*C*), 136.4 (Ar*C*), 129.2 (d, *J* = 7.7 Hz, Ar*C*H), 115.3 (d, *J* = 20.9 Hz, Ar*C*H), 114.1 (Ar*C*H), 55.3 (ArO*C*H_3), 49.1 (*C*H), 34.5 (*C*H_2), 33.8 (*C*H_2), 31.2 (*C*H_2) ppm. HRMS (ESI): *m/z* calcd for $C_{17}H_{18}OBrF+Na^+$: 359.0423 $[M+Na]^+$; found: 359.0401

Synthesis of compound 7 and 8: The solution of compound **6a** (111 mg, 0.32 mmol) in dry acetone (5 mL) was refluxed with morpholine (0.80 mmol) and K_2CO_3 (106 mg, 0.80 mmol) for 20-22 h under N_2. After complete consumption of staring material, solvent was evaporated and crude compound was purified through silica gel column chromatography using 60-80% EtOAc in hexanes as eluent, which afforded compound **7a** as viscous liquid.

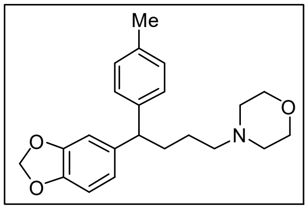

Colorless viscous liquid; R_f) = 0.25 (Acetone); Yield = 100 mg, (88%); ^{1}H-NMR (400 MHz, $CDCl_3$): δ = 7.11-7.06 (m, 4H, Ar*H*), 6.71-6.67 (m, 3H, Ar*H*), 5.88-5.87 (m, 2H, OCH_2O), 3.77 (t, *J* = 7.6 Hz, 1H, C*H*), 3.67 (t, *J* = 4.8 Hz, 4H, 2 x CH_2), 2.37-2.31 (m, 6H, 3 x CH_2), 2.28 (s, 3H, ArCH_3), 2.01-1.95 (m, 2H, CH_2), 1.47-1.41 (m, 2H, CH_2) ppm; ^{13}C-NMR (100 MHz, $CDCl_3$): δ = 147.8 (Ar*C*), 145.8 (Ar*C*), 142.2 (Ar*C*), 139.4 (Ar*C*), 135.7 (Ar*C*), 129.2 (Ar*C*H), 127.6 (Ar*C*H), 120.7 (Ar*C*H), 108.26 (Ar*C*H), 108.20 (Ar*C*H), 100.9 (O*C*H_2O), 67.1 (*C*H_2), 59.0 (*C*H_2), 53.8 (*C*H_2), 50.6 (*C*H), 33.7 (*C*H_2), 25.1 (*C*H_2), 21.0 (Ar*C*H_3) ppm; IR ($CHCl_3$) ν_{max} = 2940, 2895, 2866, 2812, 1504, 1486, 1441 cm^{-1}; HRMS (ESI): *m/z* calcd for $C_{22}H_{27}NO_3+H^+$: 354.2069 $[M+H]^+$; found: 354.2099.

Compound 7b:

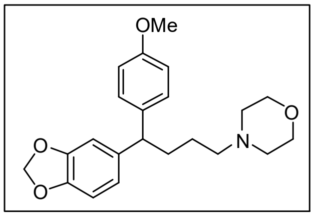

Yellow viscous liquid; R_f = 0.25 (Acetone); Yield = 129 mg, (93%); ^{1}H-NMR (500 MHz, $CDCl_3$): δ = 7.13-7.11 (m, 2H, Ar*H*), 6.82-6.79 (m, 2H, Ar*H*), 6.71-6.66 (m, 3H, Ar*H*), 5.89-5.88 (m, 2H, OCH_2O), 3.78-3.74 (m, 4H, C*H* and $ArOCH_3$), 3.68 (t, *J* = 4.5 Hz, 4H, 2 x CH_2), 2.37-2.31 (m, 6H, 3 x CH_2), 1.99-1.94 (m, 2H, CH_2), 1.46-1.41 (m, 2H, CH_2) ppm; ^{13}C-NMR (125 MHz, $CDCl_3$): δ = 158.0 (Ar*C*), 147.7 (Ar*C*), 145.8 (Ar*C*), 139.6 (Ar*C*), 137.3 (Ar*C*), 128.6 (Ar*C*H), 120.7 (Ar*C*H), 113.9 (Ar*C*H), 108.2 (Ar*C*H), 100.9 (OCH_2O), 67.1 (CH_2), 59.0 (CH_2), 55.3 ($ArOCH_3$), 53.8 (CH_2), 50.2 (*C*H), 33.8 (CH_2), 25.1 (CH_2) ppm; IR ($CHCl_3$) ν_{max} = 2942, 1610, 1508, 1486, 1441, 1300, 1244 cm^{-1}; HRMS (ESI): *m/z* calcd for $C_{22}H_{27}NO_4+H^+$: 370.2018 $[M+H]^+$; found: 370.2045.

Compound 7c:

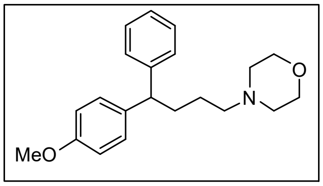

Colorless viscous liquid; R_f = 0.34 (Acetone); Yield = 96 mg, (76%); ^{1}H-NMR (500 MHz, $CDCl_3$): δ = 7.27-7.24 (m, 2H, Ar*H*), 7.22-7.20 (m, 2H, Ar*H*), 7.16-7.12 (m, 3H, Ar*H*), 6.82-6.79 (m, 2H, Ar*H*), 3.84 (t, *J* = 8.0 Hz, 1H, C*H*), 3.76 (s, 3H, $ArOCH_3$), 3.67 (t, *J* = 4.5 Hz, 4H, 2 x CH_2), 2.36-2.32 (m, 6H, 3 x CH_2), 2.05-2.00 (m, 2H, CH_2), 1.47-1.41 (m, 2H, CH_2) ppm; ^{13}C-NMR (125 MHz, $CDCl_3$): δ = 158.0 (Ar*C*), 145.5 (Ar*C*), 137.2 (Ar*C*), 128.8 (Ar*C*H), 128.5 (Ar*C*H), 127.8 (Ar*C*H), 126.1 (Ar*C*H), 113.9 (Ar*C*H), 67.1 (CH_2), 59.0 (CH_2), 55.3 ($ArOCH_3$), 50.5 (*C*H), 33.8 (CH_2), 25.1 (CH_2) ppm; IR ($CHCl_3$) ν_{max} = 2940, 1609, 1511, 1457, 1246, 1181, 1116 cm^{-1}; HRMS (ESI): *m/z* calcd for $C_{21}H_{27}NO_2+H^+$: 326.2120 $[M+H]^+$; found: 326.2142.

Compound 7d:

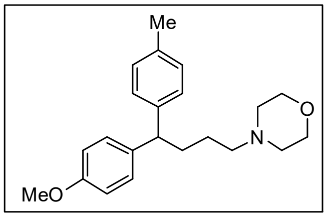

Colorless viscous liquid; R_f = 0.30 (Acetone); Yield = 140 mg, (92%); ^{1}H-NMR (500 MHz, $CDCl_3$): δ = 7.14-7.05 (m, 6H, Ar*H*), 6.81-6.78 (m, 2H, Ar*H*), 3.80 (t, *J* = 7.5 Hz, 1H, C*H*), 3.75 (s, 3H, ArOCH_3), 3.67 (t, *J* = 4.5 Hz, 4H, 2 x CH_2), 2.36-2.32 (m, 6H, 3 x CH_2), 2.28 (s, 3H, ArCH_3), 2.02-1.98 (m, 2H, CH_2), 1.47-1.42 (m, 2H, CH_2) ppm; ^{13}C-NMR (125 MHz, $CDCl_3$): δ = 157.9 (Ar*C*), 142.5 (Ar*C*), 137.5 (Ar*C*), 135.6 (Ar*C*), 129.2 (Ar*C*H), 128.7 (Ar*C*H), 127.6 (Ar*C*H), 113.9 (Ar*C*H), 67.1 (*C*H_2), 59.1 (*C*H_2), 55.3 (ArO*C*H_3), 53.8 (*C*H_2), 50.1 (*C*H), 33.8 (*C*H_2), 25.2 (*C*H_2), 21.0 (Ar*C*H_3) ppm; IR ($CHCl_3$) ν_{max} = 2937, 2864, 1653, 1610, 1510, 1459, 1247 cm^{-1}; HRMS (ESI): *m/z* calcd for $C_{22}H_{29}NO_2+H^+$: 340.2277 $[M+H]^+$; found: 340.2246.

Compound 7e:

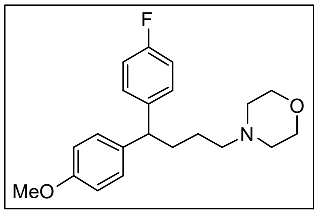

Colorless viscous liquid; R_f = 0.30 (Acetone); Yield = 129 mg, (72%); ^{1}H-NMR (500 MHz, $CDCl_3$): δ = 7.17-7.14 (m, 2H, Ar*H*), 7.11 (d, *J* = 8.5 Hz, 2H, Ar*H*), 6.96-6.92 (m, 2H, Ar*H*), 6.81 (d, *J* = 9Hz, 2H, Ar*H*), 3.82 (t, *J* = 8.0 Hz, 1H, C*H*), 3.76 (s, 3H, ArOCH_3), 3.67 (t, *J* = 4.5 Hz, 4H, 2 x CH_2), 2.36-2.31 (m, 6H, 3 x CH_2), 2.02-1.97 (m, 2H, CH_2), 1.45-1.39 (m, 2H, CH_2) ppm; ^{13}C-NMR (125 MHz, $CDCl_3$): δ = 161.3 (d, *J* = 242.5 Hz, Ar*C*F), 158.1 (Ar*C*), 141.2 (d, *J* = 2.5 Hz, Ar*C*), 137.0 (Ar*C*), 129.1 (d, *J* = 7.5 Hz, Ar*C*H), 128.7 (Ar*C*H), 115.2 (d, *J* = 21.2 Hz, Ar*C*H), 114.0 (Ar*C*H), 67.1 (*C*H_2O), 59.0 (*C*H_2), 55.3 (ArO*C*H_3), 53.8 (*C*H_2), 49.8 (*C*H), 33.9 (*C*H_2), 25.1 (*C*H_2) ppm; IR ($CHCl_3$) ν_{max} = 2942, 2863, 2814, 1606, 1509, 1460, 1245 cm^{-1}; HRMS (ESI): *m/z* calcd for $C_{21}H_{26}NO_2F+H^+$: 344.2026 $[M+H]^+$; found: 344.2036.

Compound 8a:

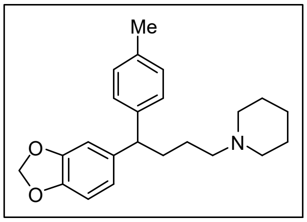

Yellow viscous liquid; R_f = 0.25 (Acetone); Yield = 114 mg, (93%); ^{1}H-NMR (400 MHz, $CDCl_3$): δ = 7.11-7.05 (m, 4H, Ar*H*), 6.71-6.66 (m, 3H, Ar*H*), 5.88-5.87 (m, 2H, OCH_2O), 3.76 (t, *J* = 8.0 Hz, 1H, C*H*), 2.30-2.26 (m, 9H, 3 x CH_2 and ArCH_3), 1.98-1.92 (m, 2H, CH_2), 1.57-1.51 (m, 4H, 2 x CH_2), 1.48-1.40 (m, 4H, 2 x CH_2) ppm; ^{13}C-NMR (100 MHz, $CDCl_3$):

δ = 147.7 (Ar*C*), 145.8 (Ar*C*), 142.3 (Ar*C*), 139.5 (Ar*C*), 135.6 (Ar*C*), 129.2 (Ar*C*H), 127.6 (Ar*C*H), 120.8 (Ar*C*H), 108.3 (Ar*C*H), 108.1 (Ar*C*H), 100.9 (O*C*H_2O), 59.5 (*C*H_2), 54.7 (*C*H_2), 50.7 (*C*H), 34.0 (*C*H_2), 26.0 (*C*H_2), 25.6 (*C*H_2), 24.6 (*C*H_2), 21.1 (Ar*C*H_3) ppm; IR ($CHCl_3$) ν_{max} = 2936, 2861, 1505, 1485, 1440, 1041, 931 cm^{-1}; HRMS (ESI): *m/z* calcd for $C_{23}H_{29}NO_2$+H^+: 352.2277 $[M+H]^+$; found: 352.2265.

Compound 8b:

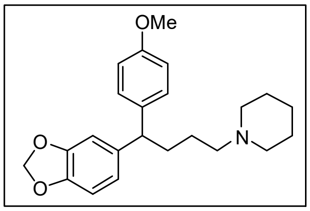

Yellow viscous liquid; R_f = 0.25 (Acetone); Yield = 130 mg, (91%); ^{1}H-NMR (400 MHz, $CDCl_3$): δ = 7.13-7.10 (m, 2H, Ar*H*), 6.82-6.78 (m, 2H, Ar*H*), 6.69-6.66 (m, 2H, Ar*H*), 5.88-5.87 (m, 2H, OC*H_2*O), 3.77-3.73 (m, 4H, C*H* and ArC*H_3*), 2.31-2.27 (m, 6H, 3 x C*H_2*), 1.96-1.89 (m, 2H, C*H_2*), 1.57-1.51 (m, 4H, 2 x C*H_2*), 1.47-1.41 (m, 4H, 2 x C*H_2*) ppm; ^{13}C-NMR (100 MHz, $CDCl_3$): δ = 158.0 (Ar*C*), 147.7 (Ar*C*), 145.8 (Ar*C*), 139.7 (Ar*C*), 137.5 (Ar*C*), 128.6 (Ar*C*H), 120.7 (Ar*C*H), 113.9 (Ar*C*H), 108.2 (Ar*C*H), 108.1 (Ar*C*H), 100.9 (O*C*H_2O), 59.5 (*C*H_2), 55.3 (ArO*C*H_3), 54.7 (CH2), 50.2 (*C*H), 34.1 (*C*H_2), 26.0 (*C*H_2), 25.6 (*C*H_2), 24.6 (*C*H_2) ppm; IR ($CHCl_3$) ν_{max} = 2938, 2861, 2806, 1609, 1508, 1486, 1441 cm^{-1}; HRMS (ESI): *m/z* calcd for $C_{23}H_{29}NO_3$+H^+: 368.2226 $[M+H]^+$; found: 368.2206.

Compound 8c:

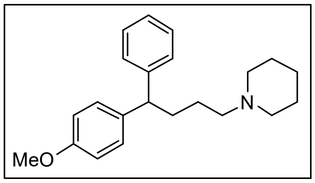

Yellow viscous liquid; R_f = 0.43 (Acetone); Yield = 125 mg, (77%); ^{1}H-NMR (500 MHz, $CDCl_3$): δ = 7.27-7.24 (m, 2H, Ar*H*), 7.21-7.19 (m, 2H, Ar*H*), 7.16-7.12 (m, 3H, Ar*H*), 6.82-6.79 (m, 2H, Ar*H*), 3.84 (t, *J* = 7.5 Hz, 1H, C*H*), 3.76 (s, 3H, ArOC*H_3*), 2.31-2.28 (m, 5H), 2.01-1.95 (m, 3H), 1.56-1.52 (m, 4H, 2 x C*H_2*), 1.48-1.43 (m, 2 x C*H_2*) ppm; ^{13}C-NMR (125 MHz, $CDCl_3$): δ = 157.9 (Ar*C*), 145.6 (Ar*C*), 137.4 (Ar*C*), 128.8 (Ar*C*H), 128.5 (Ar*C*H), 127.8 (Ar*C*H), 126.0 (Ar*C*H), 113.8 (Ar*C*H), 59.5 (*C*H_2), 55.3 (ArO*C*H_3), 54.7 (*C*H_2), 50.6 (*C*H), 34.0 (*C*H_2), 26.0 (*C*H_2), 25.5 (C*H_2*), 24.5 (C*H_2*) ppm; IR ($CHCl_3$) ν_{max} = 2938, 1511, 1245, 669 cm^{-1}; HRMS (ESI): *m/z* calcd for $C_{22}H_{29}NO$+H^+: 324.2327 $[M+H]^+$; found: 324.2341.

Compound 8d:

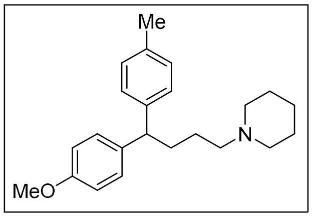

Yellow viscous liquid; R_f = 0.40 (Acetone); Yield = 120 mg, (75%); ^{1}H-NMR (500 MHz, $CDCl_3$): δ = 7.13-7.04 (m, 6H, Ar*H*), 6.80-6.78 (m, 2H, Ar*H*), 3.80 (t, J = 7.8 Hz, 1H, C*H*), 3.75 (s, 3H, ArOCH_3), 2.30-2.28 (m, 8H), 1.99-1.94 (m, 3H), 1.56-1.52 (m, 4H, 2 x CH_2), 1.48-1.43 (m, 2 x CH_2) ppm; ^{13}C-NMR (125 MHz, $CDCl_3$): δ = 157.9 (Ar*C*), 142.6 (Ar*C*), 137.6 (Ar*C*), 135.5 (Ar*C*), 129.1 (Ar*C*H), 128.7 (Ar*C*H), 127.7 (Ar*C*H), 113.8 (Ar*C*H), 59.5 (*C*H_2), 55.3 (ArO*C*H_3), 54.7 (*C*H_2), 50.1 (*C*H), 34.1 (*C*H_2), 26.0 (*C*H_2), 25.6 (CH_2), 24.6 (CH_2), 21.0 (Ar*C*H_3) ppm. HRMS (ESI): *m/z* calcd for $C_{23}H_{31}NO+K^+$: 376.2043 $[M+K]^+$; found: 376.2042.

Compound 8e:

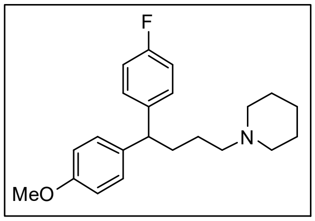

Yellow viscous liquid; R_f = 0.33 (Acetone); Yield = 159 mg, (92%); ^{1}H-NMR (500 MHz, $CDCl_3$): δ = 7.16-7.14 (m, 2H, Ar*H*), 7.12-7.09 (m, 2H, Ar*H*), 6.96-6.92 (m, 2H, Ar*H*), 6.82-6.79 (m, 2H, Ar*H*), 3.82 (t, J = 8.0 Hz, 1H, C*H*), 3.76 (s, 3H, ArOCH_3), 2.30-2.27 (m, 6H, 3 x CH_2), 1.99-1.94 (m, 2H, CH_2), 1.56-1.52 (m, 4H, 2 x CH_2), 1.46-1.41 (m, 2 x CH_2) ppm; ^{13}C-NMR (125 MHz, $CDCl_3$): δ = 161.3 (d, J = 242.5 Hz, Ar*C*F), 158.0 (Ar*C*), 141.3 (Ar*C*), 137.2 (Ar*C*), 129.2 (d, J = 7.5 Hz, Ar*C*H), 128.7 (Ar*C*H), 115.2 (d, J = 21.2 Hz, Ar*C*H), 113.9 (Ar*C*H), 59.5 (*C*H_2), 55.3 (ArO*C*H_3), 54.7 (*C*H_2), 49.8 (*C*H), 34.2 (*C*H_2), 26.0 (*C*H_2), 25.5 (CH_2), 24.5 (CH_2) ppm IR ($CHCl_3$) ν_{max} = 2938, 1607, 1509, 1035, 668, 625 cm^{-1}.

Synthesis of compound 1 and 2: To a solution of compound **7a** (0.3 mmol) in dry CH_2Cl_2, 0.09 mL of 4M HCl in 1,4-dioxane (0.36 mmol) solution was added and reaction mixture was stirred at rt for 24 h. Now, solvent was evaporated and dried under high vacuum and then product was washed with dry diethyl ether and drying under high vacuum furnished compound **1a**.

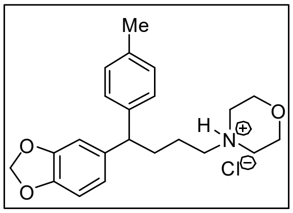

Colorless solid; Yield = 96 mg, (83%); ^{1}H-NMR (500 MHz, DMSO-d_6): δ = 10.72 (bs, 1H, N*H*), 7.17 (d, *J* = 8.0 Hz, 2H, Ar*H*), 7.08 (d, *J* = 8.0 Hz, 2H, Ar*H*), 6.87 (d, *J* = 1.5 Hz, 1H, Ar*H*), 6.80 (d, *J* = 8.0 Hz, 1H, Ar*H*), 6.74 (dd, *J* = 8.0, 1.5 Hz, 1H, Ar*H*), 5.94-5.93 (m, 2H, OCH_2O), 3.94-3.85 (broad signal, 2H, CH_2), 3.83-3.72 (m, 3H), 3.32-3.24 (broad signal, 2H, CH_2), 3.12-3.07 (broad signal, 2H, CH_2), 3.01-2.89 (broad signal, 2H, CH_2), 2.00-1.96 (m, 2H, CH_2), 1.63-1.52 (broad signal, 2H, CH_2) ppm; ^{13}C-NMR (125 MHz, DMSO-d_6): δ = 147.2 (Ar*C*), 145.3 (Ar*C*), 141.8 (Ar*C*), 138.9 (Ar*C*), 135.0 (Ar*C*), 128.9 (Ar*C*H), 127.2 (Ar*C*H), 120.4 (Ar*C*H), 108.0 (Ar*C*H), 107.7 (Ar*C*H), 100.7 (O*C*H$_2$O), 63.0 (*C*H$_2$), 55.7 (*C*H$_2$), 50.9 (*C*H$_2$), 49.2 (*C*H), 31.7 (*C*H$_2$), 21.5 (*C*H$_2$), 20.5 (Ar*C*H$_3$) ppm; IR (CHCl$_3$) ν_{max} = 3434, 1637, 1546, 1510, 1485, 1436, 928 cm^{-1}; HRMS (ESI): *m/z* calcd for $C_{22}H_{28}NO_3$-Cl^+: 354.2069 [M-Cl]$^+$; found: 354.2074.

Compound 1b:

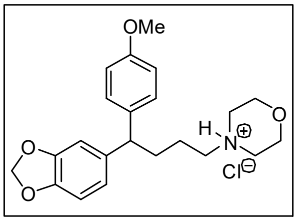

Colorless solid; Yield = 122 mg, (93%); ^{1}H-NMR (500 MHz, DMSO-d_6): δ = 10.77 (bs, 1H, N*H*), 7.24 (d, *J* = 8.5 Hz, 2H, Ar*H*), 6.90-6.82 (m, 4H, Ar*H*), 6.78 (d, *J* = 8.0 Hz, 1H, Ar*H*), 5.97 (d, *J* = 4.0 Hz, 2H, OCH_2O), 3.97-3.91 (broad signal, 2H, CH_2), 3.86-3.75 (m, 3H), 3.73 (s, 3H, ArOCH_3), 3.35-3.28 (broad signal, 2H, CH_2), 3.16-3.06 (broad signal, 2H, CH_2), 3.05-2.93 (broad signal, 2H, CH_2), 2.07-1.95 (broad signal, 2H, CH_2), 1.66-1.55 (broad signal, 2H, CH_2) ppm; ^{13}C-NMR (125 MHz, DMSO-d_6): δ = 157.5 (Ar*C*), 147.3 (Ar*C*), 145.3 (Ar*C*), 139.1 (Ar*C*), 136.8 (Ar*C*), 128.3 (Ar*C*H), 120.4 (Ar*C*H), 113.7 (Ar*C*H), 108.0 (Ar*C*H), 107.7 (Ar*C*H), 100.7 (O*C*H$_2$O), 63.1 (*C*H$_2$), 55.7 (*C*H$_2$), 54.9 (ArO*C*H$_3$), 50.9 (*C*H$_2$), 48.8 (*C*H), 31.9 (*C*H$_2$), 21.5 (*C*H$_2$) ppm; IR (CHCl$_3$) ν_{max} = 3435, 2959, 1610, 1508, 1486, 1441, 1301 cm^{-1}; HRMS (ESI): *m/z* calcd for $C_{22}H_{28}NO_4$-Cl^+: 370.2018 [M-Cl]$^+$; found: 370.2021.

Compound 1c:

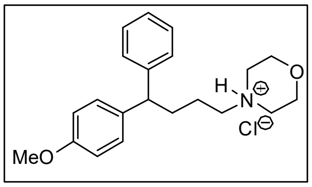

Colorless solid; Yield = 90 mg, (94%); ^{1}H-NMR (500 MHz, DMSO-d_6): δ = 10.76 (bs, 1H, N*H*), 7.30-7.27 (m, 4H, Ar*H*), 7.23 (d, *J* = 8.5 Hz, 2H, Ar*H*), 7.19-7.15 (m, 1H, Ar*H*), 6.86

(d, J = 8.5 Hz, 2H, ArH), 3.90 (t, J = 8.0 Hz, 1H, CH), 3.76 (t, J = 12.0 Hz, 2H, CH_2), 3.71 (s, 3H, ArOCH_3), 3.33-3.27 (broad signal, 2H, CH_2), 3.16-3.06 (broad signal, 2H, CH_2), 3.04-2.90 (broad signal, 2H, CH_2), 2.06-2.01 (m, 2H, CH_2), 1.63-1.60 (m, 2H, CH_2) ppm; ^{13}C-NMR (125 MHz, DMSO-d_6): δ = 157.5 (ArC), 145.0 (ArC), 136.5 (ArC), 128.4 (ArCH), 128.3 (ArCH), 127.4 (ArCH), 125.9 (ArCH), 113.8 (ArCH), 63.0 (CH$_2$), 55.7 (CH$_2$), 54.9 (ArOCH$_3$), 50.9 (CH$_2$), 49.1 (CH), 31.8 (CH$_2$), 21.5 (CH$_2$) ppm; IR (CHCl$_3$) ν_{max} = 3446, 2962, 1610, 1511, 1448, 1182, 1116 cm^{-1}; HRMS (ESI): m/z calcd for $[C_{21}H_{27}NO_2+H]^+$: 326.2120; found: 326.2146.

Compound 1d:

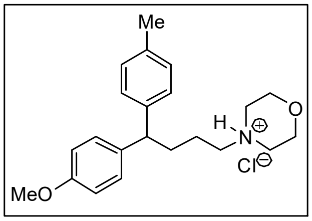

Colorless solid; Yield = 142 mg, (91%); ^{1}H-NMR (500 MHz, DMSO-d_6): δ = 10.04 (bs, 1H, NH), 7.21-7.16 (m, 4H, ArH), 7.09 (d, J = 8.0 Hz, 2H, ArH), 6.86-6.83 (m, 2H, ArH), 3.91-3.77 (m, 5H), 3.71 (s, 3H, ArOCH_3), 3.32-3.24 (broad signal, 2H, CH_2), 3.14-3.04 (broad signal, 2H, CH_2), 3.02-2.90 (broad signal, 2H, CH_2), 2.25 (s, 3H, ArCH_3), 2.03-1.98 (m, 2H, CH_2), 1.63-1.57 (m, 2H, CH_2) ppm; ^{13}C-NMR (125 MHz, DMSO-d_6): δ = 157.4 (ArC), 141.9 (ArC), 136.8 (ArC), 134.9 (ArC), 128.9 (ArCH), 128.3 (ArCH), 127.2 (ArCH), 113.7 (ArCH), 63.0 (CH$_2$), 55.7 (CH$_2$), 54.9 (ArOCH$_3$), 50.8 (CH$_2$), 48.7 (CH), 31.9 (CH$_2$), 21.5 (CH$_2$), 20.4 (ArCH$_3$) ppm; IR (CHCl$_3$) ν_{max} = 3442, 1609, 1512, 1426, 1033, 928, 628 cm^{-1}; HRMS (ESI): m/z calcd for $C_{22}H_{30}NO_2$-Cl^+: 340.2277 [M-Cl]$^+$; found: 340.2265.

Compound 1e:

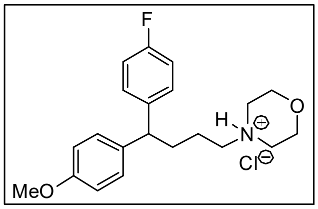

Colorless solid; Yield = 106 mg, (80%); ^{1}H-NMR (500 MHz, DMSO-d_6): δ = 10.91 (bs, 1H, NH), 7.34-7.30 (m, 2H, ArH), 7.24-7.22 (m, 2H, ArH), 7.13-7.09 (m, 2H, ArH), 6.86 (d, J = 8.5 Hz, 2H, ArH), 3.94-3.89 (m, 3H), 3.78 (t, J = 12.0 Hz, 2H, CH_2), 3.71 (s, 3H, ArOCH_3), 3.32-3.25 (broad signal, 2H, CH_2), 3.04-2.97 (broad signal, 2H, CH_2), 2.81-2.70 (broad signal, 2H, CH_2), 2.04-2.00 (m, 2H, CH_2), 1.62-1.56 (m, 2H, CH_2) ppm; ^{13}C-NMR (125 MHz, DMSO-d_6): δ = 160.5 (d, J = 241.2 Hz, ArCF), 157.6 (ArC), 141.2 (ArC), 136.3 (ArC),

129.1 (d, J = 7.5 Hz, Ar*C*H), 128.4 (Ar*C*H), 115.0 (d, J = 21.2 Hz, Ar*C*H), 113.8 (Ar*C*H), 63.0 (*C*H_2), 55.7 (*C*H_2), 54.9 (ArO*C*H_3), 50.8 (*C*H_2), 48.3 (*C*H), 32.0 (*C*H_2), 21.4 (*C*H_2) ppm; IR ($CHCl_3$) ν_{max} = 3451, 2963, 1609, 1509, 1437, 927, 669 cm^{-1}; HRMS (ESI): *m/z* calcd for $[C_{21}H_{26}FNO_2+H]^+$: 344.2025; found: 344.2020.

Compound 2a:

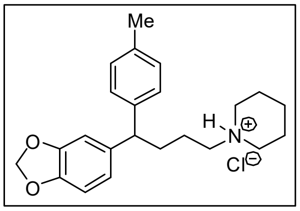

Colorless solid; Yield = 109 mg, (90%); ^{1}H-NMR (500 MHz, DMSO-d_6): δ = 10.10 (bs, 1H, N*H*), 7.19 (d, J = 8.5 Hz, 2H, Ar*H*), 7.09 (d, J = 8.0 Hz, 2H, Ar*H*), 6.88 (d, J = 1.5 Hz, 1H, Ar*H*), 6.81 (d, J = 8.0 Hz, 1H, Ar*H*), 6.76 (dd, J = 8.0, 1.5 Hz, 1H, Ar*H*), 5.96-5.95 (m, 2H, OC*H*$_2$O), 3.84 (t, J = 8.0 Hz, 1H, C*H*), 3.31-3.28 (broad signal, 2H, C*H*$_2$), 3.02-2.98 (m, 2H, C*H*$_2$), 2.80-2.72 (m, 2H, C*H*$_2$), 2.01-1.96 (m, 2H, C*H*$_2$), 1.74-1.65 (m, 5H, C*H*$_2$), 1.61-1.55 (m, 2H, C*H*$_2$), 1.39-1.30 (m, 1H) ppm; ^{13}C-NMR (125 MHz, DMSO-d_6): δ = 147.2 (Ar*C*), 145.3 (Ar*C*), 141.8 (Ar*C*), 138.9 (Ar*C*), 135.0 (Ar*C*), 128.9 (Ar*C*H), 127.2 (Ar*C*H), 120.4 (Ar*C*H), 108.0 (Ar*C*H), 107.7 (Ar*C*H), 100.6 (O*C*H_2O), 55.6 (*C*H_2), 51.8 (*C*H_2), 49.2 (*C*H), 31.8 (*C*H_2), 22.2 (*C*H_2), 21.8 (*C*H_2), 21.3 (*C*H_2), 20.5 (Ar*C*H_3) ppm; IR ($CHCl_3$) ν_{max} = 3434, 2957, 1508, 1486, 1439, 1038, 929 cm^{-1};

Compound 2b:

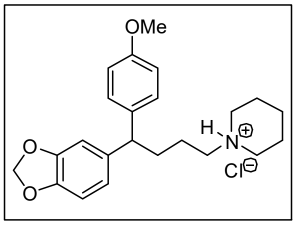

Colorless solid; Yield = 124 mg, (91%); ^{1}H-NMR (500 MHz, DMSO-d_6): δ = 10.26 (bs, 1H, N*H*), 7.36 (d, J = 8.5 Hz, 2H, Ar*H*), 7.02-6.99 (m, 3H, Ar*H*), 6.96 (d, J = 8.0 Hz, 1H, Ar*H*), 6.90 (dd, J = 8.0, 1.5 Hz, 1H, Ar*H*), 6.10 (d, J = 4.0, 1.5 Hz, 2H, OC*H*$_2$O), 3.97 (t, J = 8.0 Hz, 1H, C*H*), 3.86 (s, 3H, ArOC*H*$_3$), 3.45-3.43 (broad signal, 2H, C*H*$_2$), 3.17-3.12 (m, 2H, C*H*$_2$), 2.94-2.89 (m, 2H, C*H*$_2$), 2.14-2.10 (m, 2H, C*H*$_2$), 1.94-1.69 (m, 7H), 1.53-1.48 (m, 1H) ppm. ^{13}C-NMR (125 MHz, DMSO-d_6): δ = 157.5 (Ar*C*), 147.2 (Ar*C*), 145.3 (Ar*C*), 139.1 (Ar*C*), 136.8 (Ar*C*), 128.3 (Ar*C*H), 120.3 (Ar*C*H), 113.7 (Ar*C*H), 108.0 (Ar*C*H), 107.7 (Ar*C*H), 100.6 (O*C*H_2O), 55.6 (*C*H_2), 54.9 (*C*H_2), 51.8 (*C*H_2), 48.7 (*C*H), 31.9 (*C*H_2), 22.1 (*C*H_2), 21.7 (*C*H_2), 21.3 (*C*H_2) ppm; IR ($CHCl_3$) ν_{max} = 3465, 1639, 1512, 929, 760, 624, 580, 557 cm^{-1}; HRMS (ESI): *m/z* calcd for $[C_{23}H_{29}NO_3 + H]^+$: 368.2225; found: 368.2217.

Compound 2c:

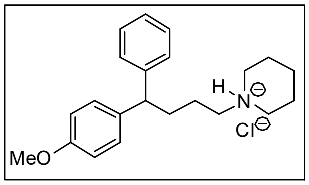

Colorless solid; Yield = 118 mg, (88%); ^{1}H-NMR (500 MHz, DMSO-d_6): δ = 10.13 (bs, 1H, N*H*), 7.31-7.27 (m, 4H, Ar*H*), 7.23 (d, *J* = 9.0 Hz, 2H, Ar*H*), 7.19-7.15 (m, 1H, Ar*H*), 6.86 (d, *J* = 8.5 Hz, 2H, Ar*H*), 3.90 (t, *J* = 8.0 Hz, 1H, C*H*), 3.71 (s, 3H, ArOCH_3), 3.30-3.28 (broad signal, 2H, CH_2), 3.03-2.99 (m, 2H, CH_2), 2.79-2.72 (m, 2H, CH_2), 2.05-2.00 (m, 2H, CH_2), 1.79-1.56 (m, 7H), 1.40-1.30 (m, 1H) ppm; ^{13}C-NMR (125 MHz, DMSO-d_6): δ = 157.5 (Ar*C*), 145.1 (Ar*C*), 136.5 (Ar*C*), 128.4 (Ar*C*H), 128.3 (Ar*C*H), 127.4 (Ar*C*H), 125.9 (Ar*C*H), 113.7 (Ar*C*H), 55.6 (CH_2), 54.9 (ArOCH_3), 51.8 (CH_2), 49.1 (*C*H), 31.9 (CH_2), 22.1 (CH_2), 21.8 (CH_2), 21.3 (CH_2) ppm; IR ($CHCl_3$) ν_{max} = 3437, 2954, 1610, 1511, 1452, 1427, 1033 cm^{-1}; HRMS (ESI): *m/z* calcd for $[C_{22}H_{29}NO+H]^+$: 324.2327; found: 324.2326.

Compound 2d:

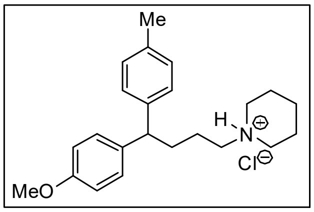

Colorless solid; Yield = 130 mg, (87%); ^{1}H-NMR (500 MHz, DMSO-d_6): δ = 10.21 (bs, 1H, N*H*), 7.22-7.16 (m, 4H, Ar*H*), 7.09 (d, *J* = 7.5 Hz, 2H, Ar*H*), 6.86-6.83 (m, 2H, Ar*H*), 3.85 (t, *J* = 8.0 Hz, 1H, C*H*), 3.71 (s, 3H, ArOCH_3), 3.30-3.25 (broad signal, 2H, CH_2), 3.02-2.98 (m, 2H, CH_2), 2.79-2.72 (m, 2H, CH_2), 2.25 (s, 3H, ArCH_3), 2.02-1.97 (m, 2H, CH_2), 1.77-1.55 (m, 7H), 1.39-1.32 (m, 1H) ppm; ^{13}C-NMR (125 MHz, DMSO-d_6): δ = 157.4 (Ar*C*), 142.0 (Ar*C*), 136.8 (Ar*C*), 134.9 (Ar*C*), 128.9 (Ar*C*H), 128.3 (Ar*C*H), 127.2 (Ar*C*H), 113.7 (Ar*C*H), 55.6 (CH_2), 54.9 (ArOCH_3), 51.7 (CH_2), 48.7 (*C*H), 32.0 (CH_2), 22.1 (CH_2), 21.8 (CH_2), 21.3 (CH_2), 20.4 (ArCH_3) ppm; IR ($CHCl_3$) ν_{max} = 3431, 2955, 1609, 1511, 1458, 1033, 929 cm^{-1}; HRMS (ESI): *m/z* calcd for $[C_{23}H_{31}NO+H]^+$: 338.2483; found: 338.2508.

Compound 2e:

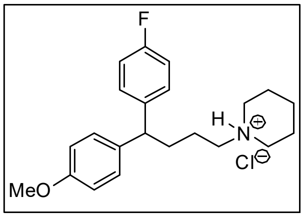

Colorless solid; Yield = 148 mg, (96%); ^{1}H-NMR (500 MHz, DMSO-d_6): δ = 10.21 (bs, 1H, N*H*), 7.34-7.30 (m, 2H, Ar*H*), 7.24-7.21 (m, 2H, Ar*H*), 7.13-7.08 (m, 2H, Ar*H*), 6.88-6.85

(m, 2H, Ar*H*), 3.93 (t, *J* = 8.0 Hz, 1H, C*H*), 3.71 (s, 3H, ArOCH_3), 3.33-3.25 (broad signal, 2H, CH_2), 3.03-2.98 (m, 2H, CH_2), 2.79-2.74 (m, 2H, CH_2), 2.03-1.99 (m, 2H, CH_2), 1.80-1.55 (m, 7H), 1.39-1.30 (m, 1H) ppm; ^{13}C-NMR (125 MHz, DMSO-d_6): δ = 156.5 (d, *J* = 241.2 Hz, Ar*C*F), 153.5 (Ar*C*), 137.2 (Ar*C*), 132.4 (Ar*C*), 125.1 (d, *J* = 8.7 Hz, Ar*C*H), 124.4 (Ar*C*H), 111.0 (d, *J* = 21.2 Hz, Ar*C*H), 109.8 (Ar*C*H), 51.5 (CH_2), 50.9 (ArOCH_3), 47.8 (CH_2), 44.2 (*C*H), 28.0 (CH_2), 18.1 (CH_2), 17.7 (CH_2), 17.3 (CH_2) ppm; IR ($CHCl_3$) ν_{max} = 3448, 2956, 1609, 1509, 668, 625, 579 cm^{-1}; HRMS (ESI): *m/z* calcd for $[C_{22}H_{29}NOF+H]^+$: 342.2233 ; found: 342.2226.

Compound 5a:

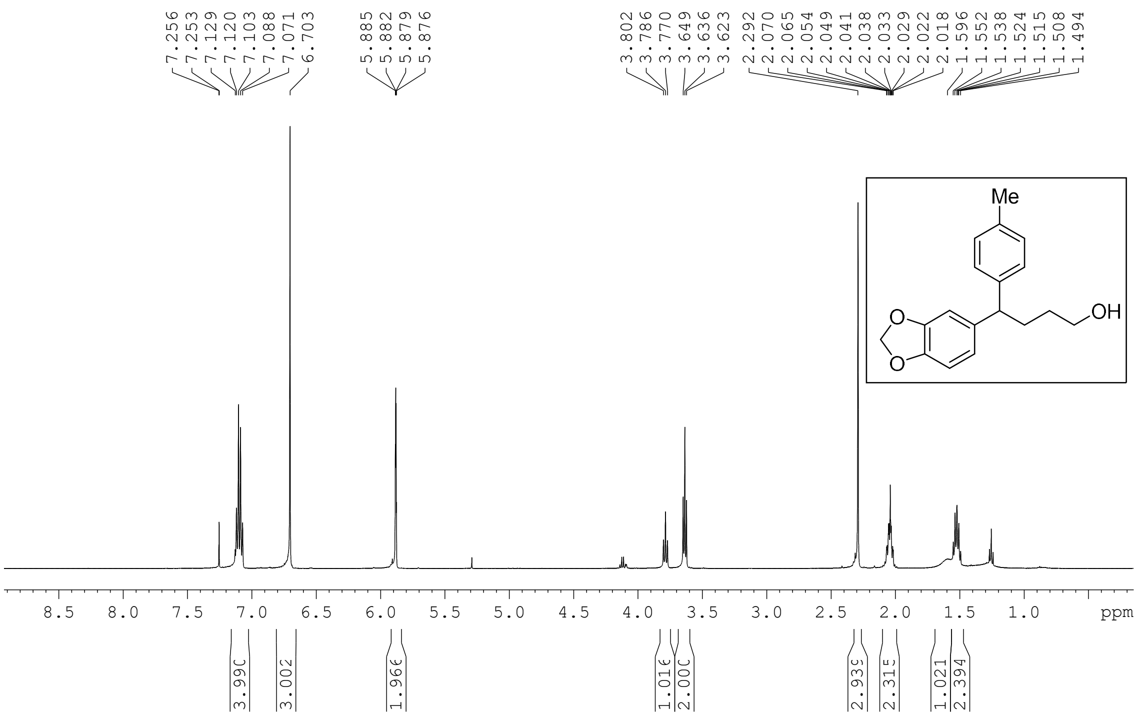

^{1}H NMR ($CDCl_3$, 500 MHz) spectrum of compound 5a

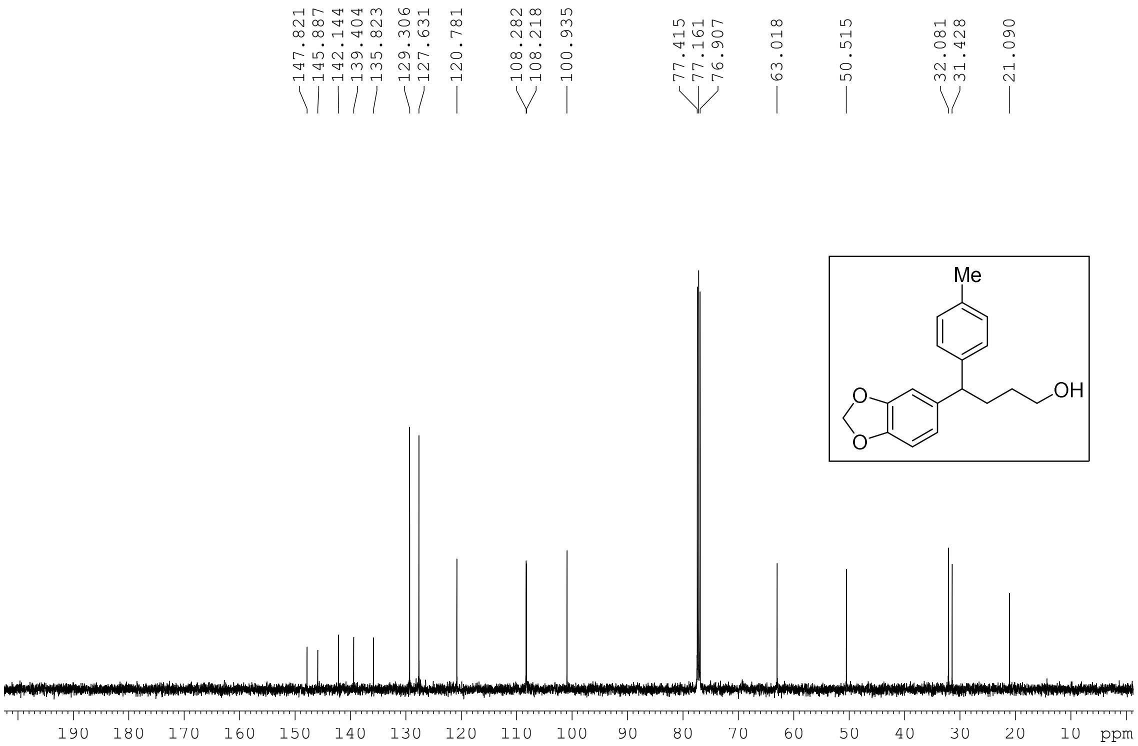

^{13}C NMR ($CDCl_3$, 125 MHz) spectrum of compound 5a

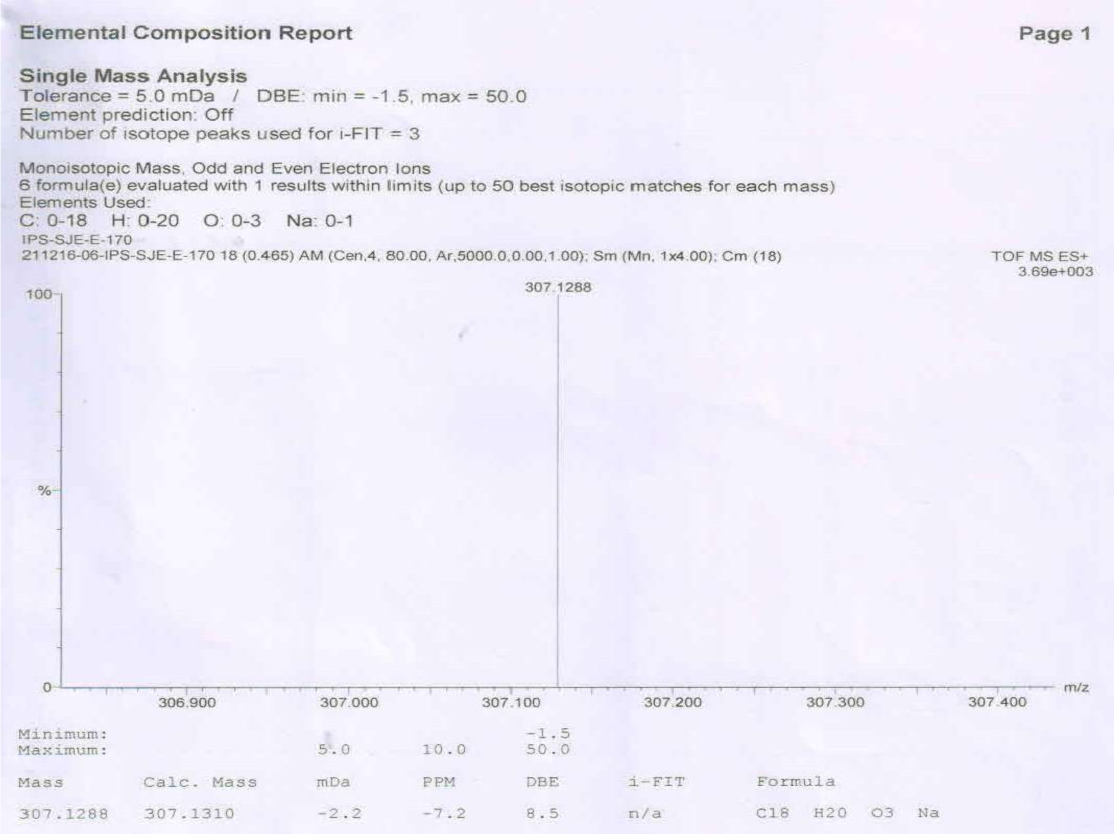

ESI Mass spectrum of compound 5a

Compound 5b:

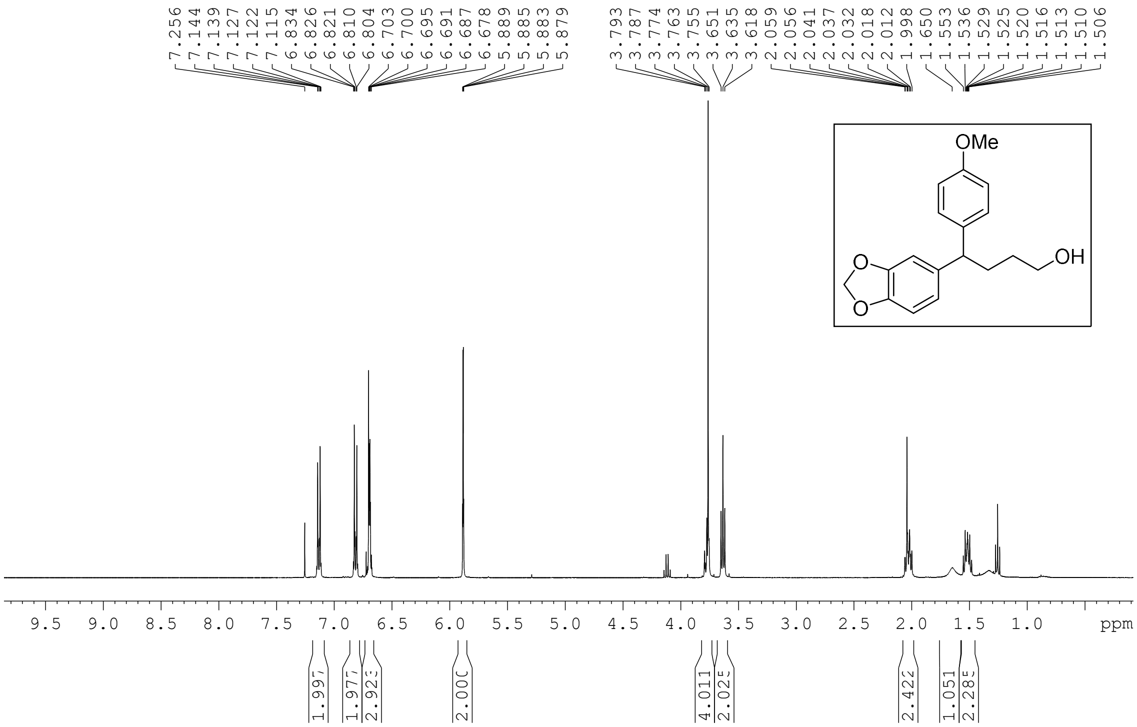

^{1}H NMR ($CDCl_3$, 400 MHz) spectrum of compound 5b

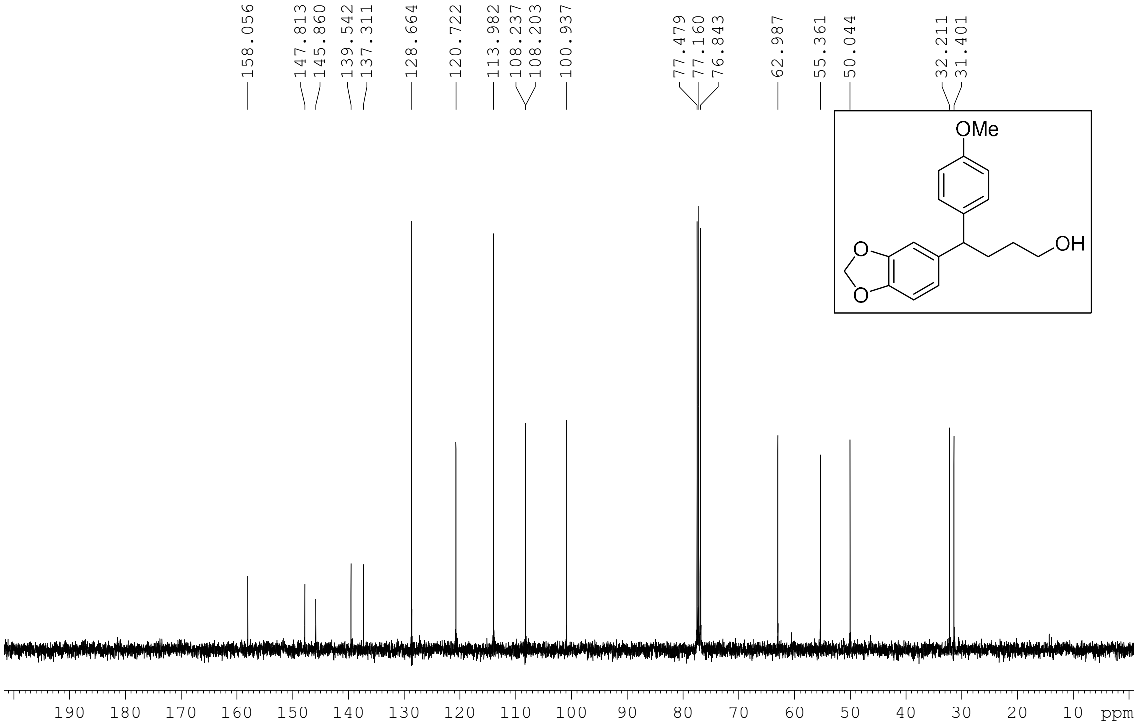

^{1}H NMR ($CDCl_3$, 400 MHz) spectrum of compound 5b

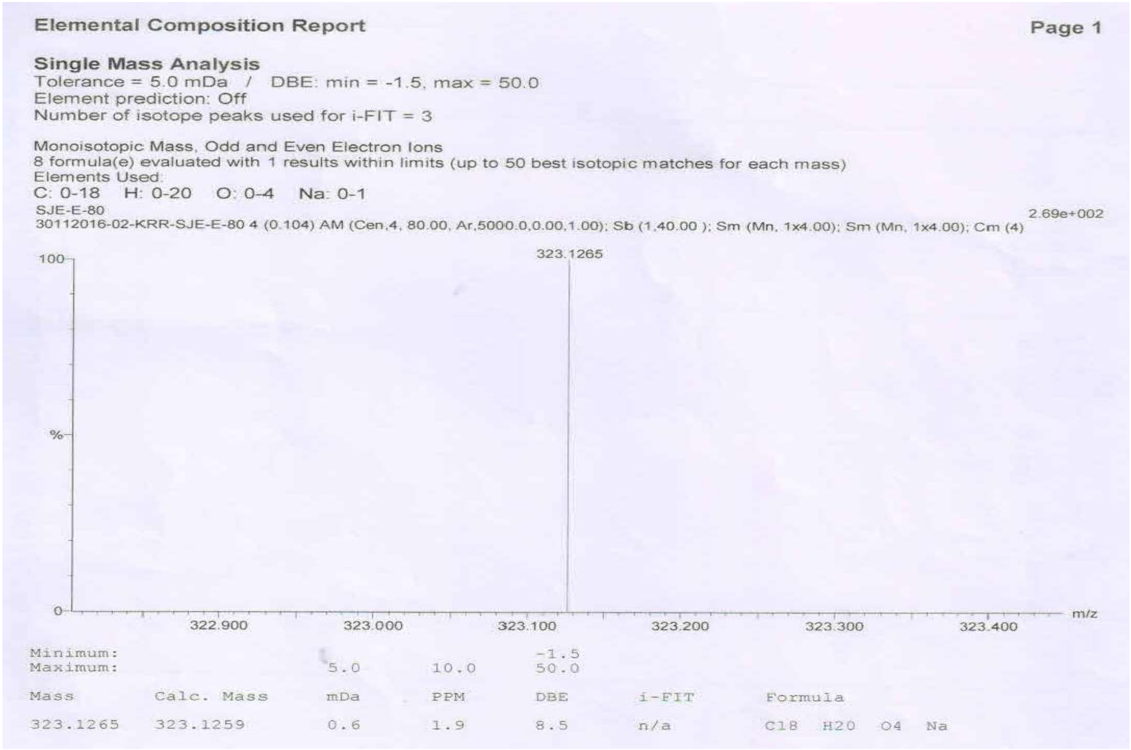

ESI Mass spectrum of compound 5b

Compound 5d:

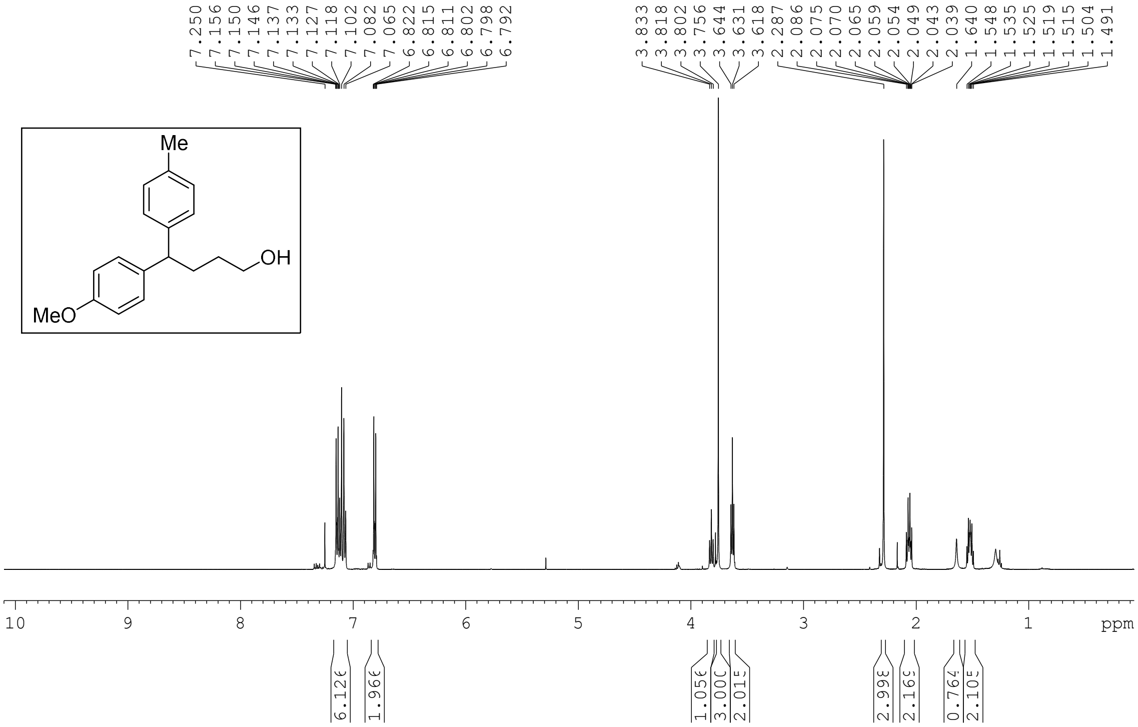

^{1}H NMR ($CDCl_3$, 500 MHz) spectrum of compound 5d

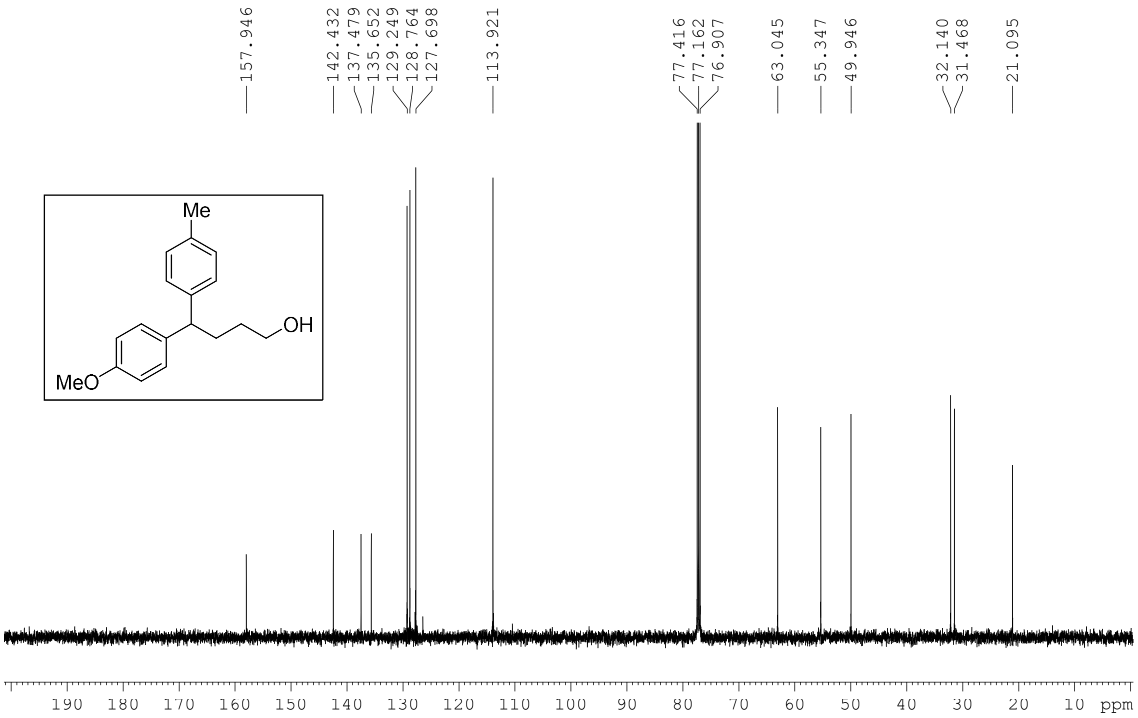

^{13}C NMR (CDCl$_3$, 125 MHz) spectrum of compound 5d

Compound 5e:

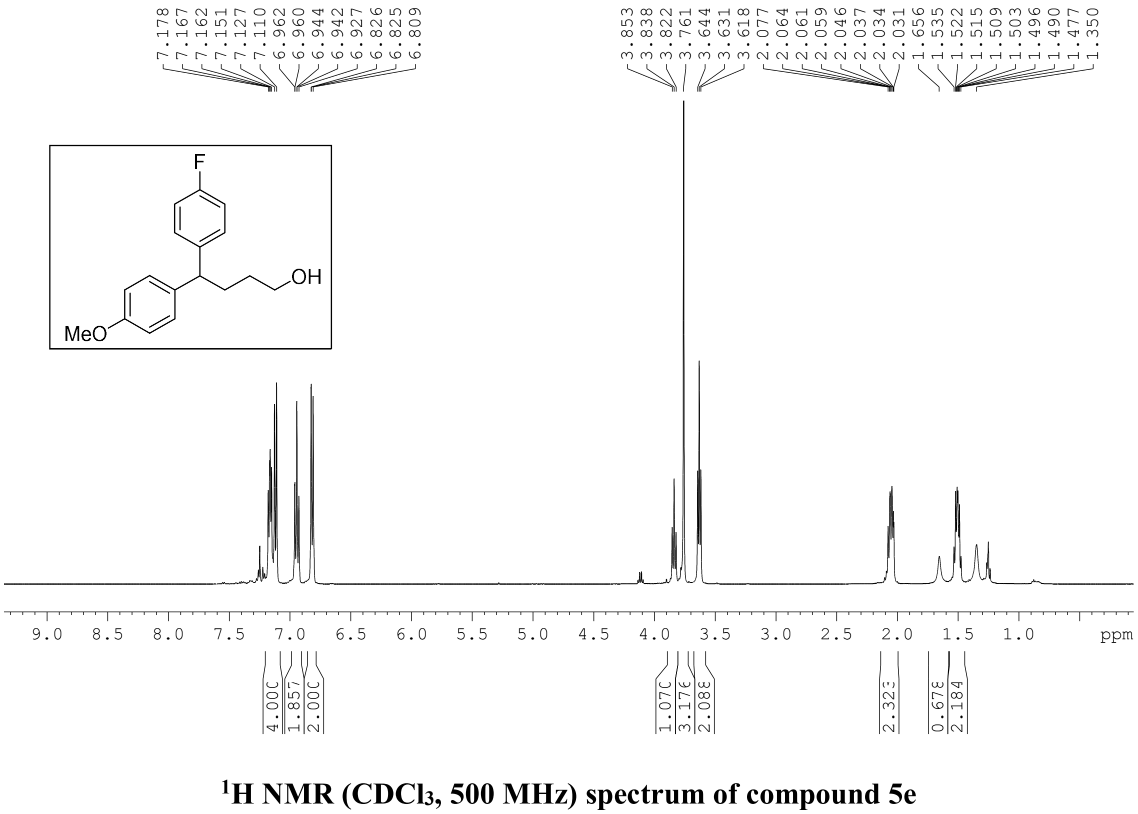

^{1}H NMR (CDCl$_3$, 500 MHz) spectrum of compound 5e

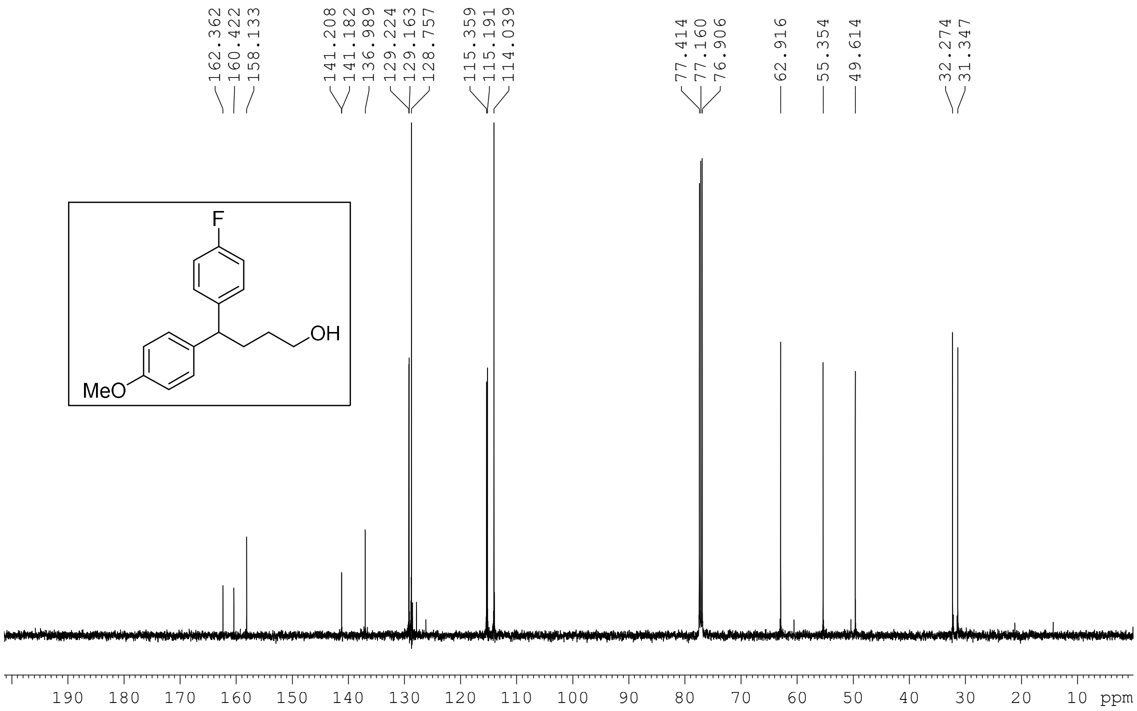

^{13}C NMR ($CDCl_3$, 125 MHz) spectrum of compound 5e

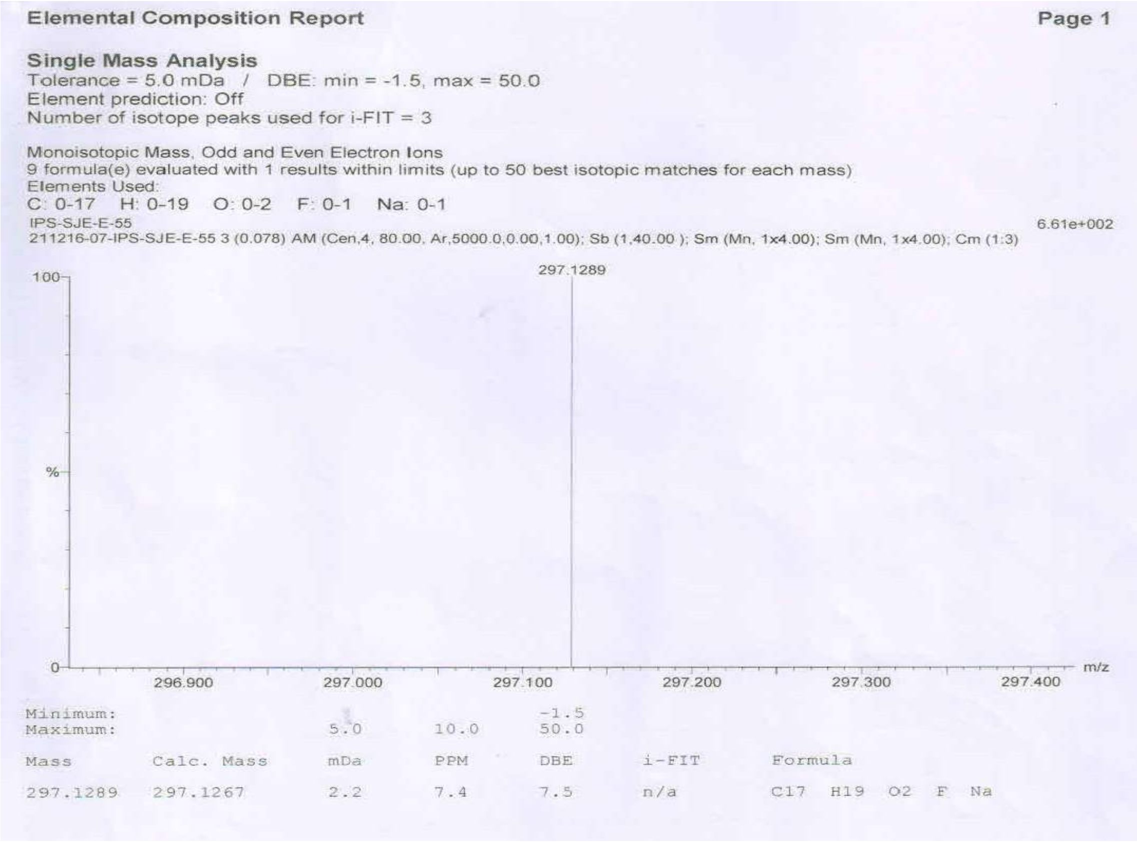

ESI Mass spectrum of compound 5e

Compound 6a:

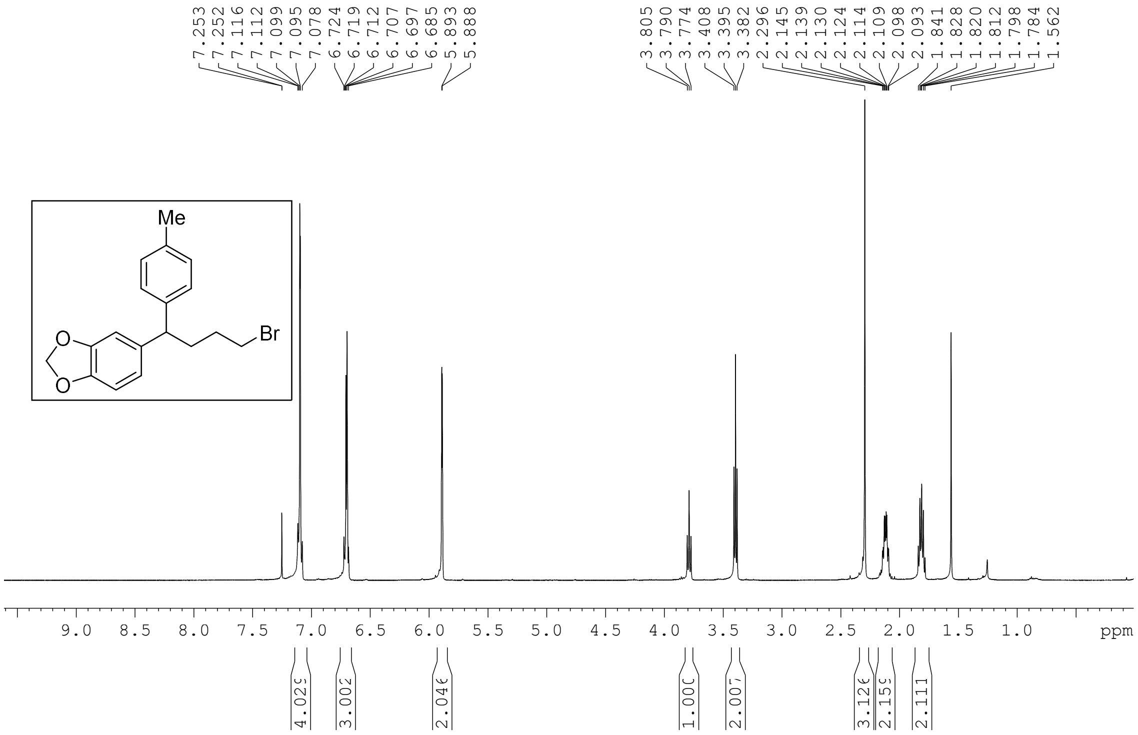

^{1}H NMR (CDCl$_3$, 500 MHz) spectrum of compound 6a

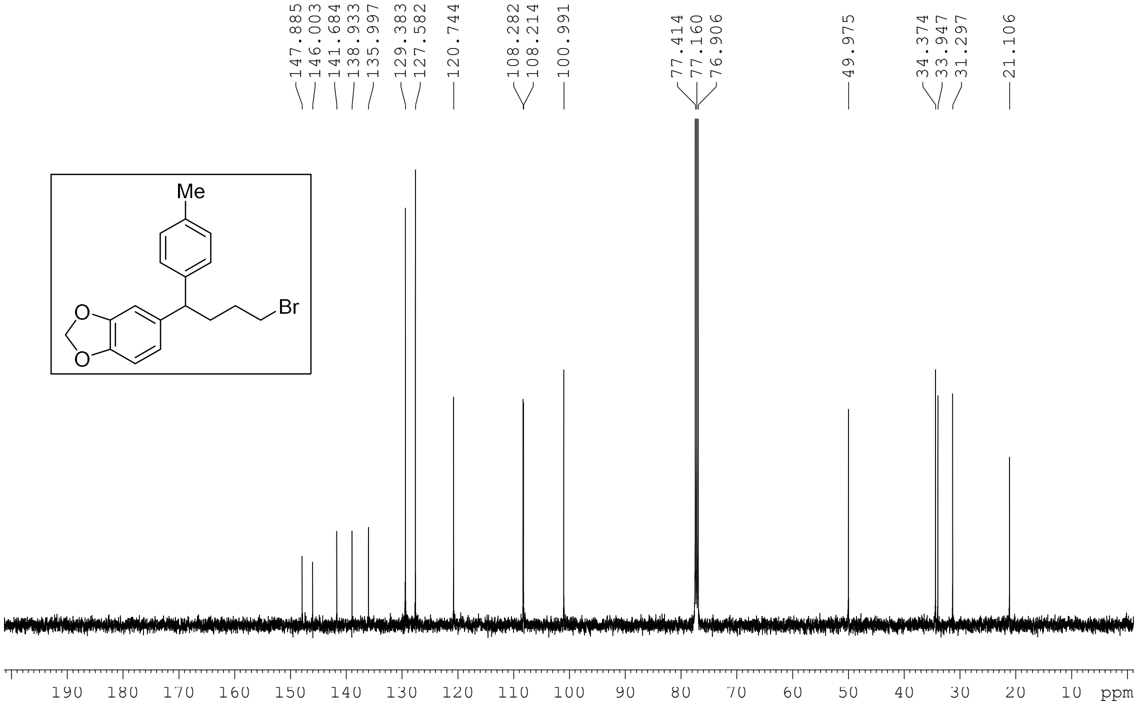

^{13}C NMR (CDCl$_3$, 125 MHz) spectrum of compound 6a

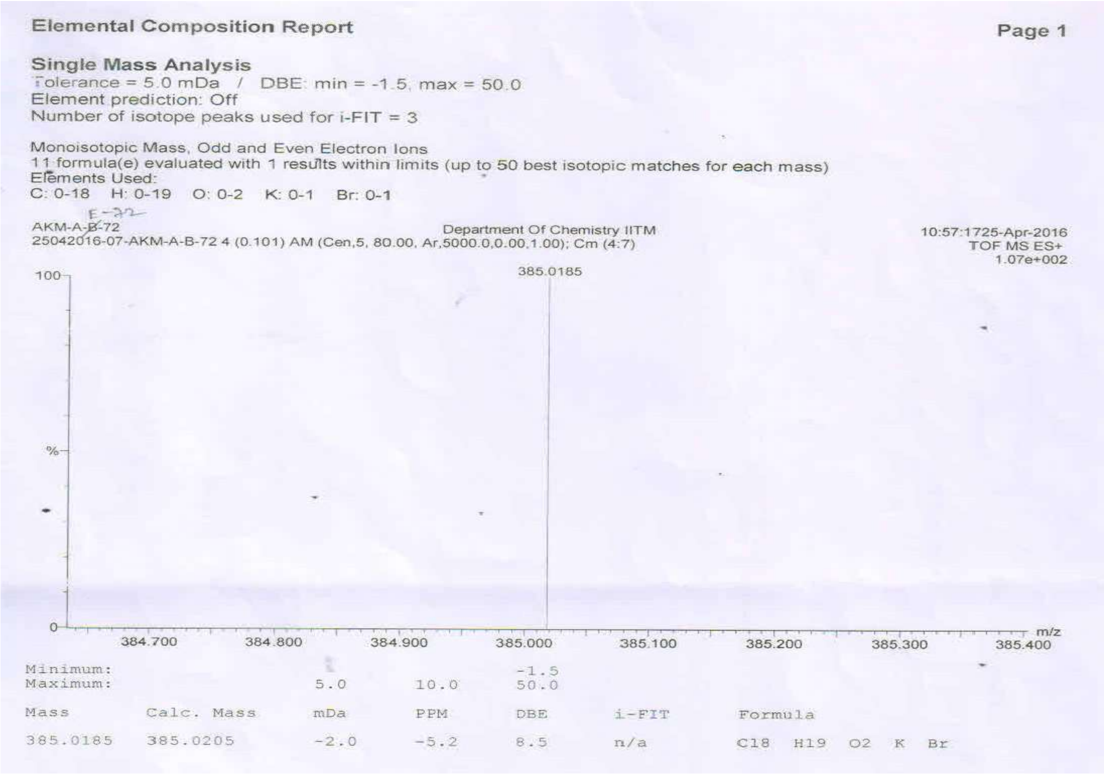

ESI Mass spectrum of compound 6a

Compound 6b:

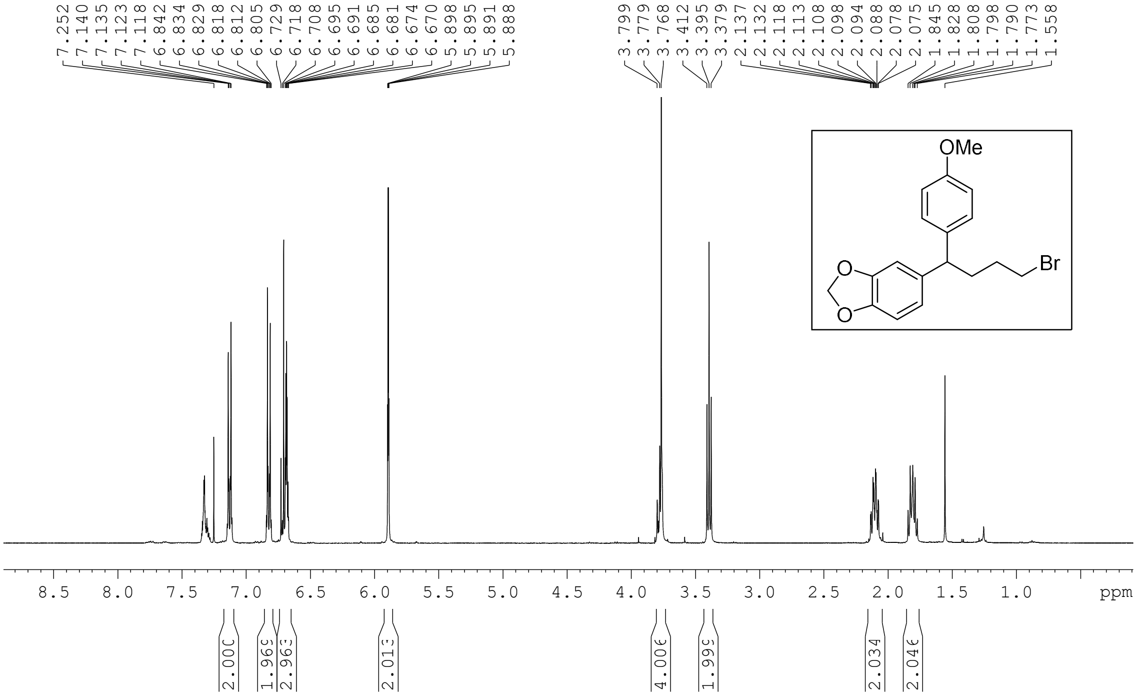

^{1}H NMR ($CDCl_3$, 400 MHz) spectrum of compound 6b

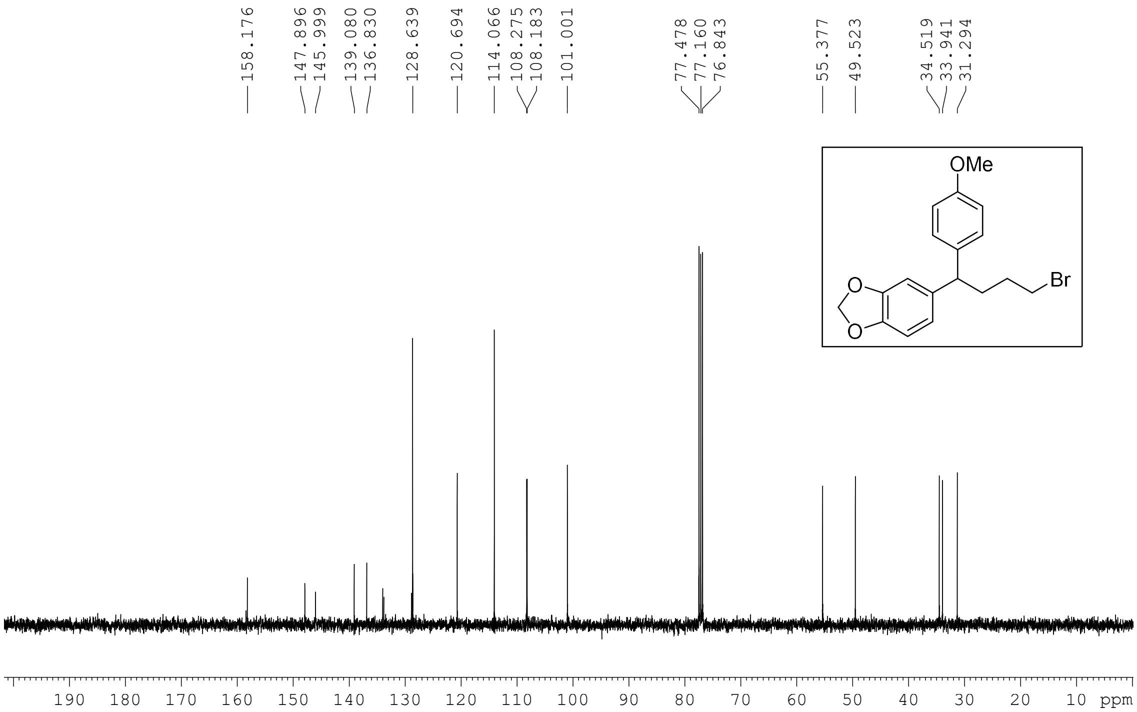

^{13}C NMR ($CDCl_3$, 100 MHz) spectrum of compound 6b

Compound 6c:

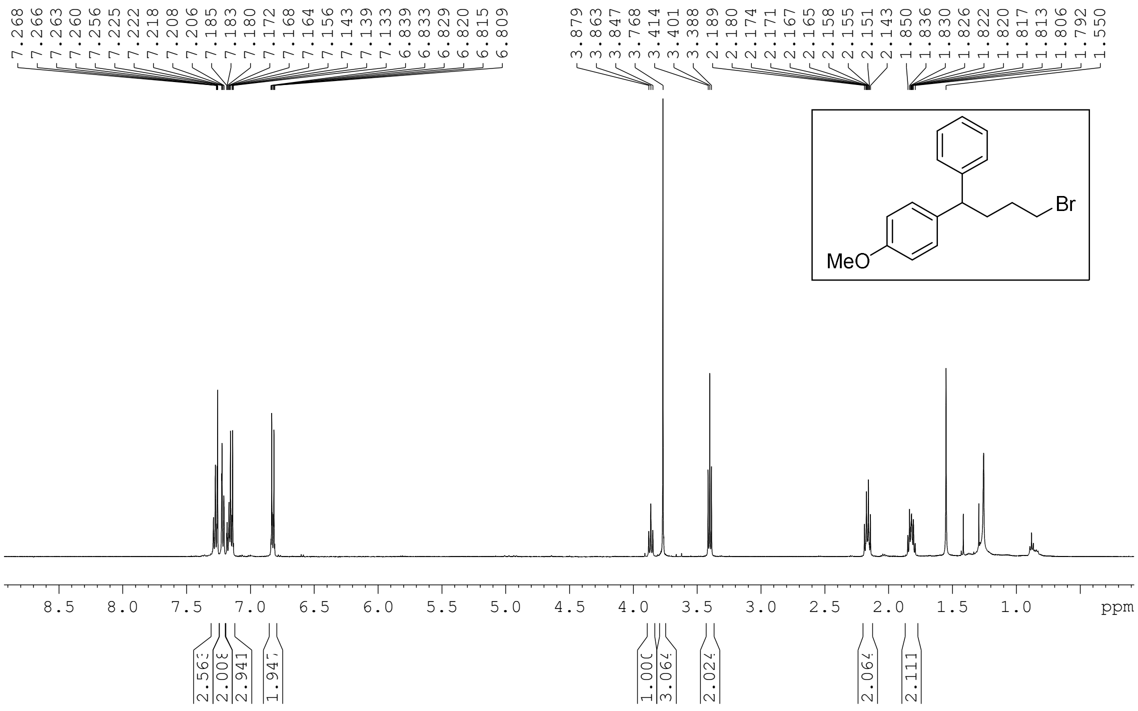

^{1}H NMR ($CDCl_3$, 500 MHz) spectrum of compound 6c

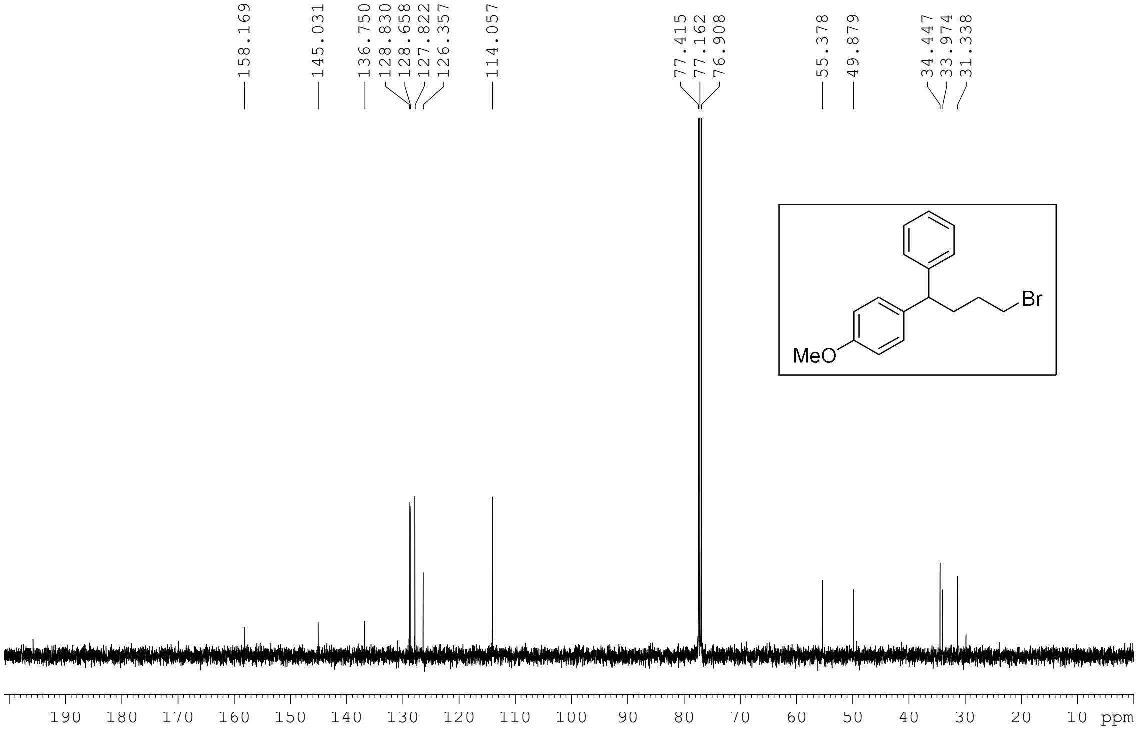

^{13}C NMR ($CDCl_3$, 125 MHz) spectrum of compound 6c

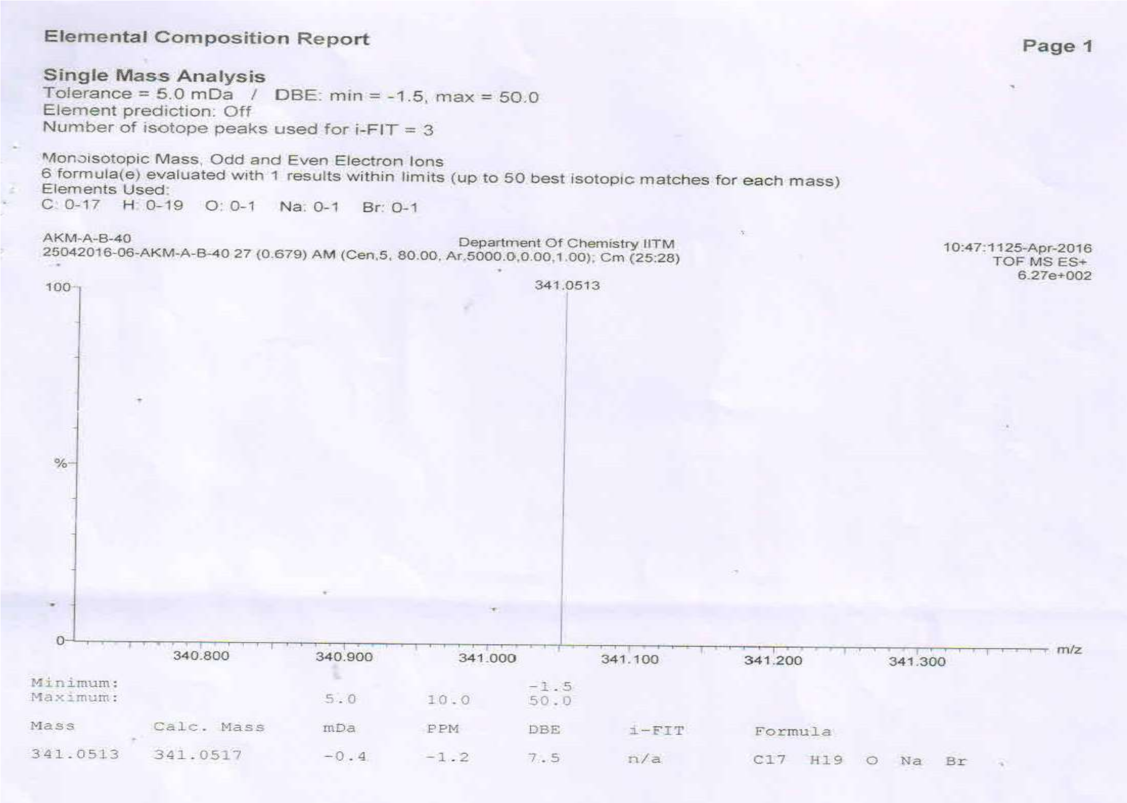

ESI Mass spectrum of compound 6c

Compound 6d:

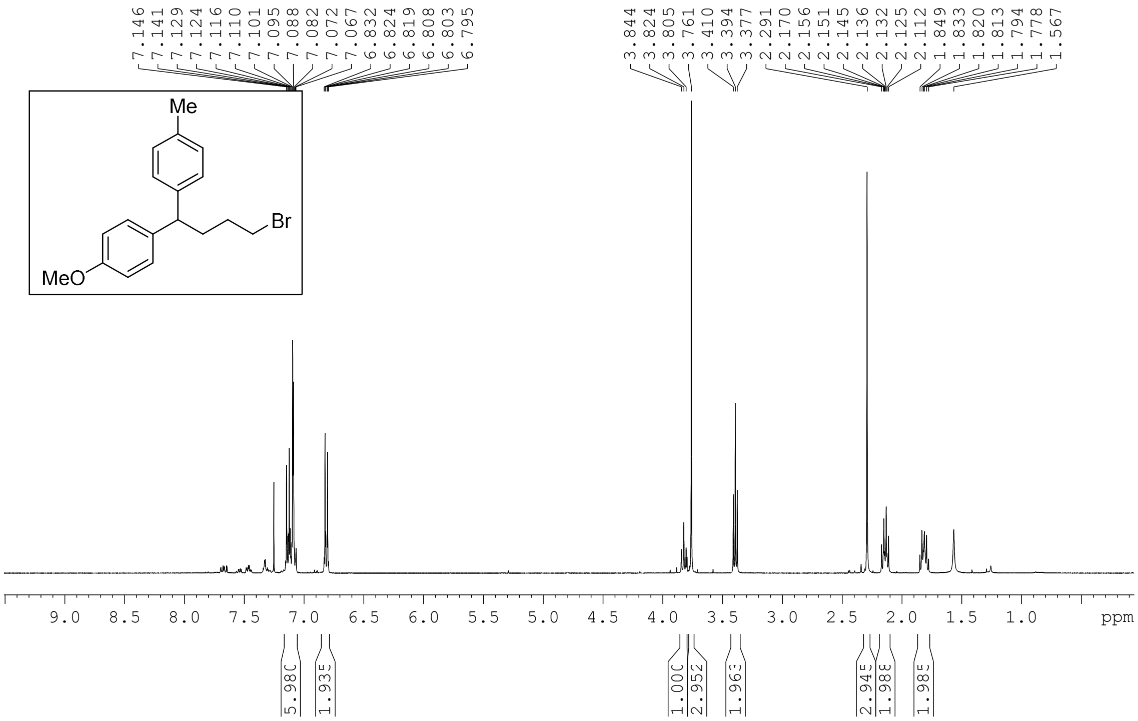

^{1}H NMR (CDCl$_3$, 400 MHz) spectrum of compound 6d

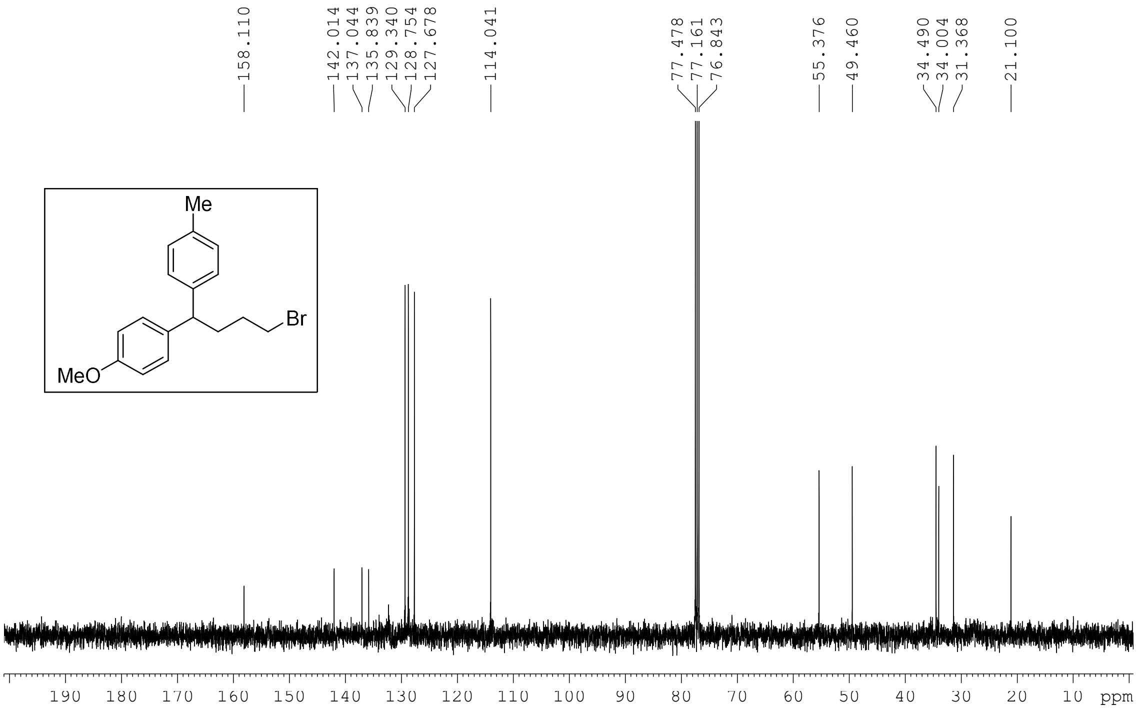

^{13}C NMR (CDCl$_3$, 100 MHz) spectrum of compound 6d

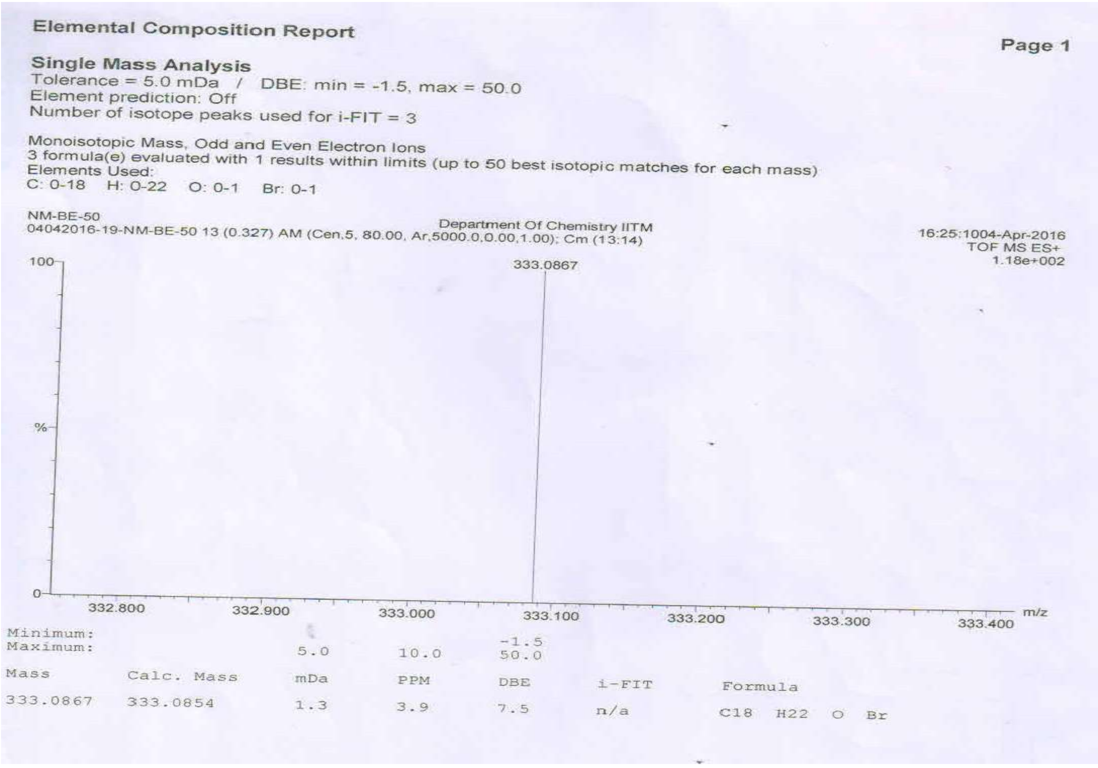

Elemental Composition Report

Page 1

Single Mass Analysis
Tolerance = 5.0 mDa / DBE: min = -1.5, max = 50.0
Element prediction: Off
Number of isotope peaks used for i-FIT = 3

Monoisotopic Mass, Odd and Even Electron Ions
3 formula(e) evaluated with 1 results within limits (up to 50 best isotopic matches for each mass)
Elements Used:
C: 0-18 H: 0-22 O: 0-1 Br: 0-1

NM-BE-50
04042016-19-NM-BE-50 13 (0.327) AM (Cen,5, 80.00, Ar,5000.0,0.00,1.00); Cm (13:14)
Department Of Chemistry IITM
16:25:1004-Apr-2016
TOF MS ES+
1.18e+002

Minimum:				-1.5		
Maximum:		5.0	10.0	50.0		
Mass	Calc. Mass	mDa	PPM	DBE	i-FIT	Formula
333.0867	333.0854	1.3	3.9	7.5	n/a	C18 H22 O Br

ESI Mass spectrum of compound 6d

Compound 6e:

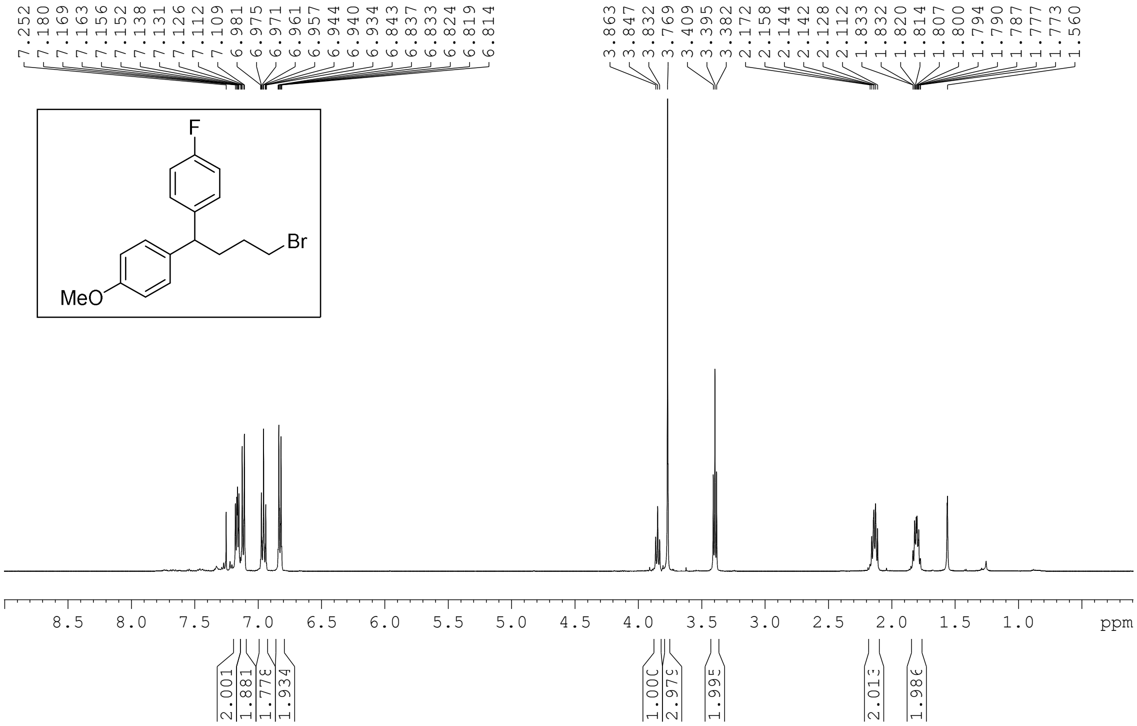

^{1}H NMR ($CDCl_3$, 500 MHz) spectrum of compound 6e

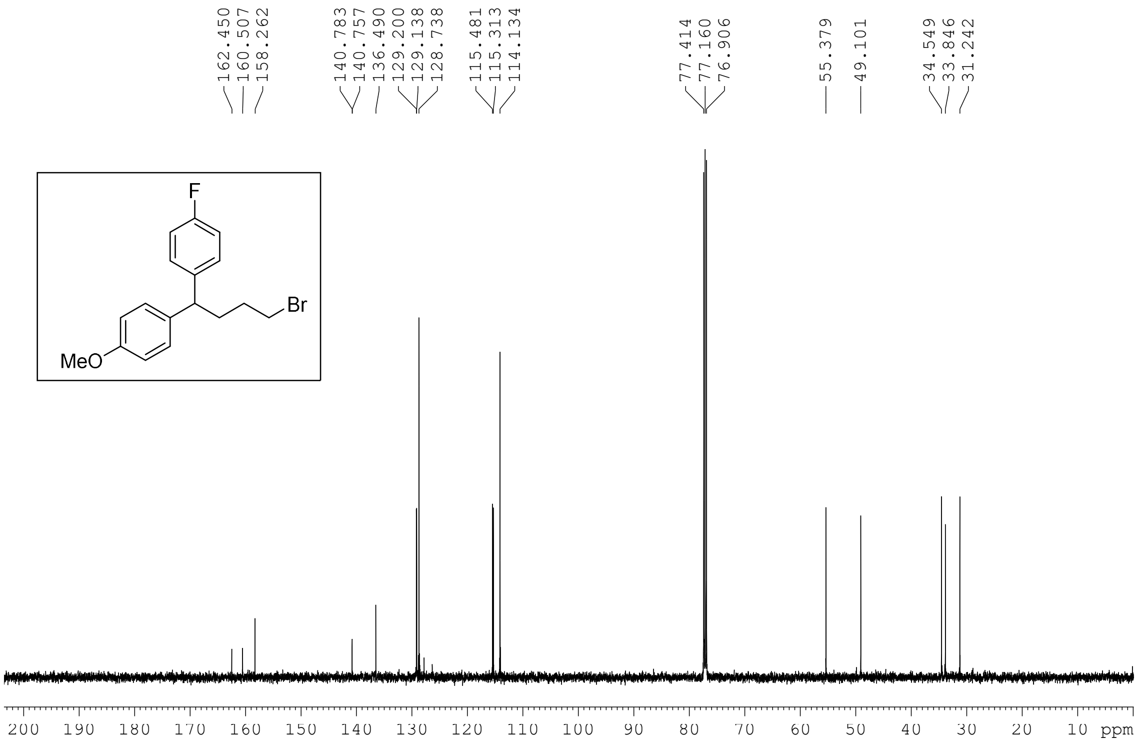

^{13}C NMR ($CDCl_3$, 125 MHz) spectrum of compound 6e

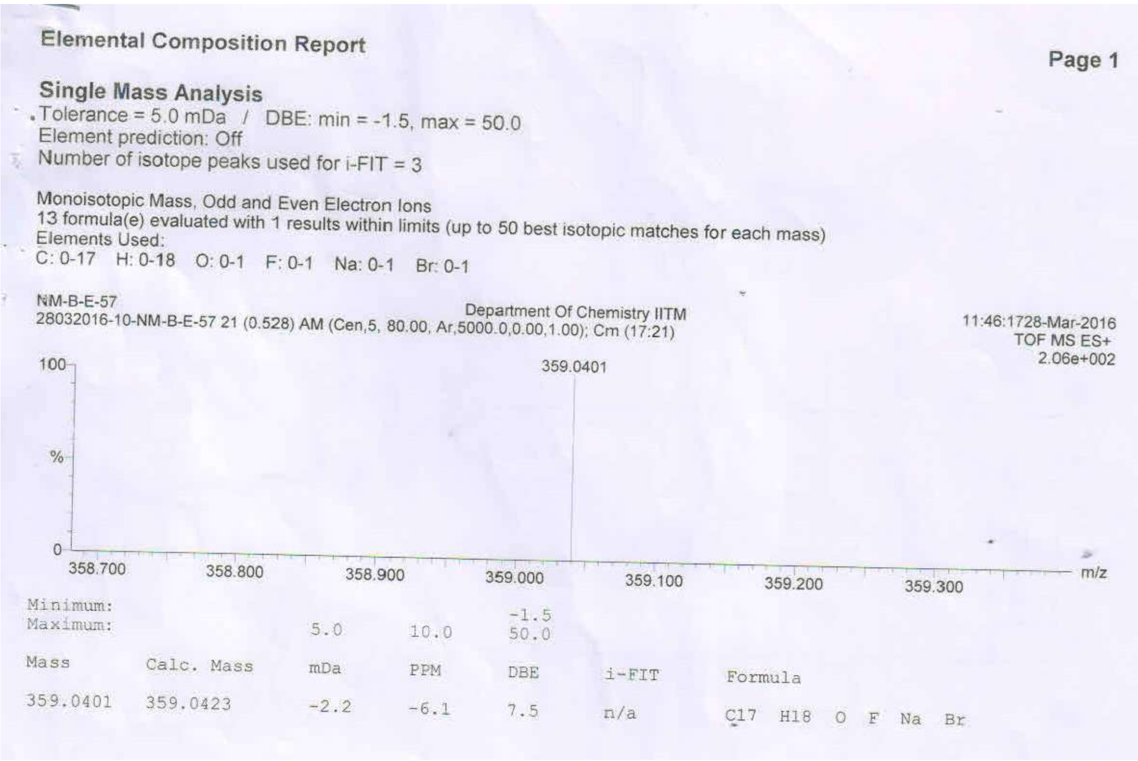

Elemental Composition Report

Page 1

Single Mass Analysis
Tolerance = 5.0 mDa / DBE: min = -1.5, max = 50.0
Element prediction: Off
Number of isotope peaks used for i-FIT = 3

Monoisotopic Mass, Odd and Even Electron Ions
13 formula(e) evaluated with 1 results within limits (up to 50 best isotopic matches for each mass)
Elements Used:
C: 0-17 H: 0-18 O: 0-1 F: 0-1 Na: 0-1 Br: 0-1

NM-B-E-57
28032016-10-NM-B-E-57 21 (0.528) AM (Cen,5, 80.00, Ar,5000.0,0.00,1.00); Cm (17:21)
Department Of Chemistry IITM
11:46:1728-Mar-2016
TOF MS ES+
2.06e+002

Minimum:				-1.5		
Maximum:		5.0	10.0	50.0		
Mass	Calc. Mass	mDa	PPM	DBE	i-FIT	Formula
359.0401	359.0423	-2.2	-6.1	7.5	n/a	C17 H18 O F Na Br

ESI Mass spectrum of compound 6e

Compound 7a:

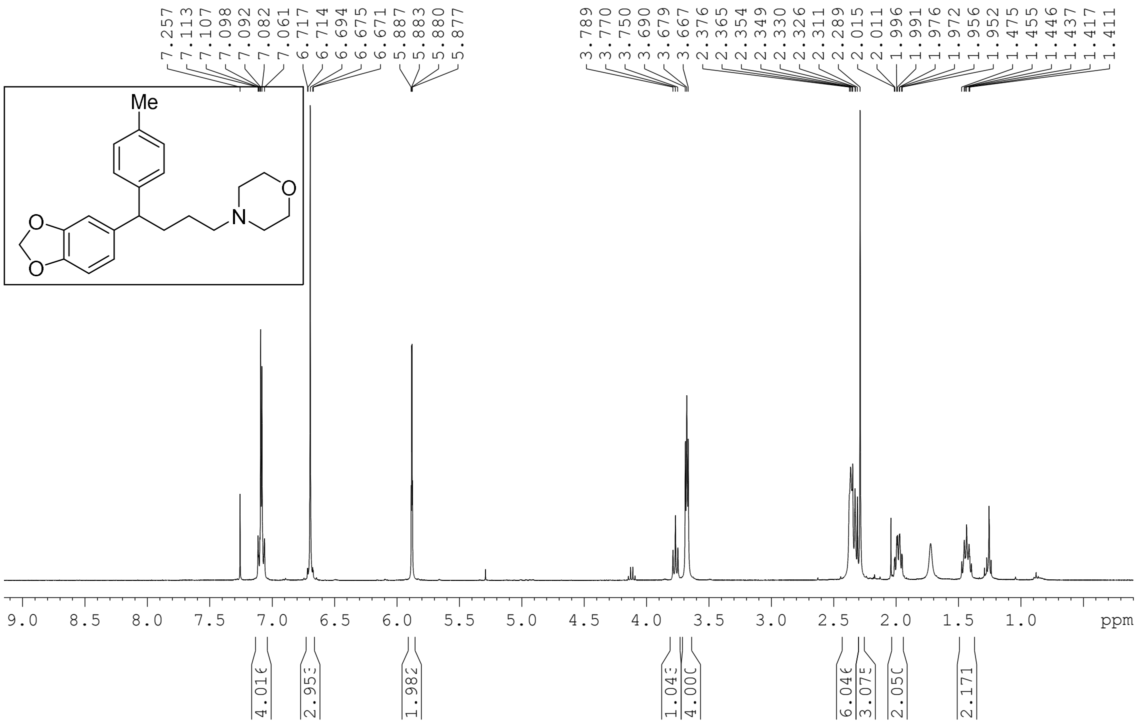

^{1}H NMR ($CDCl_3$, 400 MHz) spectrum of compound 7a

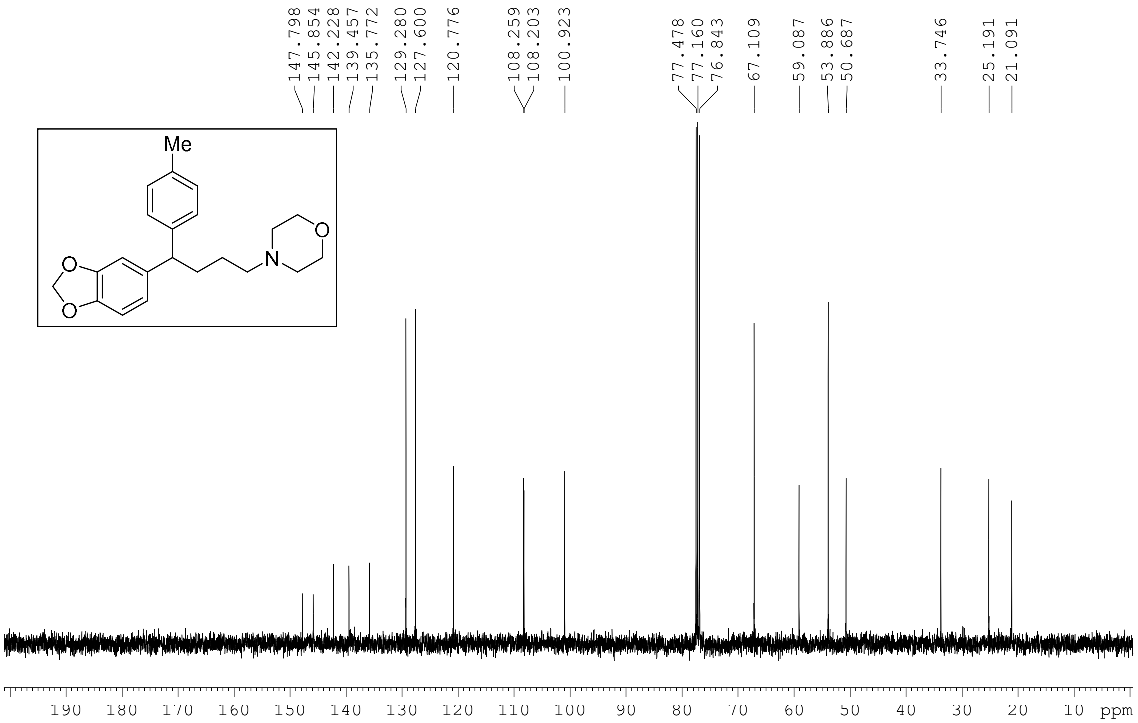

^{13}C NMR ($CDCl_3$, 100 MHz) spectrum of compound 7a

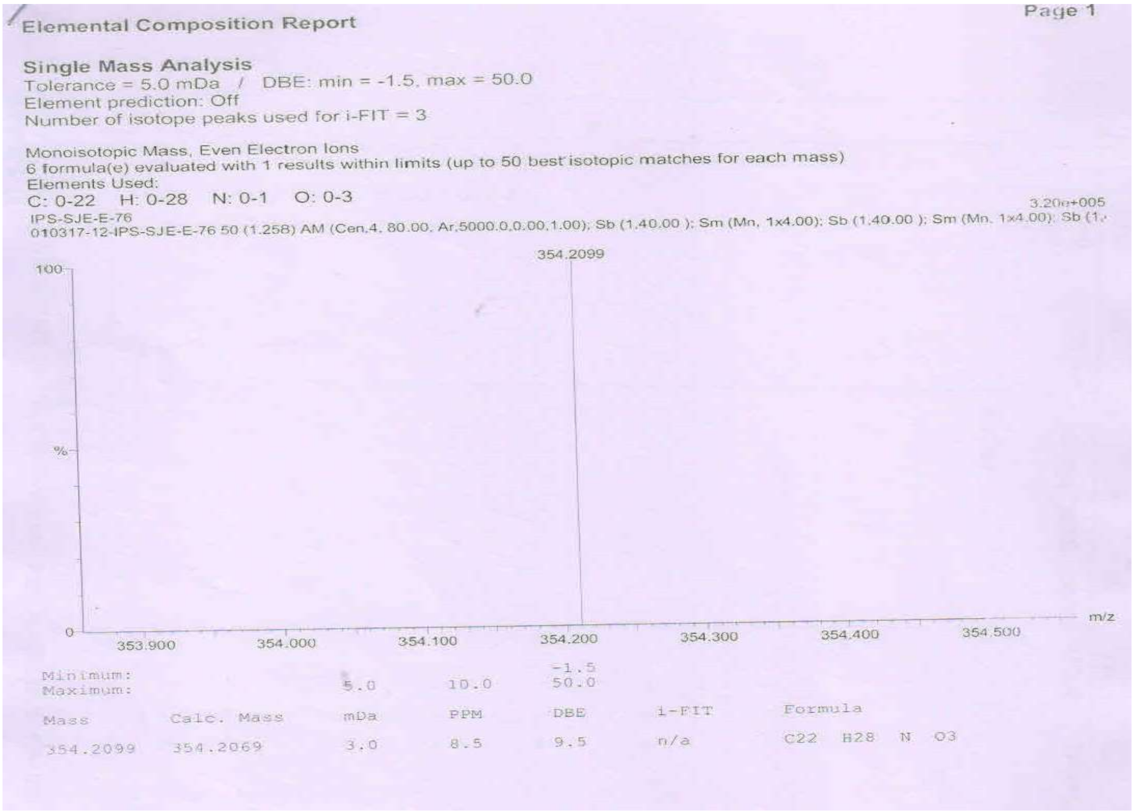

ESI Mass spectrum of compound 7a

Compound 7b:

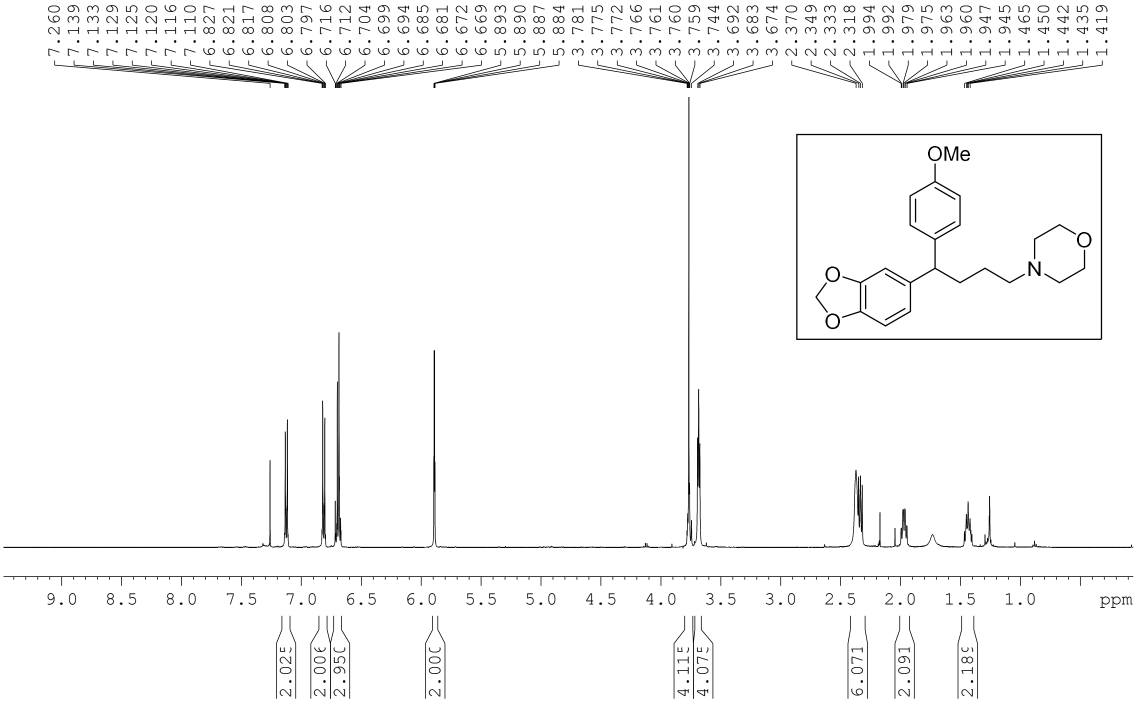

^{1}H NMR (CDCl$_3$, 500 MHz) spectrum of compound 7b

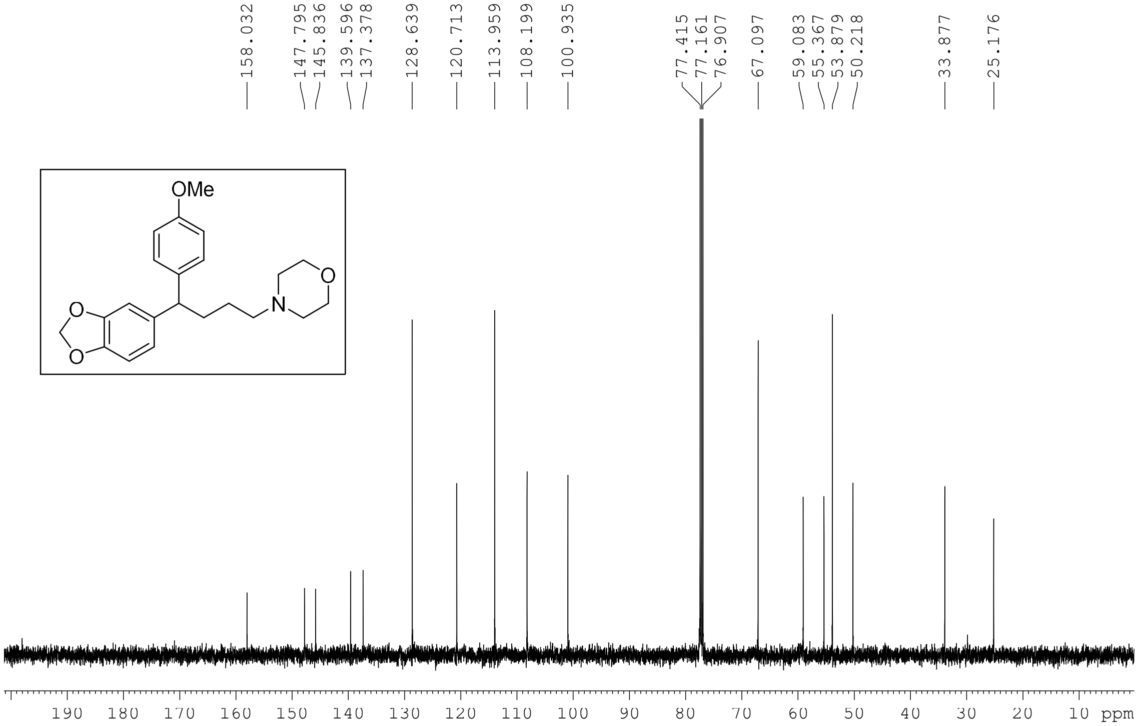

^{13}C NMR ($CDCl_3$, 125 MHz) spectrum of compound 7b

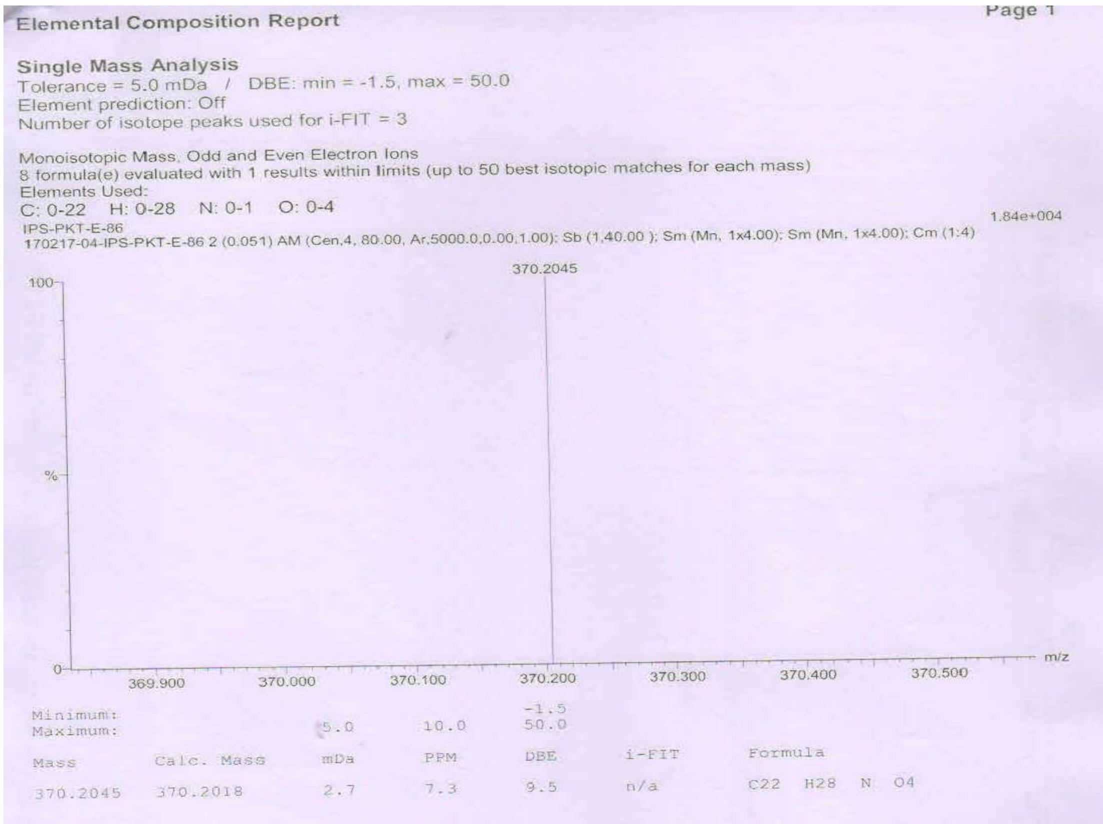

ESI Mass spectrum of compound 7b

Compound 7c:

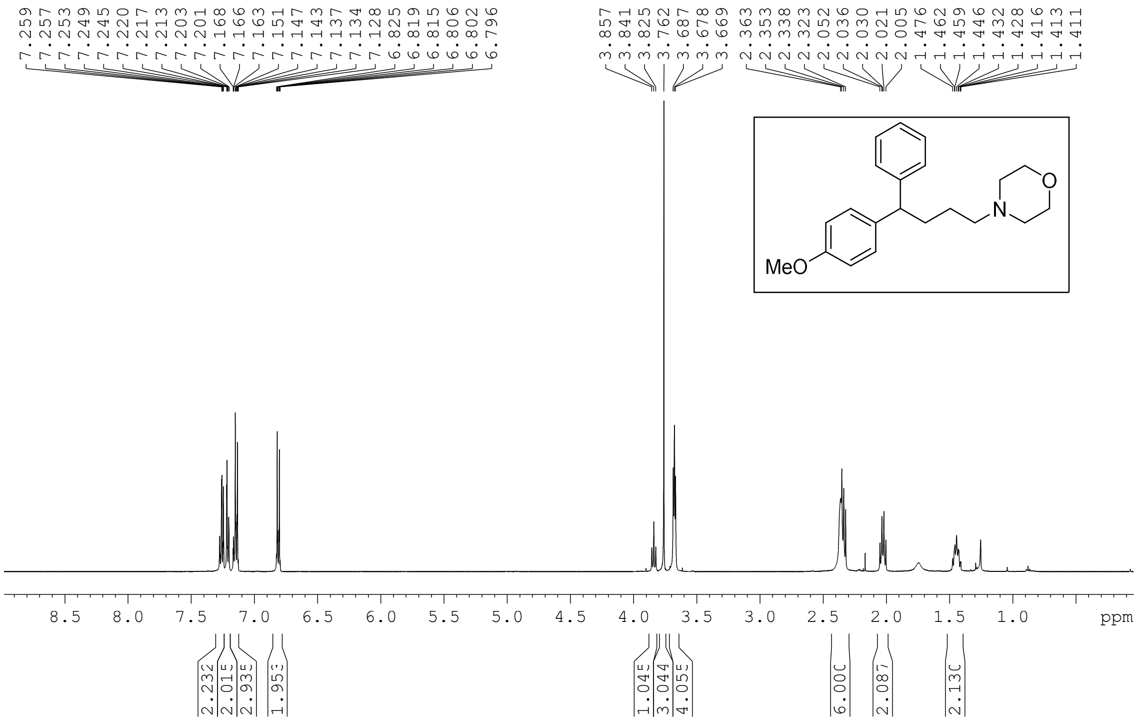

^{1}H NMR (CDCl₃, 500 MHz) spectrum of compound 7c

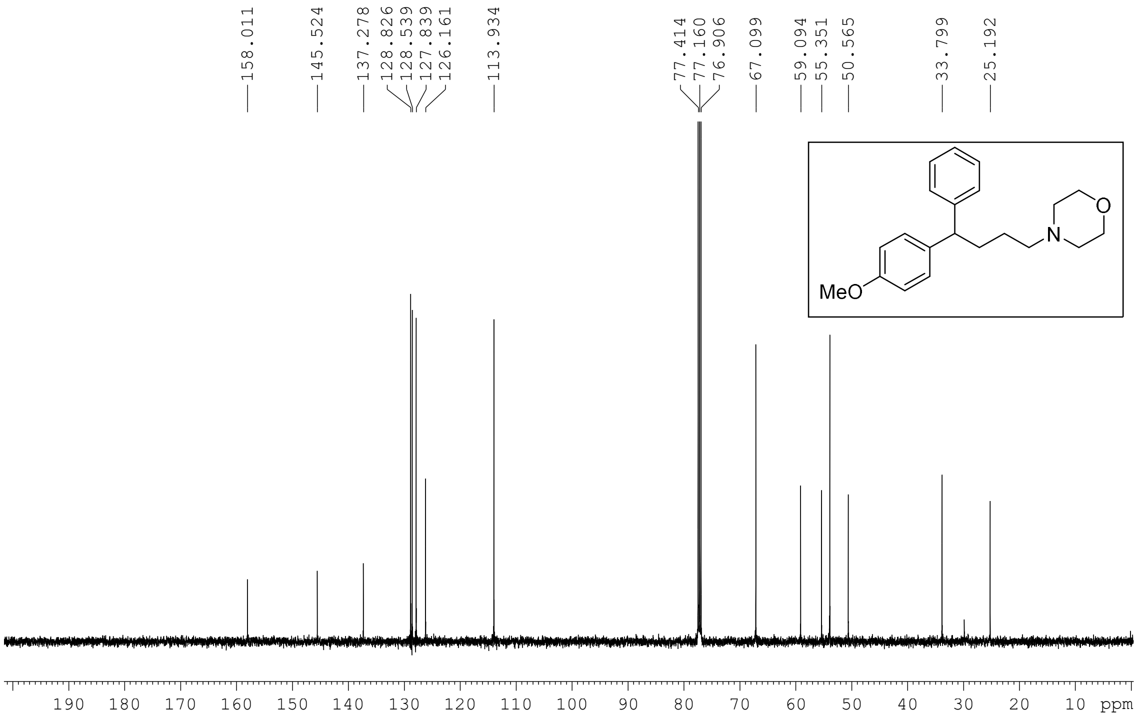

^{13}C NMR (CDCl₃, 125 MHz) spectrum of compound 7c

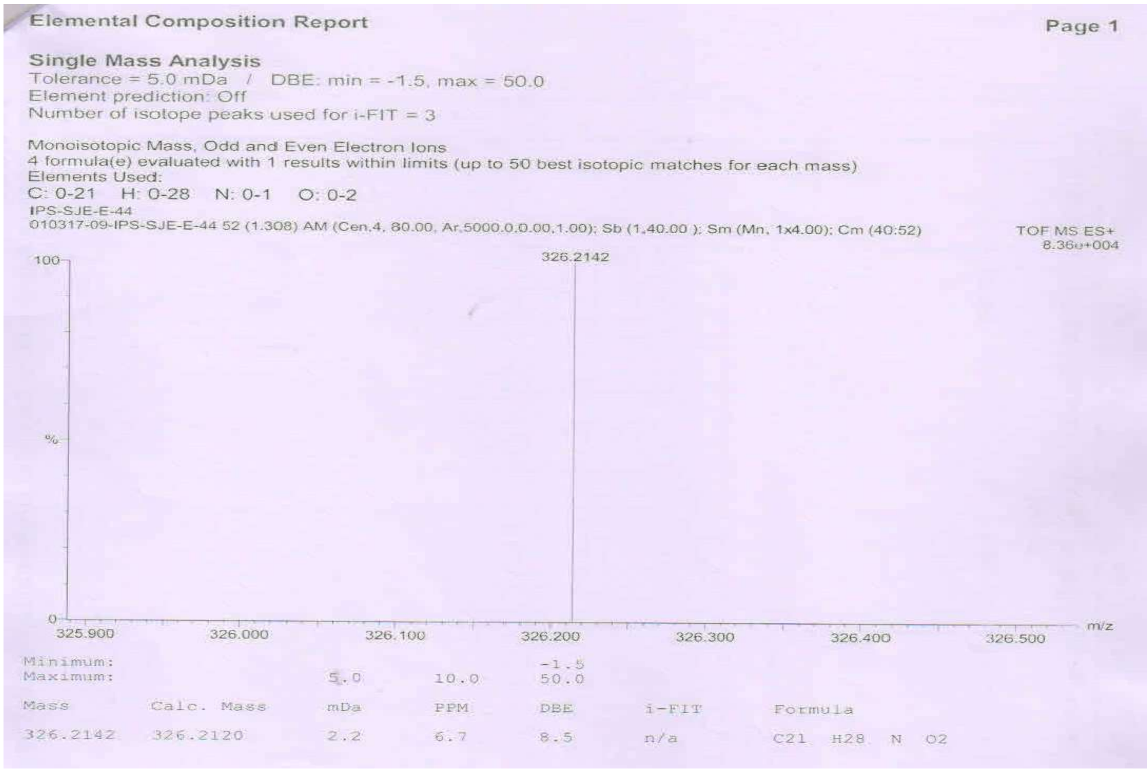

ESI Mass spectrum of compound 7c

Compound 7d:

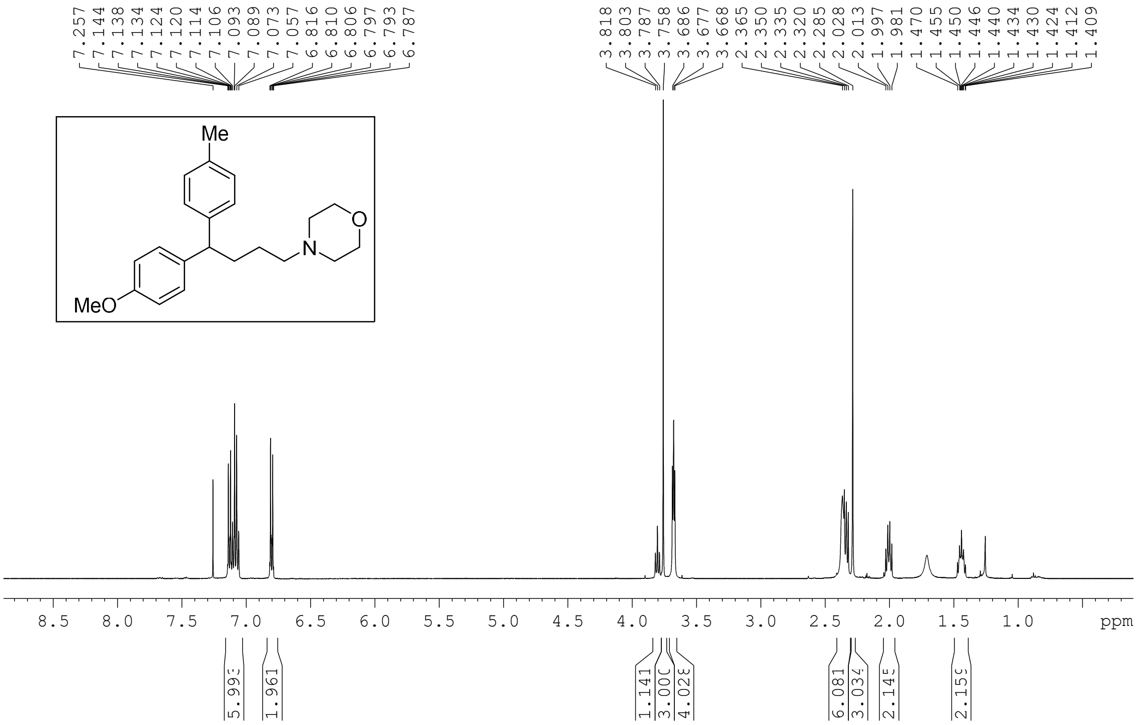

^{1}H NMR ($CDCl_3$, 500 MHz) spectrum of compound 7d

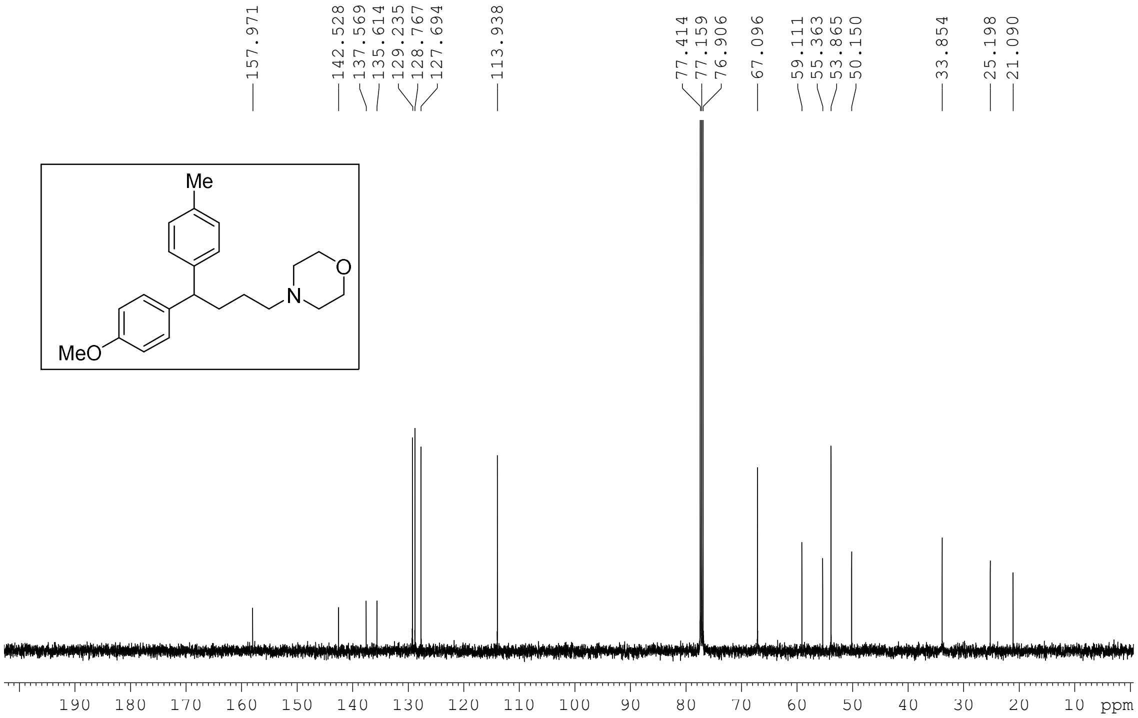

^{13}C NMR ($CDCl_3$, 125 MHz) spectrum of compound 7d

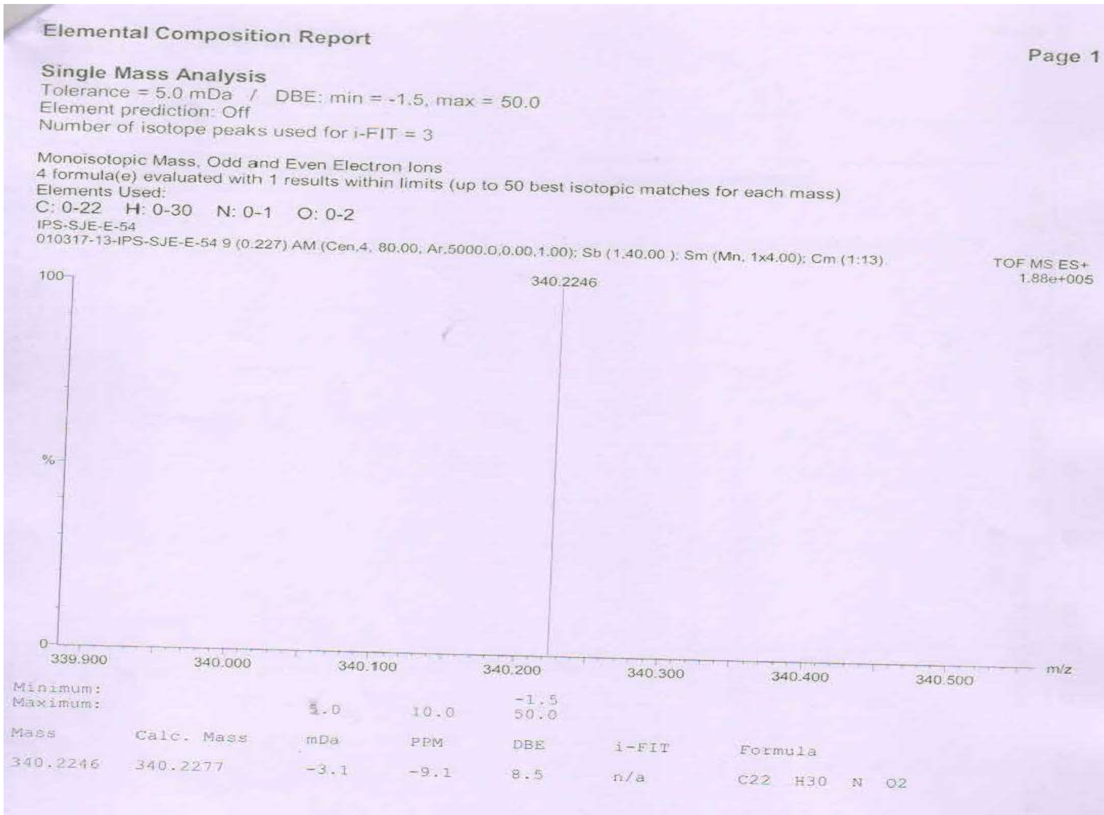

ESI Mass spectrum of compound 7d

Compound 7e:

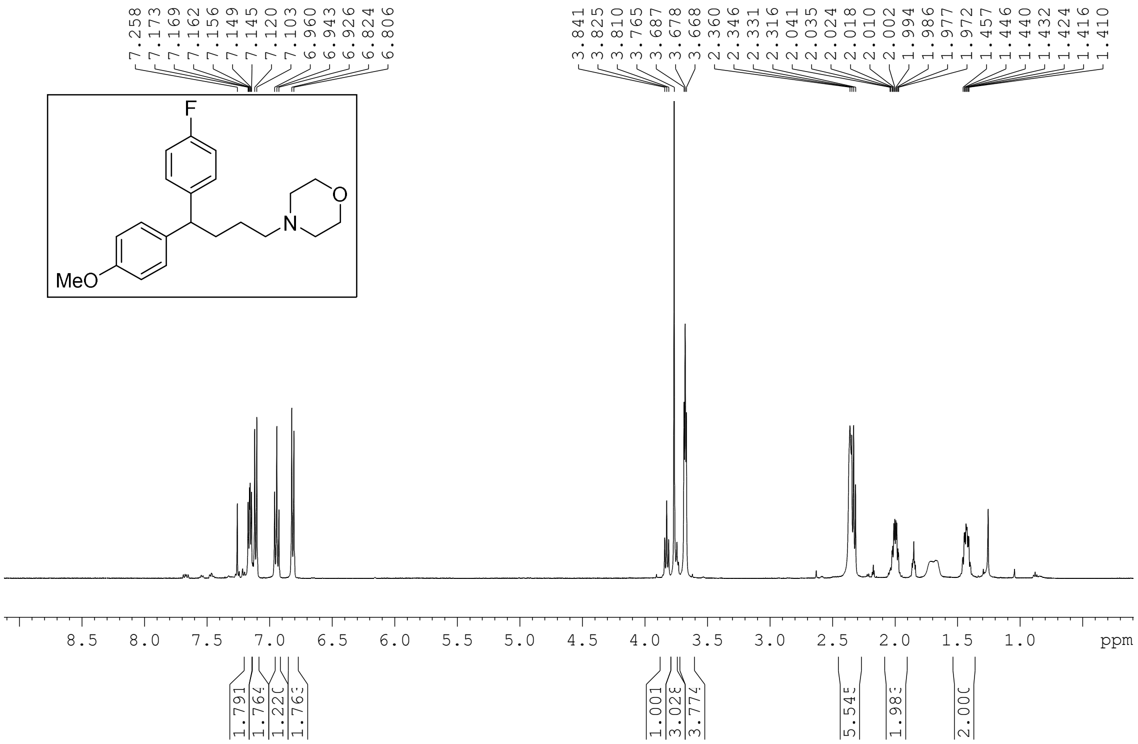

^1H NMR ($CDCl_3$, 500 MHz) spectrum of compound 7e

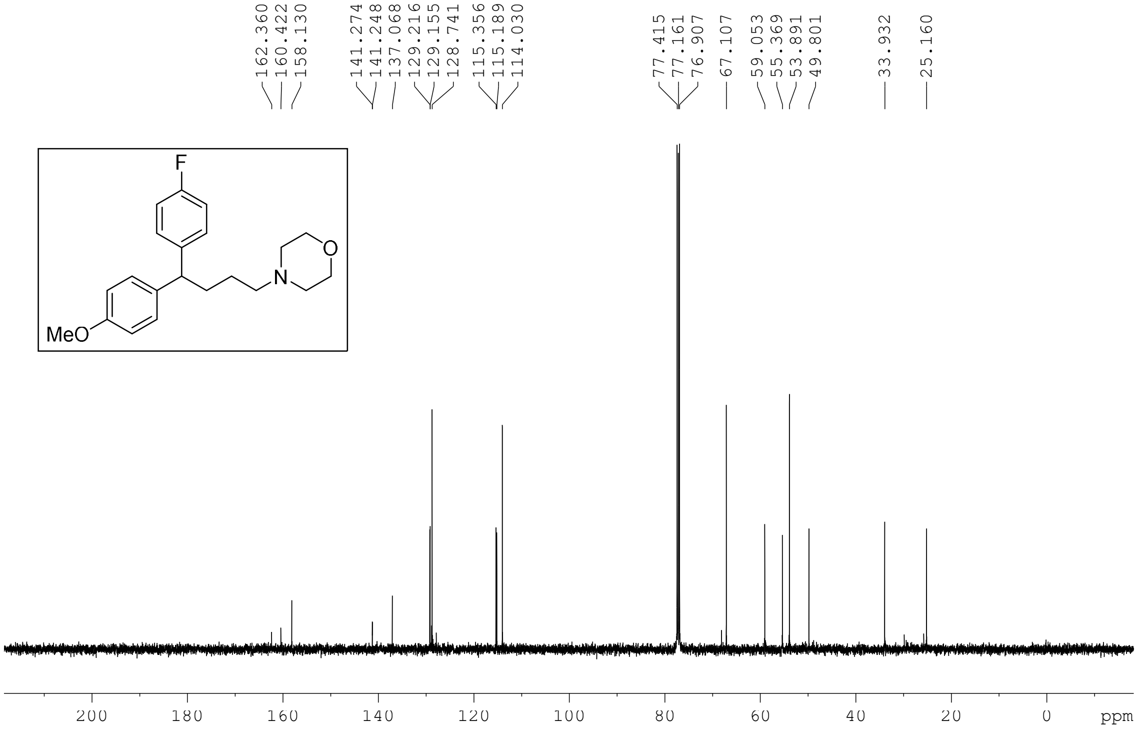

^{13}C NMR ($CDCl_3$, 125 MHz) spectrum of compound 7e

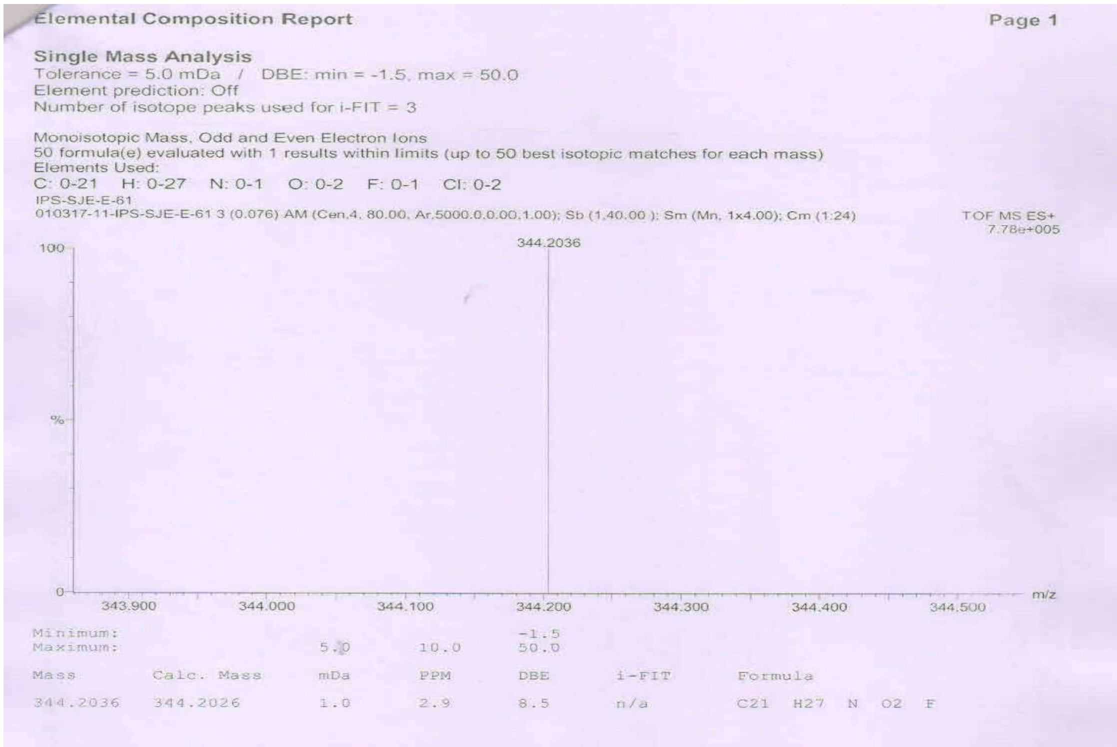

ESI Mass spectrum of compound 7e

Compound 8a:

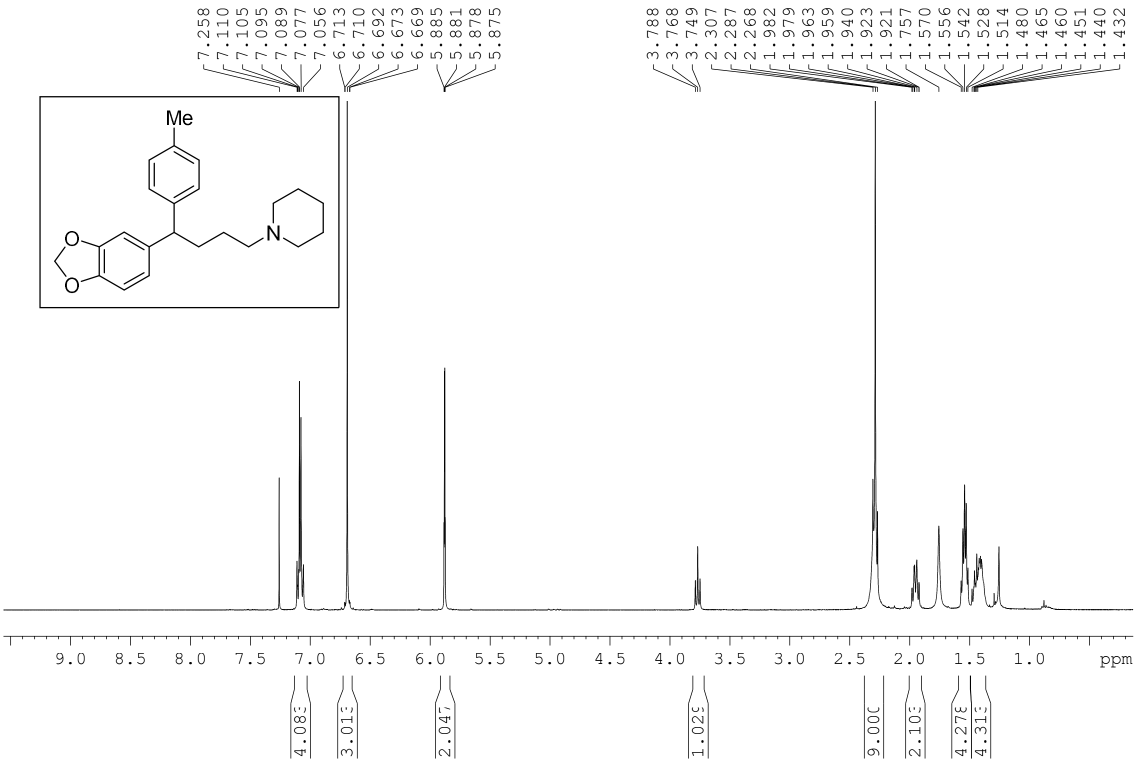

^{1}H NMR ($CDCl_3$, 400 MHz) spectrum of compound 8a

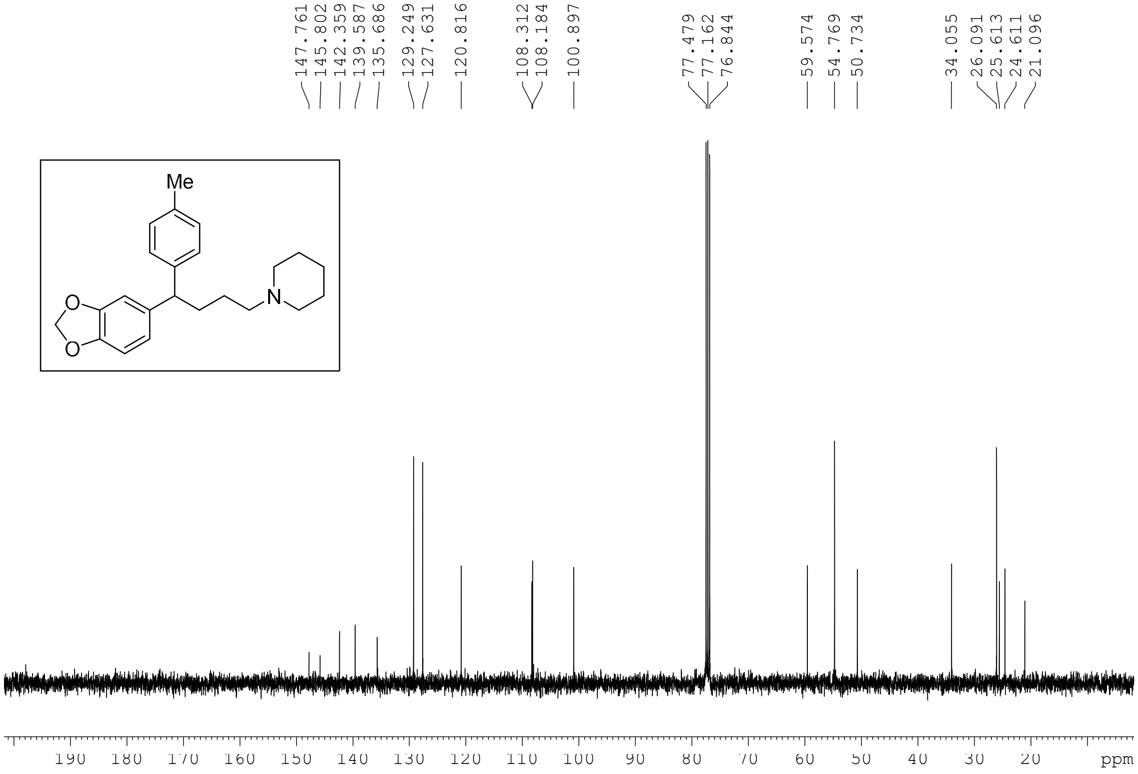

^{13}C NMR ($CDCl_3$, 100 MHz) spectrum of compound 8a

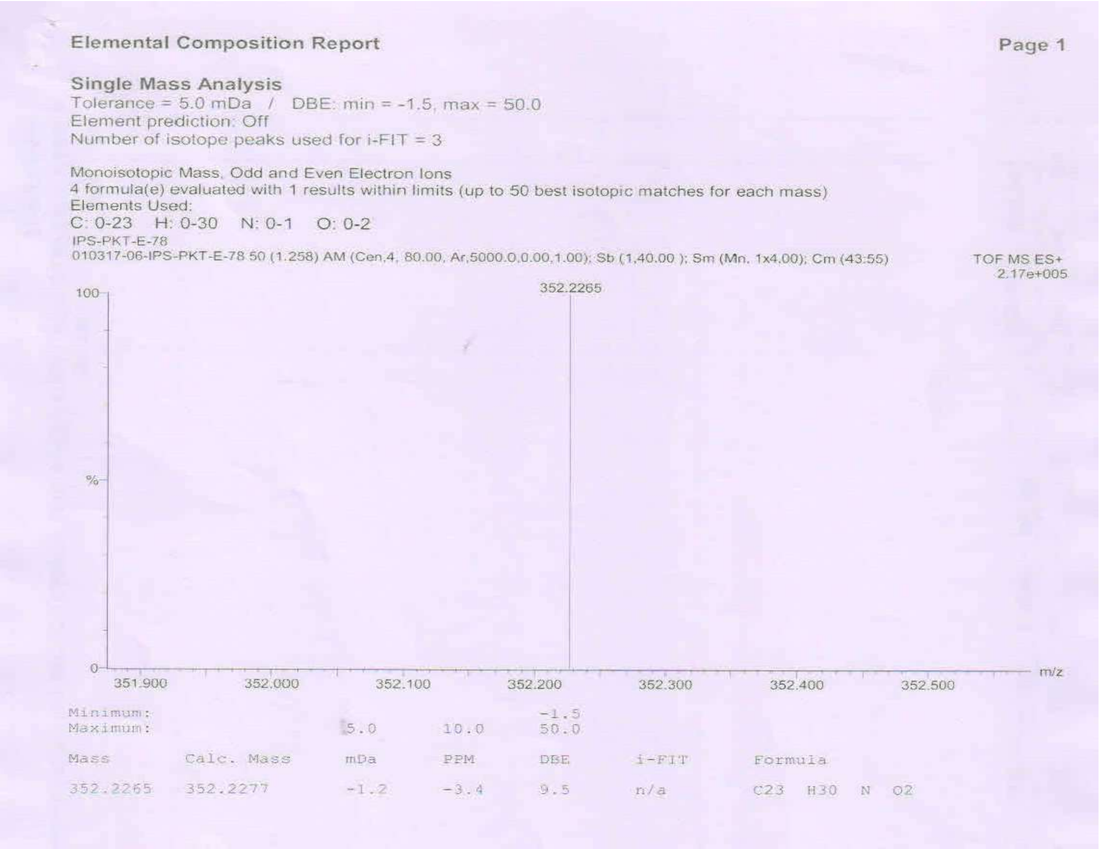

Elemental Composition Report

Page 1

Single Mass Analysis
Tolerance = 5.0 mDa / DBE: min = -1.5, max = 50.0
Element prediction: Off
Number of isotope peaks used for i-FIT = 3

Monoisotopic Mass, Odd and Even Electron Ions
4 formula(e) evaluated with 1 results within limits (up to 50 best isotopic matches for each mass)
Elements Used:
C: 0-23 H: 0-30 N: 0-1 O: 0-2
IPS-PKT-E-78
010317-06-IPS-PKT-E-78 50 (1.258) AM (Cen,4, 80.00, Ar,5000.0,0.00,1.00); Sb (1,40.00); Sm (Mn, 1x4.00); Cm (43:55)

TOF MS ES+
2.17e+005

Minimum:				-1.5		
Maximum:		5.0	10.0	50.0		
Mass	Calc. Mass	mDa	PPM	DBE	i-FIT	Formula
352.2265	352.2277	-1.2	-3.4	9.5	n/a	C23 H30 N O2

ESI Mass spectrum of compound 8a

Compound 8b:

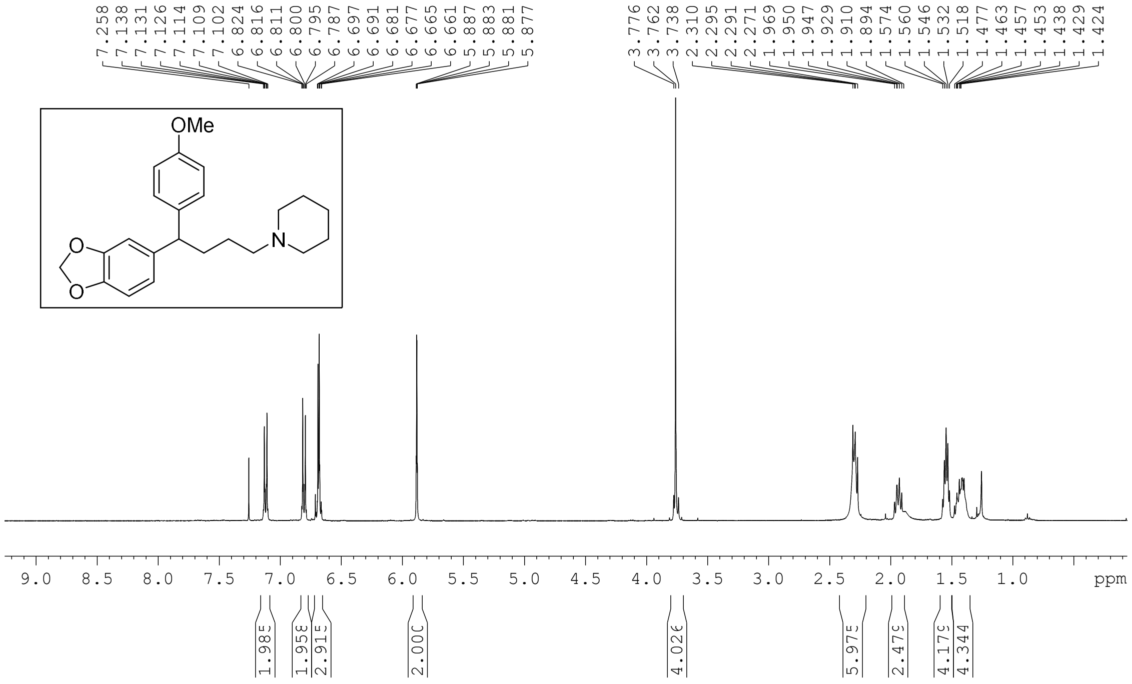

^{1}H NMR ($CDCl_3$, 400 MHz) spectrum of compound 8b

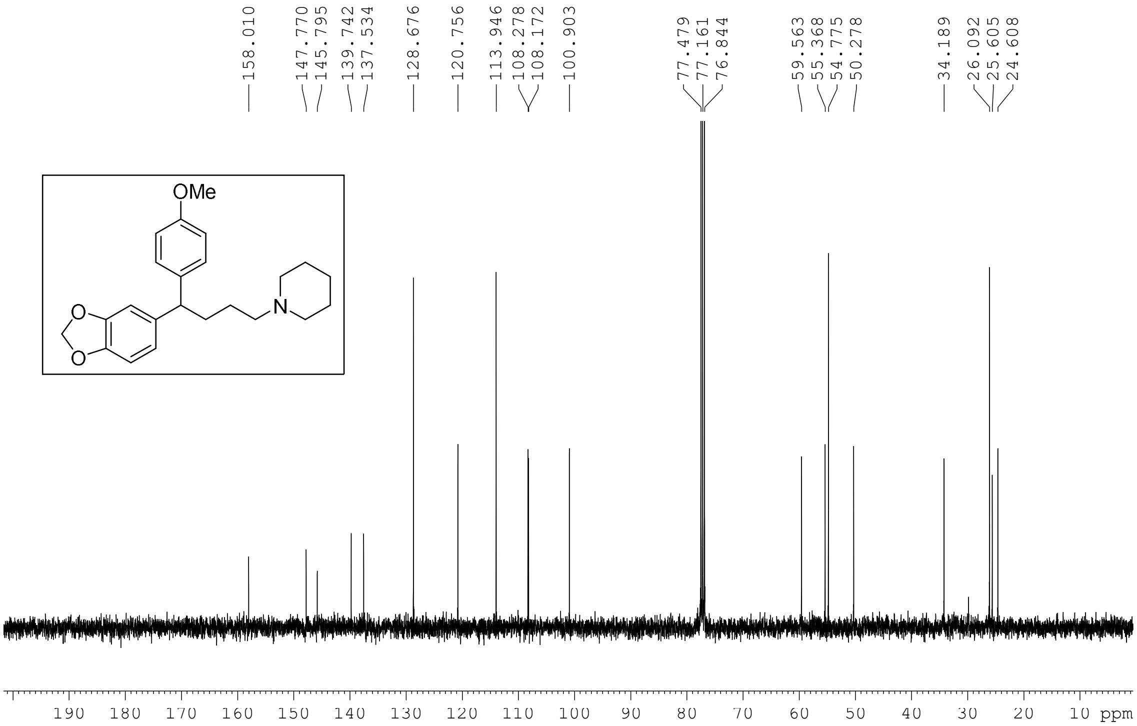

^{13}C NMR ($CDCl_3$, 100 MHz) spectrum of compound 8b

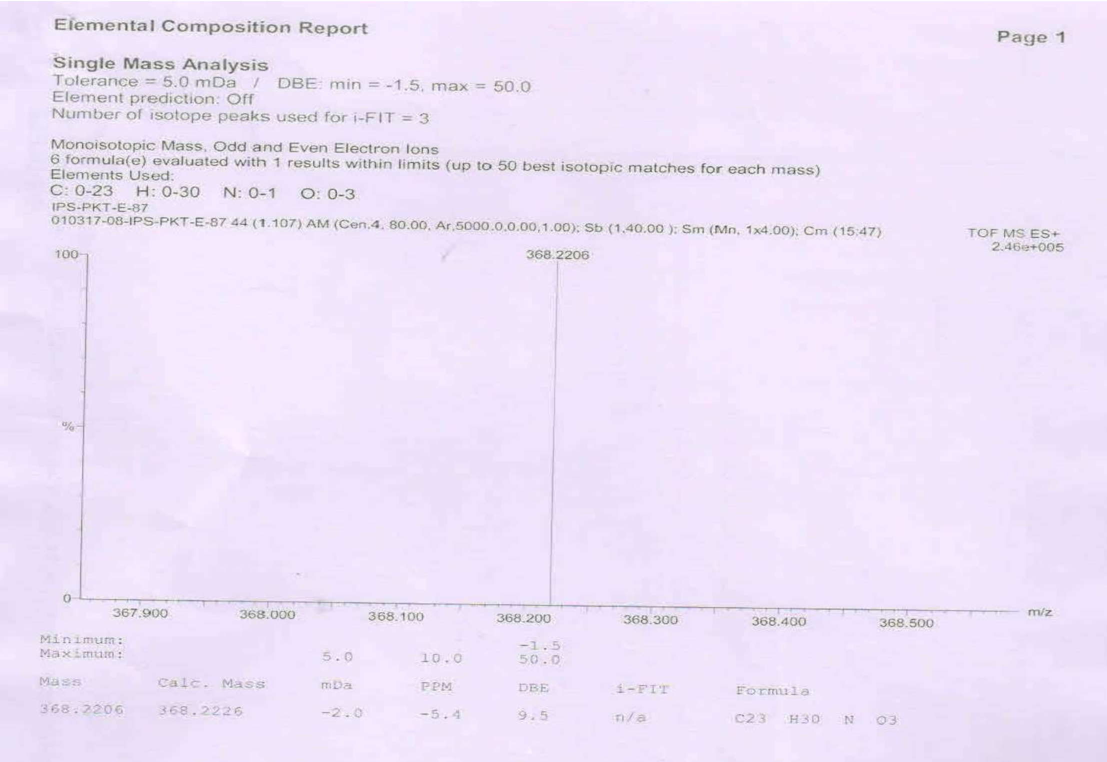

ESI Mass spectrum of compound 8b

Compound 8c:

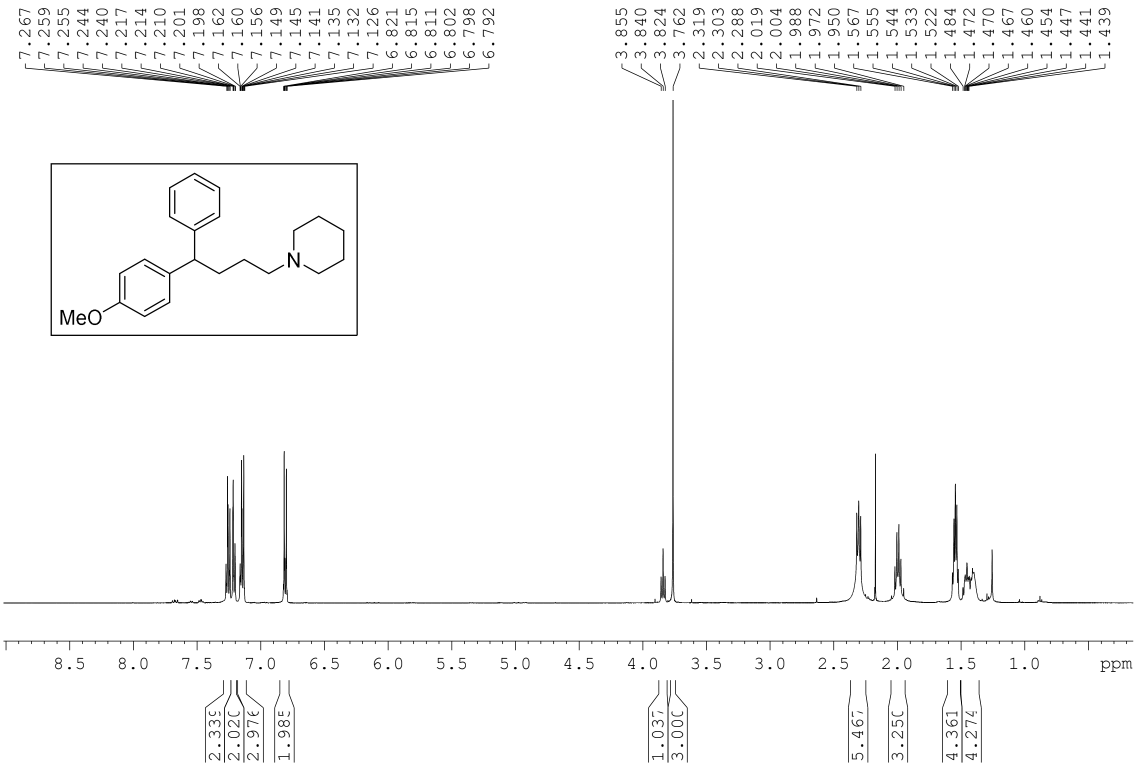

^{1}H NMR ($CDCl_3$, 500 MHz) spectrum of compound 8c

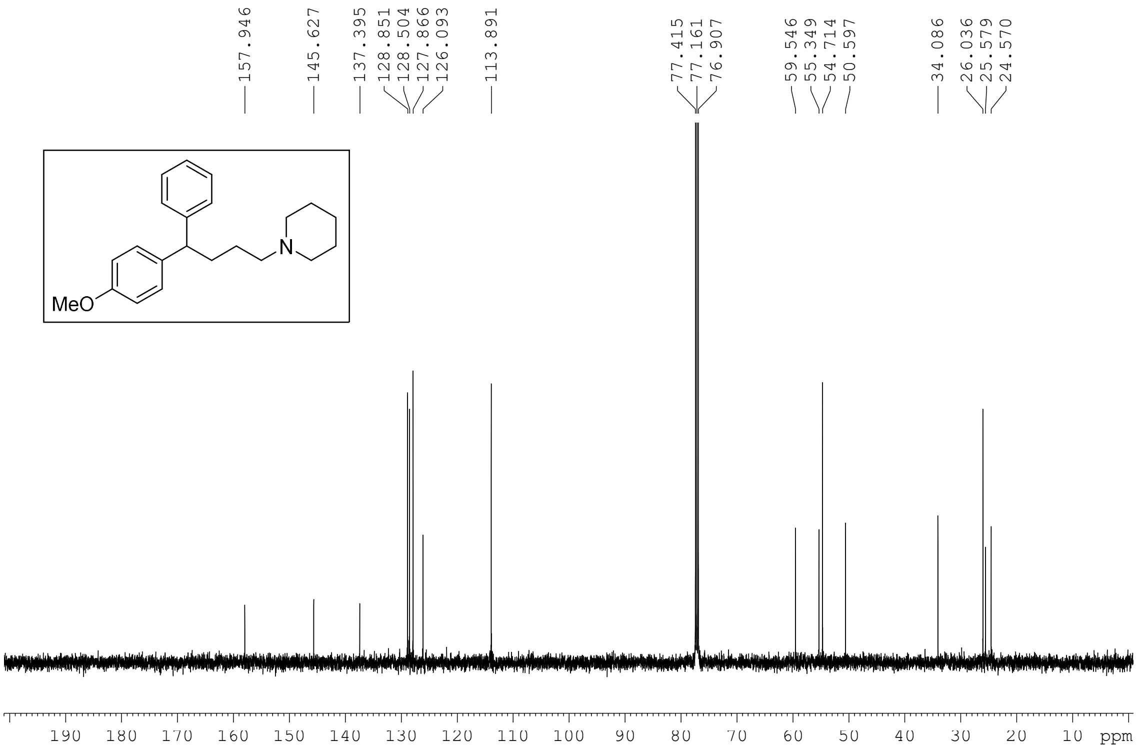

^{13}C NMR ($CDCl_3$, 125 MHz) spectrum of compound 8c

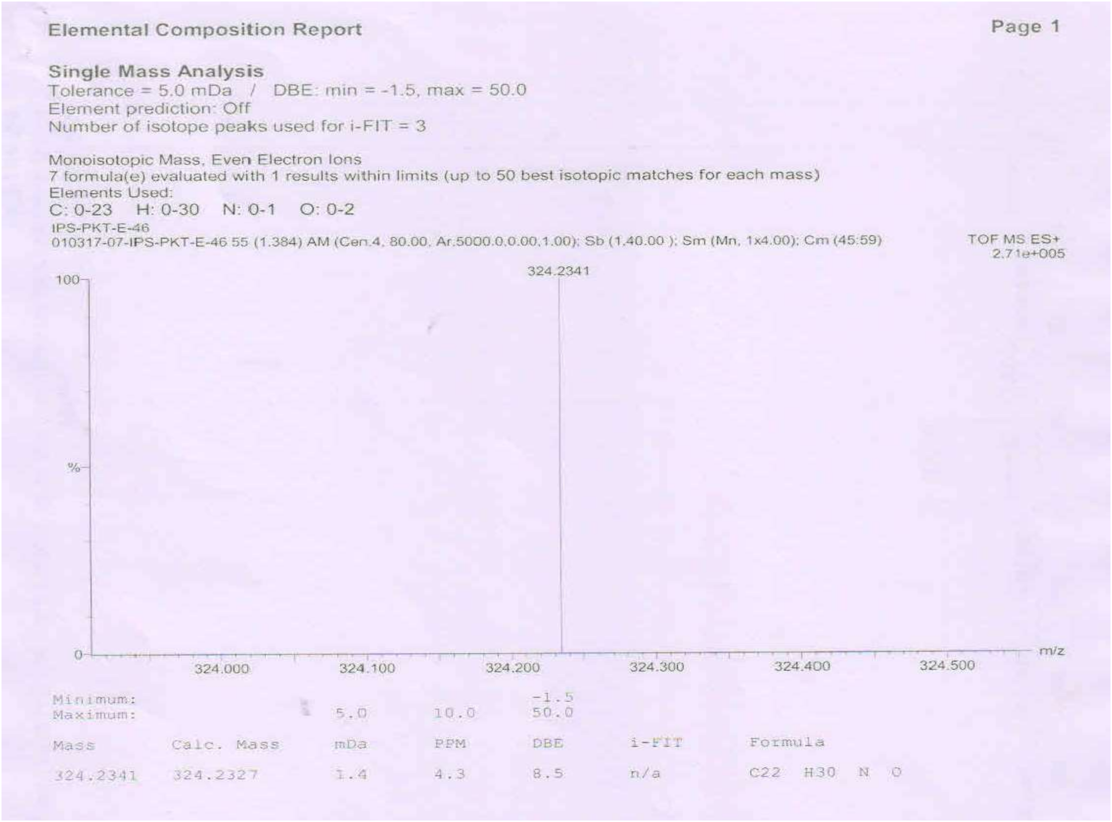

ESI Mass spectrum of compound 8c

Compound 8d:

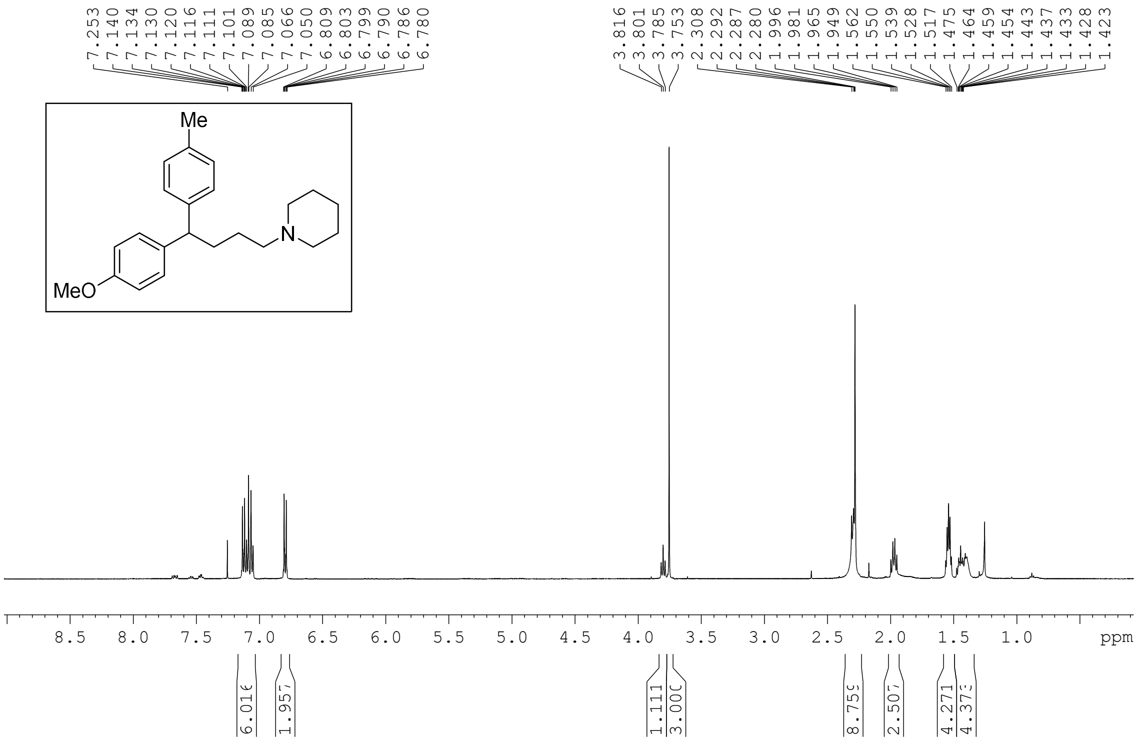

^{1}H NMR (CDCl3, 500 MHz) spectrum of compound 8d

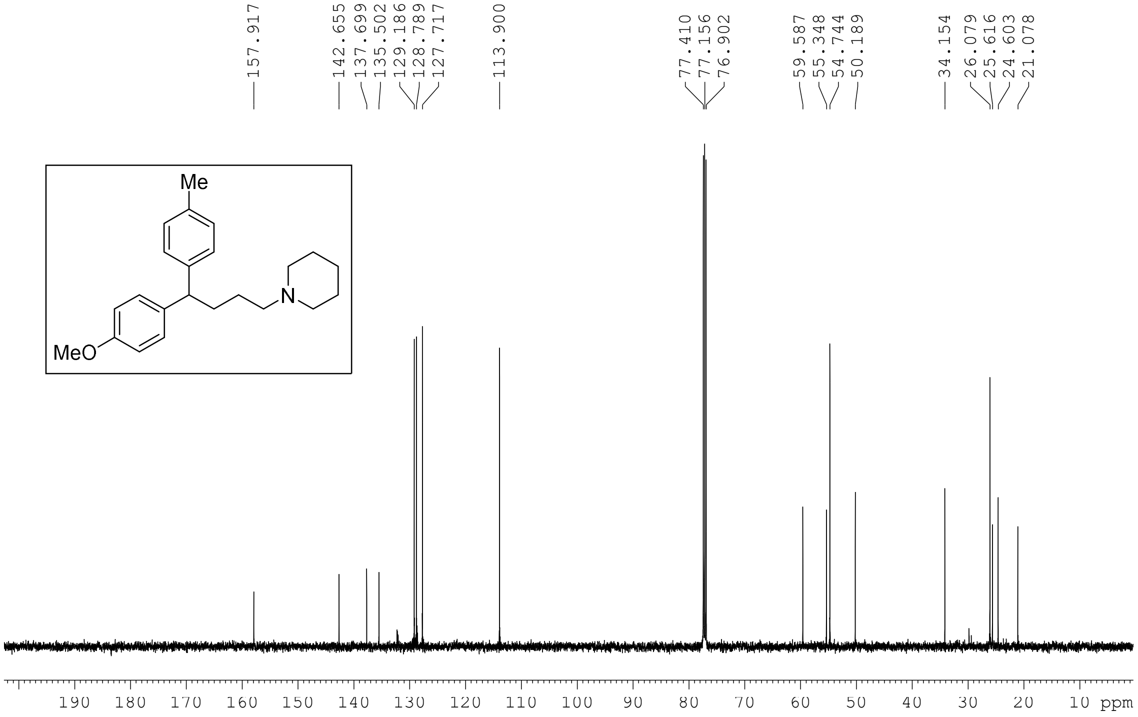

^{13}C NMR (CDCl3, 125 MHz) spectrum of compound 8d

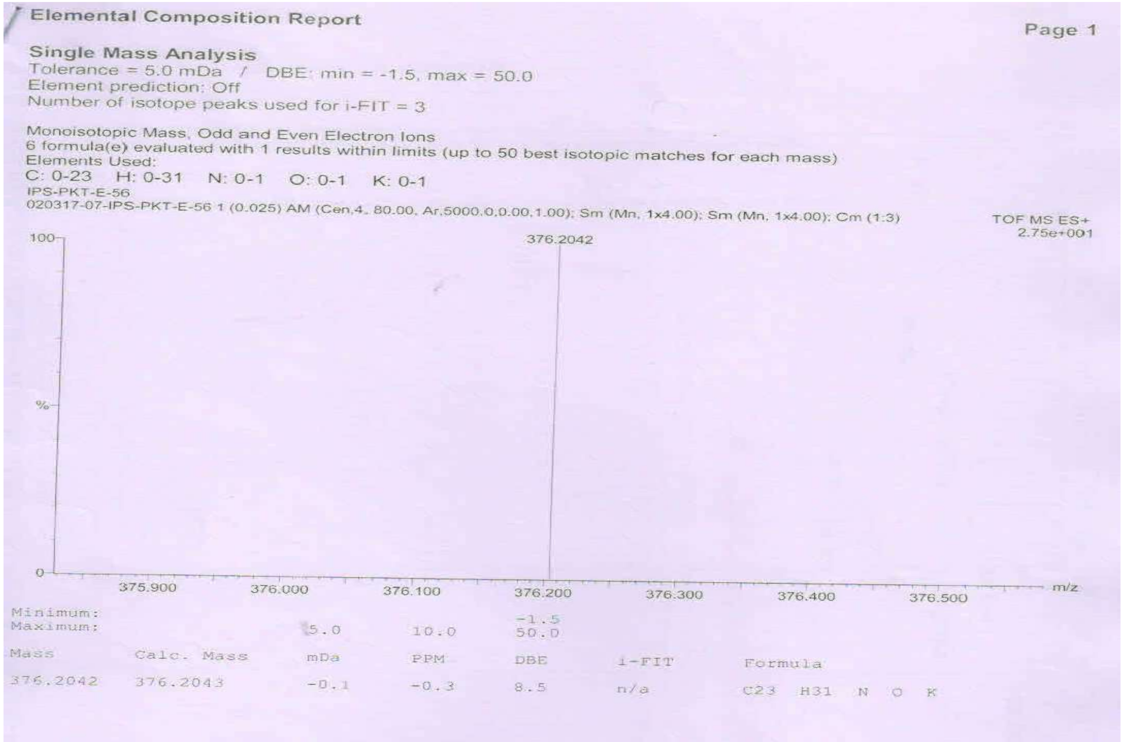

ESI Mass spectrum of compound 8d

Compound 8e:

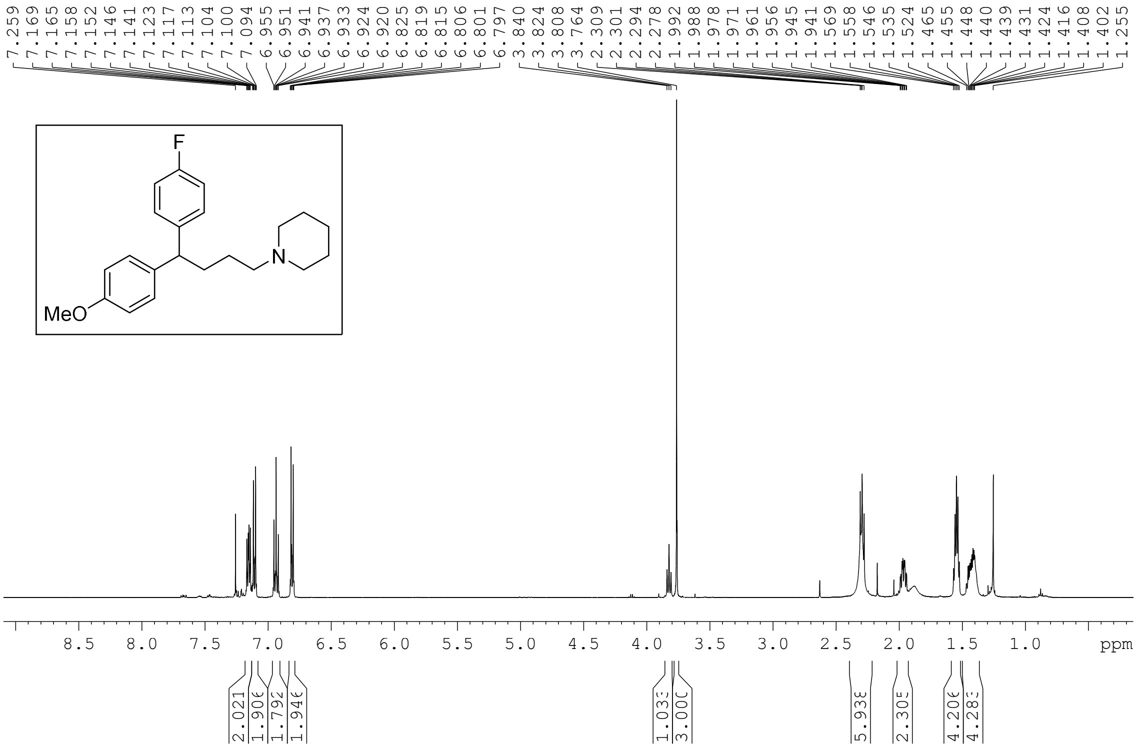

^{1}H NMR ($CDCl_3$, 500 MHz) spectrum of compound 8e

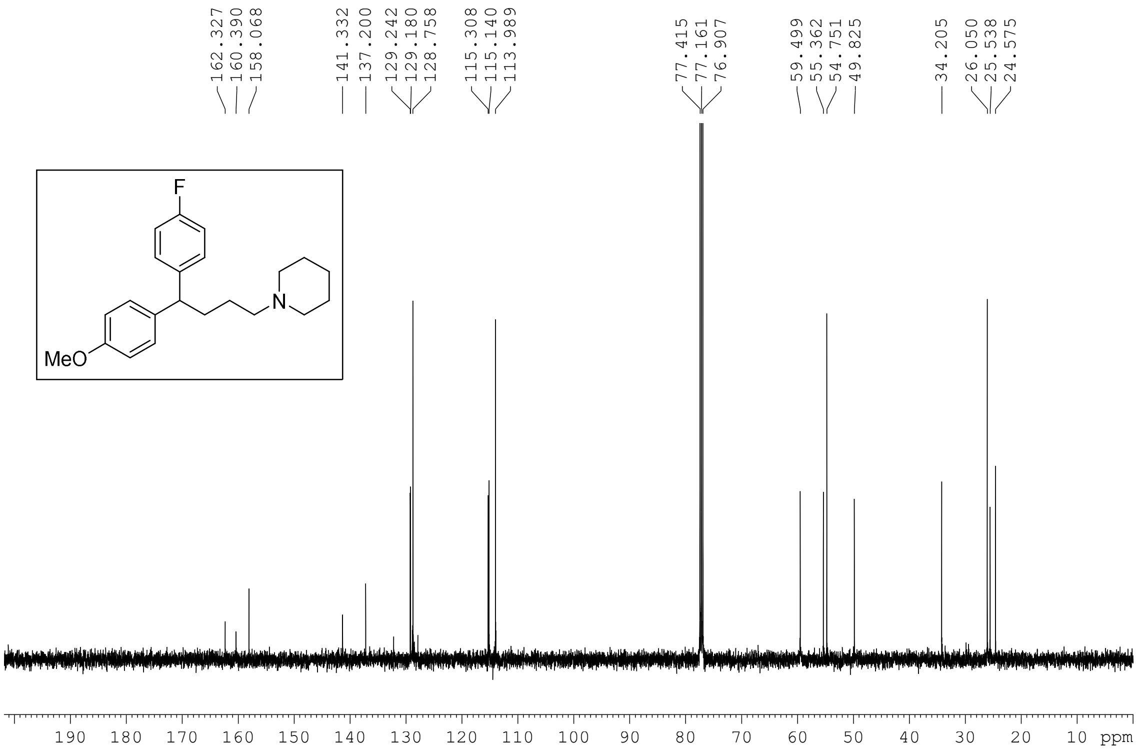

^{13}C NMR ($CDCl_3$, 125 MHz) spectrum of compound 8e

Compound 1a:

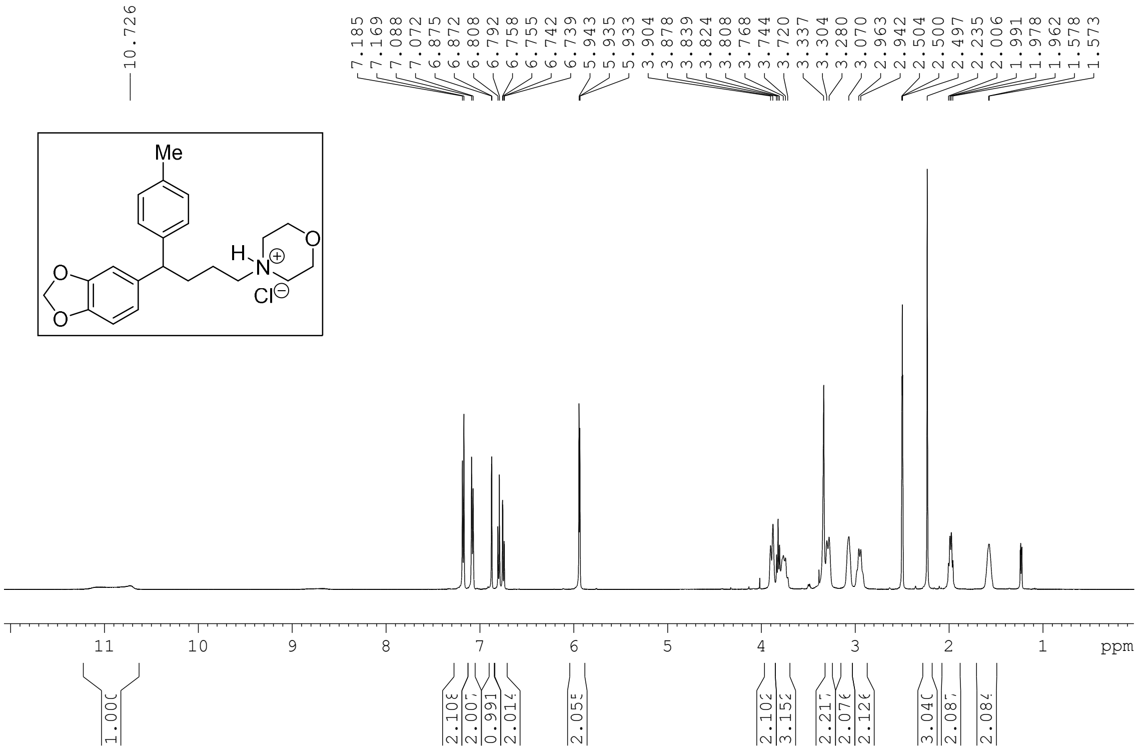

^{1}H NMR (DMSO-d_6, 500 MHz) spectrum of compound 1a

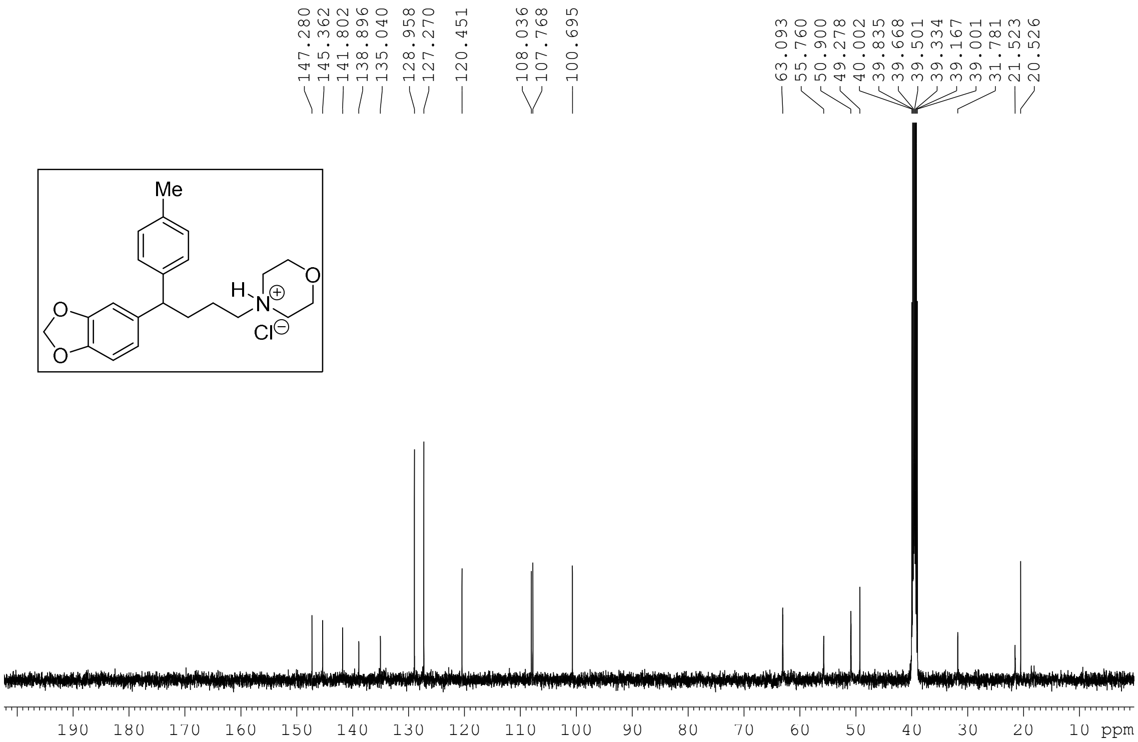

^{13}C NMR (DMSO-d_6, 125 MHz) spectrum of compound 1a

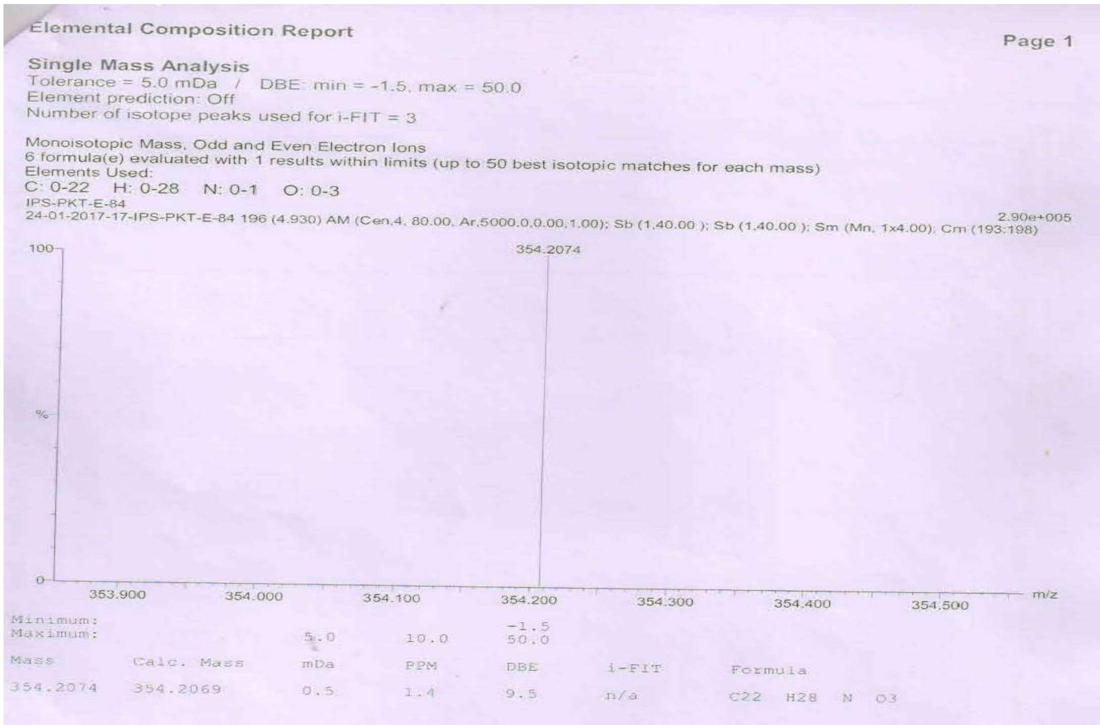

ESI Mass spectrum of compound 5a

Compound 1b:

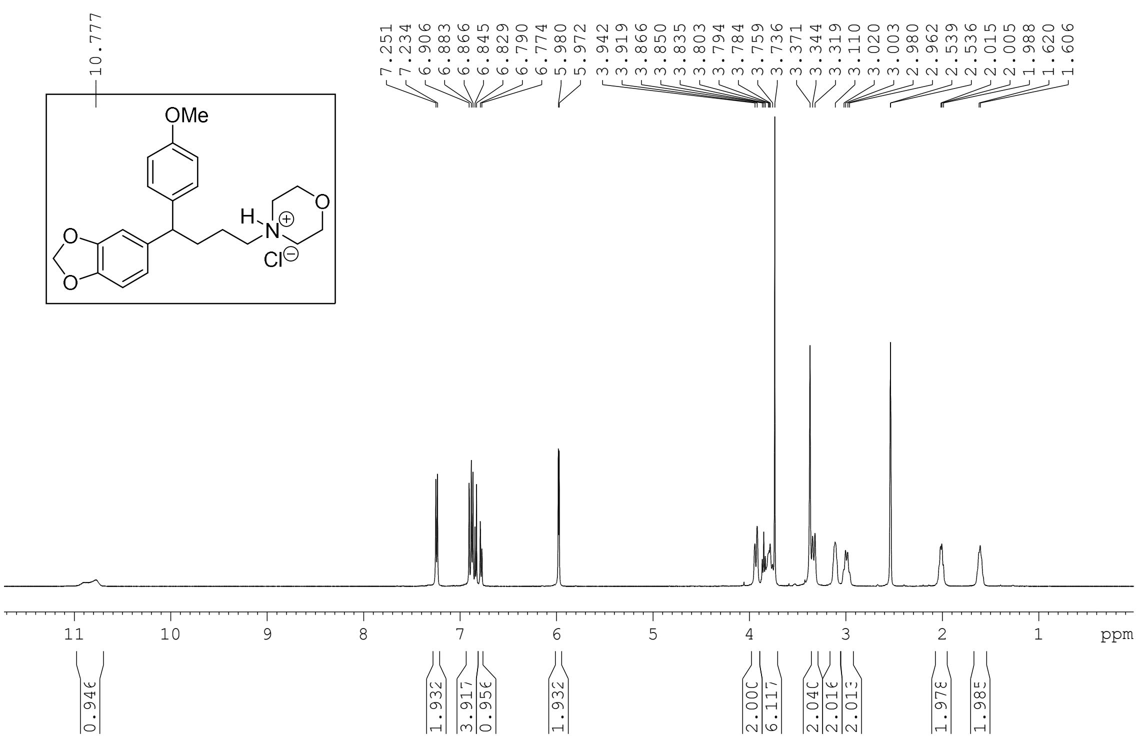

^{1}H NMR (DMSO-d_6, 500 MHz) spectrum of compound 1b

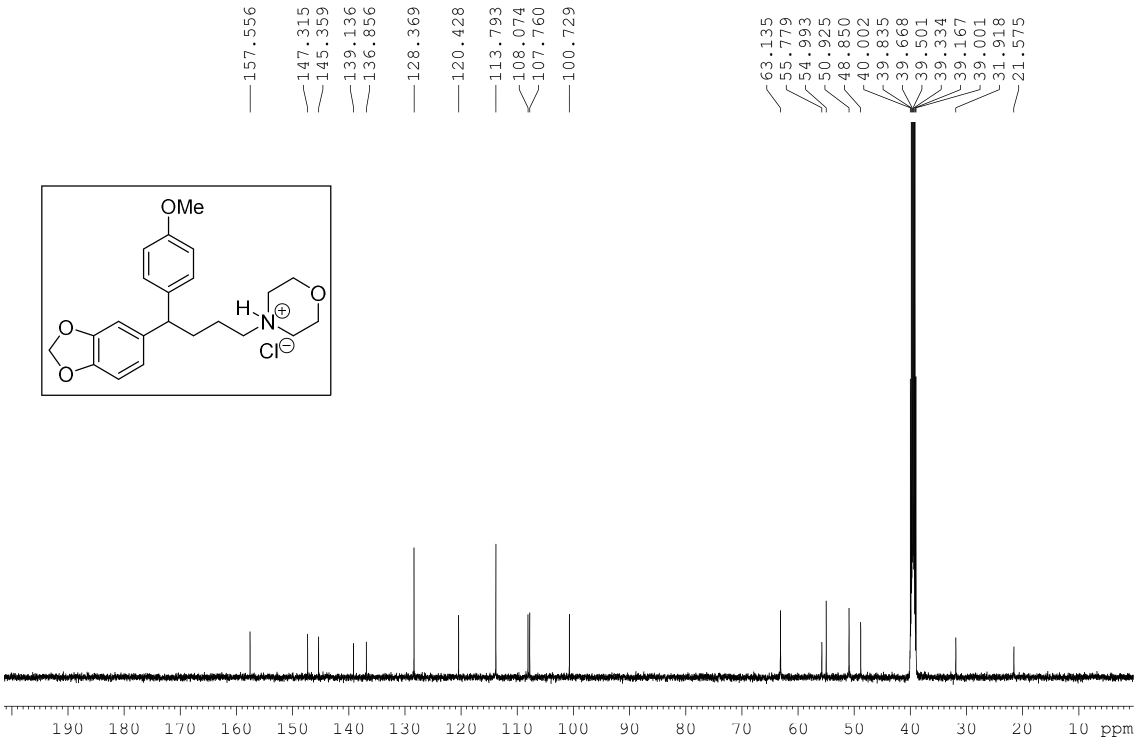

^{13}C NMR (DMSO-d_6, 125 MHz) spectrum of compound 1b

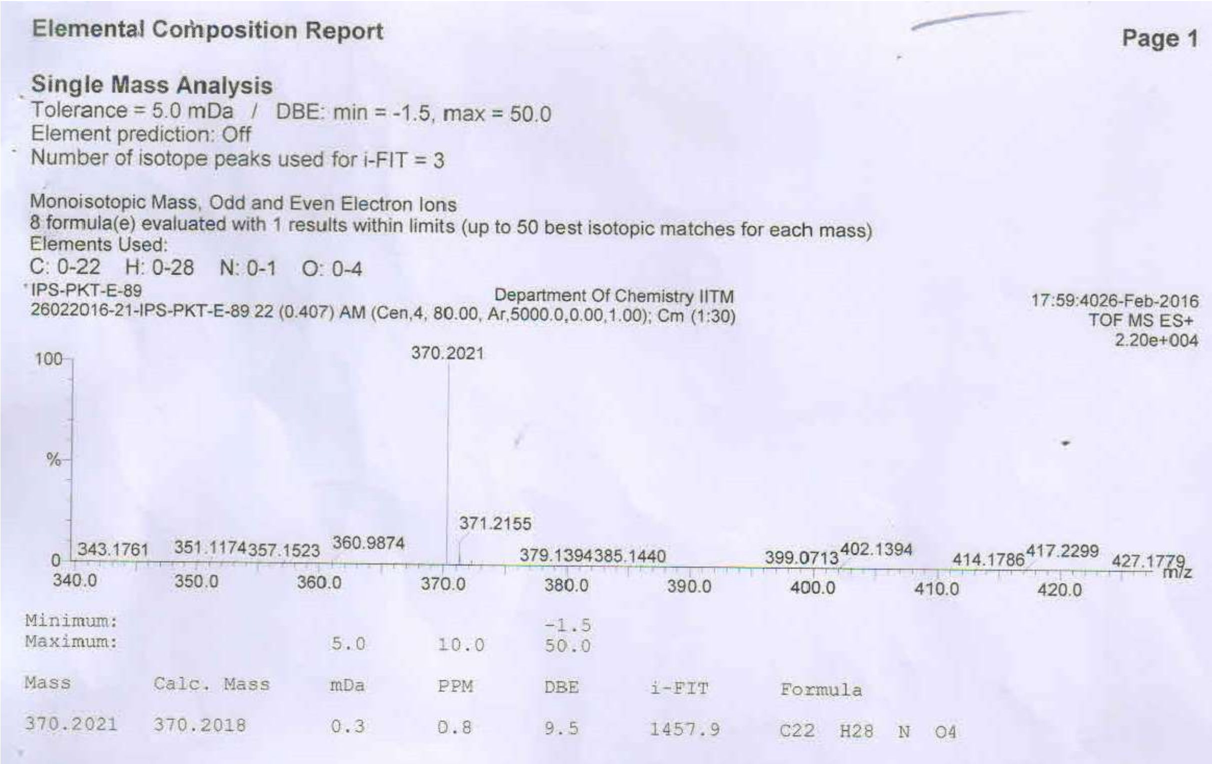

Elemental Composition Report

Page 1

Single Mass Analysis
Tolerance = 5.0 mDa / DBE: min = -1.5, max = 50.0
Element prediction: Off
Number of isotope peaks used for i-FIT = 3

Monoisotopic Mass, Odd and Even Electron Ions
8 formula(e) evaluated with 1 results within limits (up to 50 best isotopic matches for each mass)
Elements Used:
C: 0-22 H: 0-28 N: 0-1 O: 0-4
IPS-PKT-E-89 Department Of Chemistry IITM
26022016-21-IPS-PKT-E-89 22 (0.407) AM (Cen,4, 80.00, Ar,5000.0,0.00,1.00); Cm (1:30)
17:59:4026-Feb-2016
TOF MS ES+
2.20e+004

Minimum:				-1.5		
Maximum:		5.0	10.0	50.0		
Mass	Calc. Mass	mDa	PPM	DBE	i-FIT	Formula
370.2021	370.2018	0.3	0.8	9.5	1457.9	C22 H28 N O4

ESI Mass spectrum of compound 1b

Compound 1c:

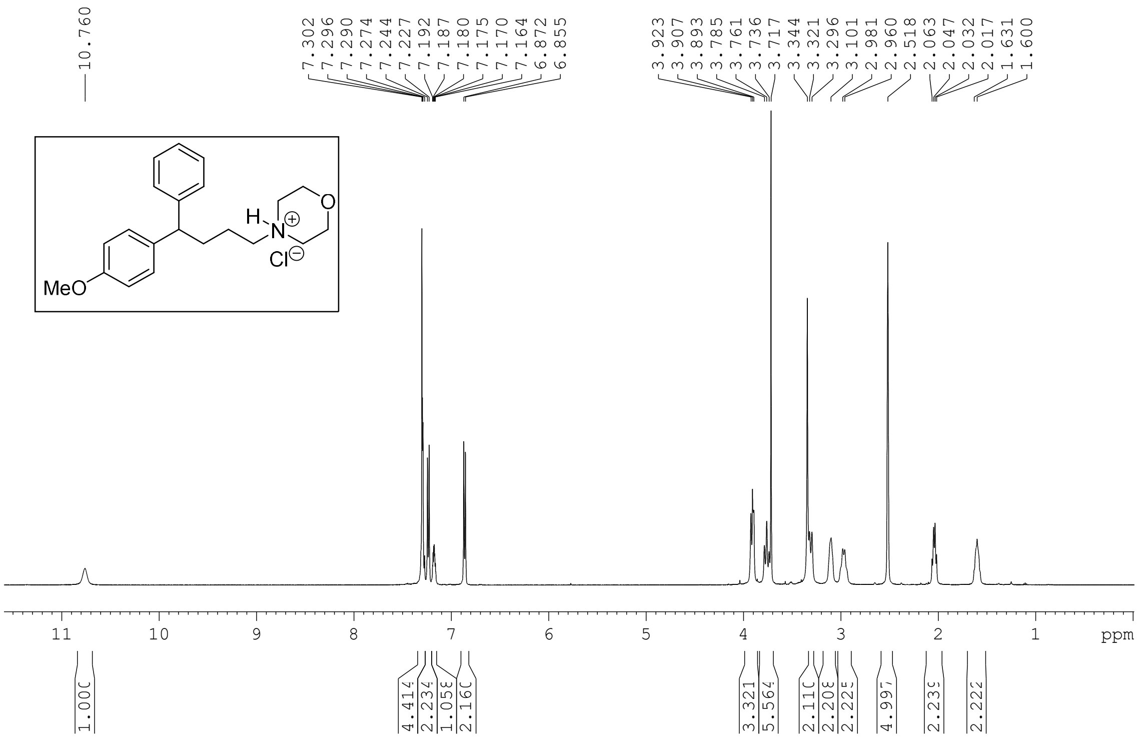

^{1}H NMR (DMSO-d_6, 500 MHz) spectrum of compound 1c

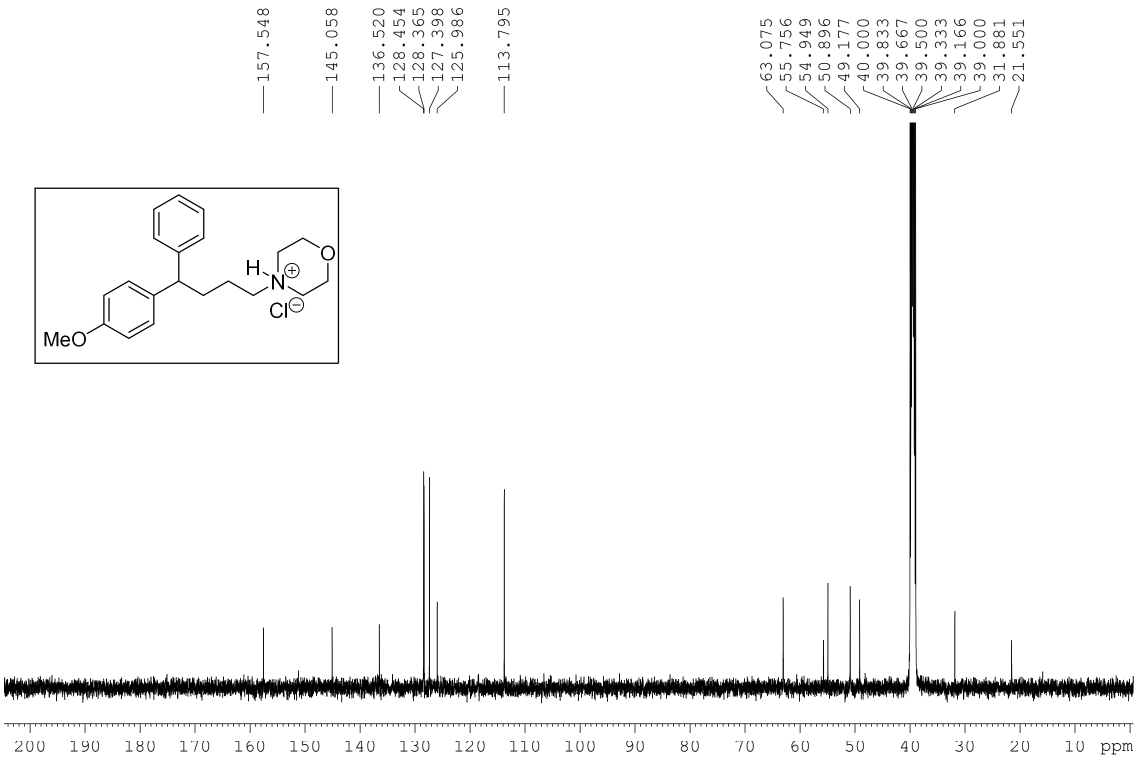

^{13}C NMR (DMSO-d_6, 125 MHz) spectrum of compound 1c

HRMS DST-FIST Funded, Department of Chemistry, IIT Madras

ESI Mass Report

**Name**	230719-30-IPS-PKT-E-47	**Data File Path**	D:\MassHunter\Data\2019\JULY-2019\IPS-PKT-E-47.d
**Sample ID**		**Acq. Time (Local)**	23-07-2019 19:14:52 (UTC+05:30)
**Instrument**	Instrument 1	**Method Path (Acq)**	D:\MassHunter\Methods\Direct Infusion_HPLC.m
**MS Type**	QTOF	**Version (Acq SW)**	6200 series TOF/6500 series Q-TOF B.08.00 (B8058.0)
**Inj. Vol. (ul)**	5	**IRM Status**	Success
**Position**	P1-C8	**Method Path (DA)**	D:\MassHunter\Methods\10.0\IIT-Target Screening_1.m
**Plate Pos.**		**Target Source Path**	
**Operator**		**Result Summary**	1 qualified (1 targets)

Compound Details

Cpd. 1: C21 H27 N O2

Compound Spectra (overlaid)

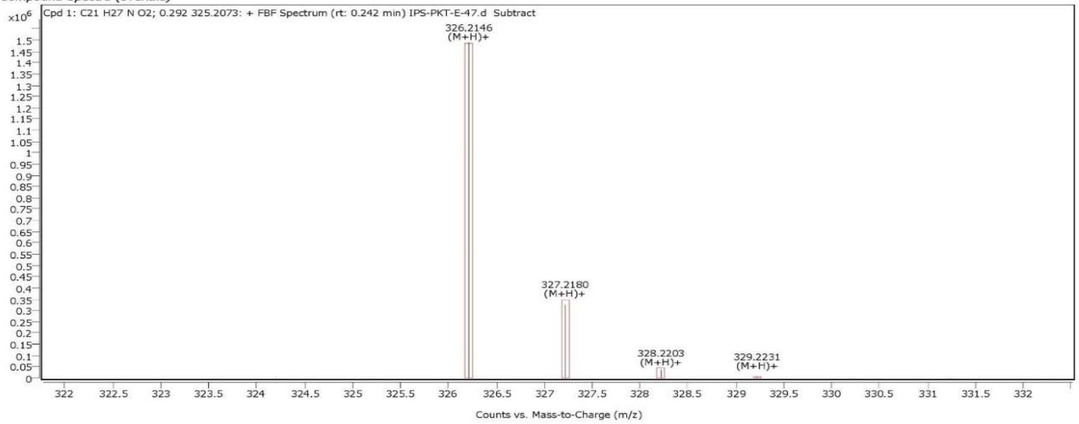

Compound ID Table

Cpd	Formula	Mass (Tgt)	Calc. Mass	Mass	Species	Diff(Tgt.ppm)	mDa
1	C21 H27 N O2	325.2042	325.2073	326.2146	(M+H)+	9.60	3.12

ESI Mass spectrum of compound 1c

Compound 1d:

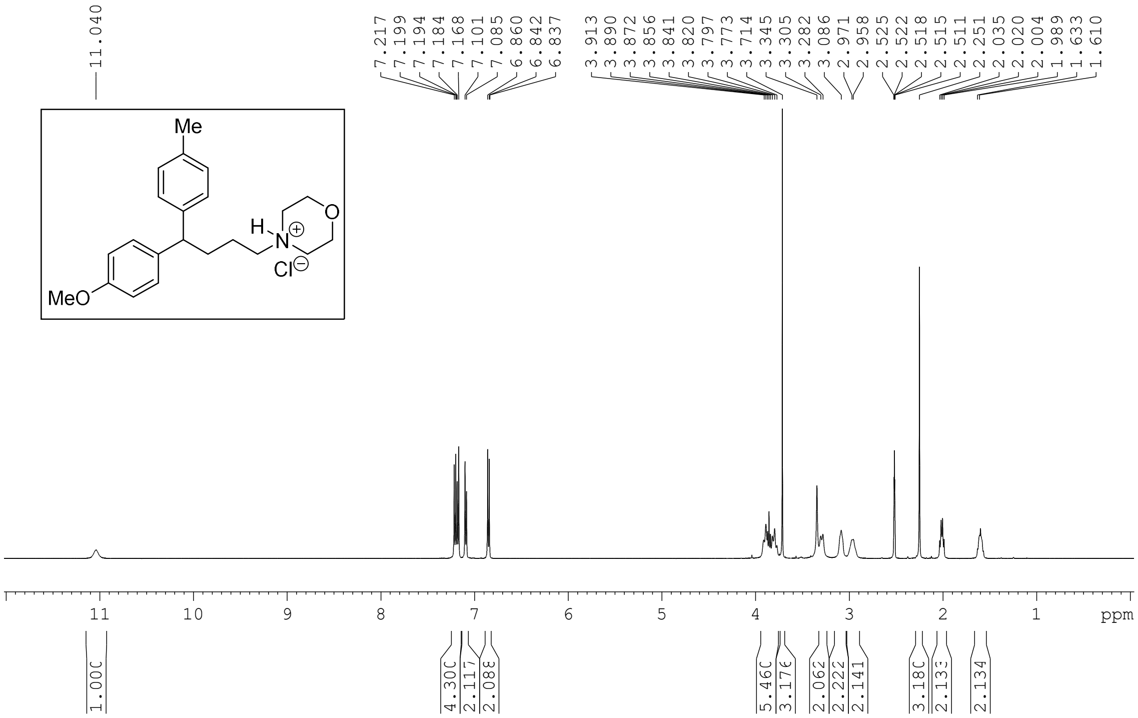

^{1}H NMR (DMSO-d_6, 500 MHz) spectrum of compound 1d

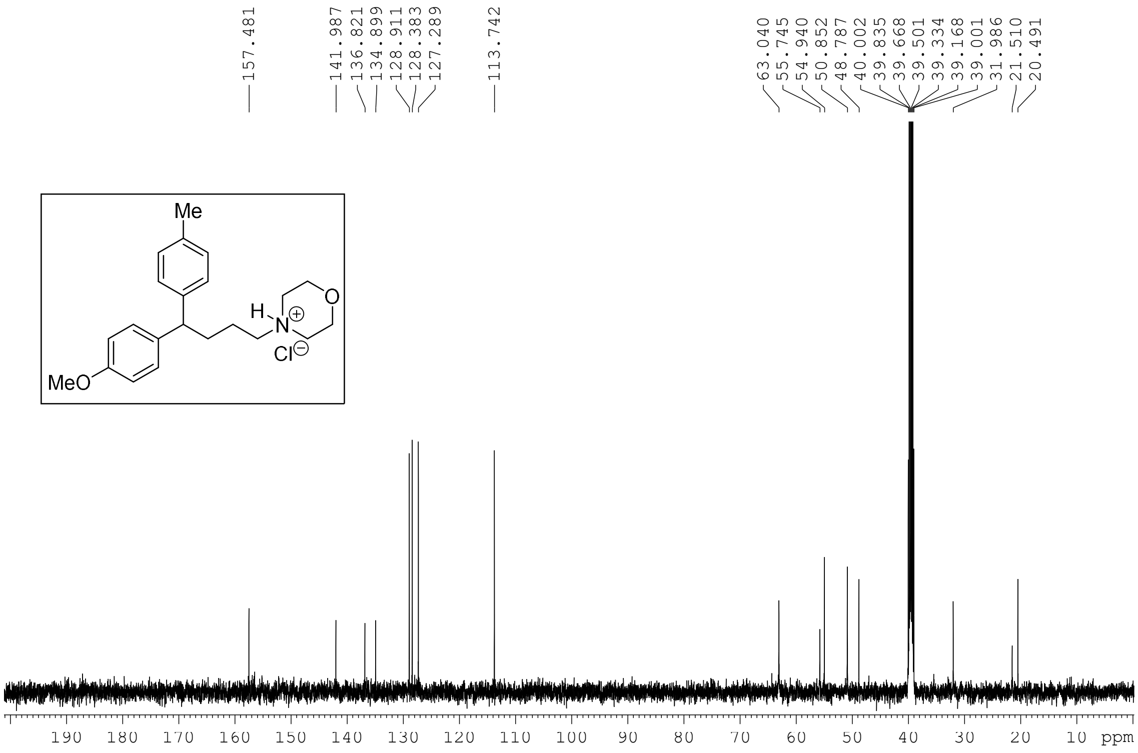

^{13}C NMR (DMSO-d_6, 125 MHz) spectrum of compound 1d

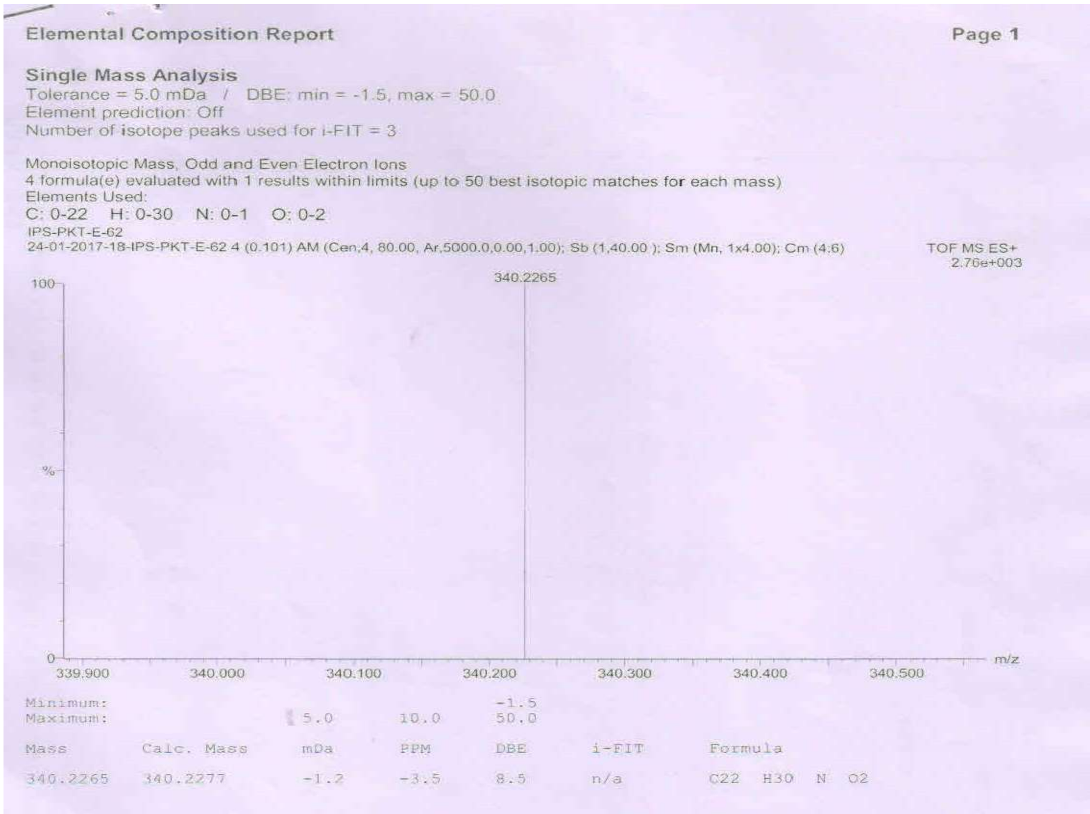

ESI Mass spectrum of compound 1c

Compound 1e:

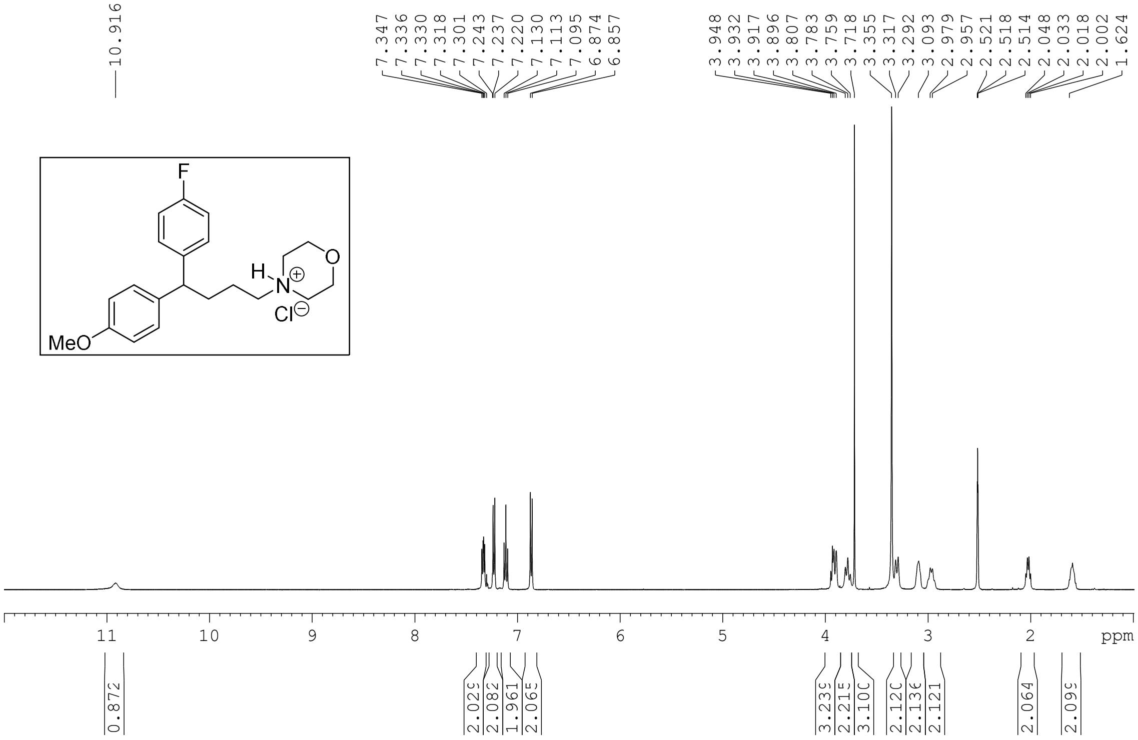

^{1}H NMR (DMSO-d_6, 500 MHz) spectrum of compound 1e

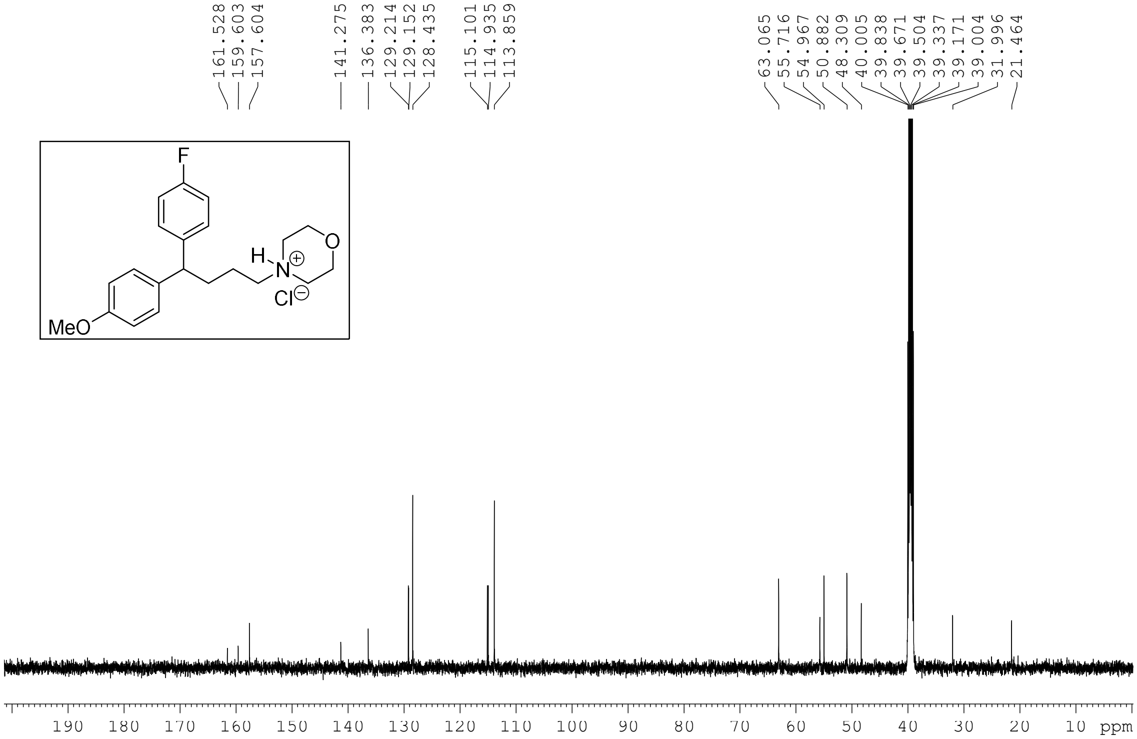

^{13}C NMR (DMSO-d_6, 125 MHz) spectrum of compound 1e

HRMS DST-FIST Funded, Department of Chemistry, IIT Madras

ESI Mass Report

**Name**	230719-28-IPS-PKT-E-68	**Data File Path**	D:\MassHunter\Data\2019\JULY-2019\IPS-PKT-E-68.d
**Sample ID**		**Acq. Time (Local)**	23-07-2019 17:59:45 (UTC+05:30)
**Instrument**	Instrument 1	**Method Path (Acq)**	D:\MassHunter\Methods\Direct Infusion_HPLC.m
**MS Type**	QTOF	**Version (Acq SW)**	6200 series TOF/6500 series Q-TOF B.08.00 (B8058.0)
**Inj. Vol. (ul)**	5	**IRM Status**	Success
**Position**	P1-C6	**Method Path (DA)**	D:\MassHunter\Methods\10.0\IIT-Target Screening_1.m
**Plate Pos.**		**Target Source Path**	
**Operator**		**Result Summary**	1 qualified (1 targets)

Compound Details

Cpd. 1: C21 H26 F N O2

Compound Spectra (overlaid)

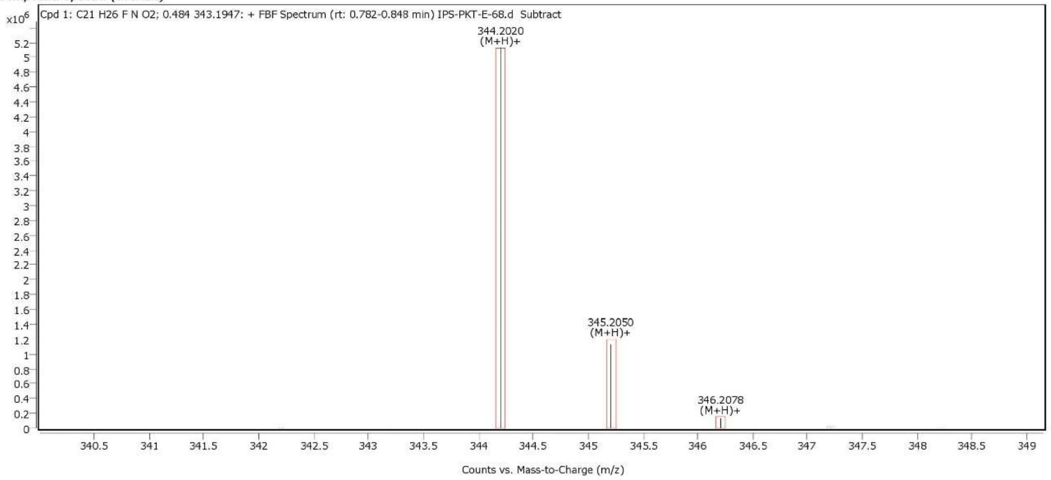

Compound ID Table

Cpd	Formula	Mass (Tgt)	Calc. Mass	Mass	Species	Diff(Tgt.ppm)	mDa
1	C21 H26 F N O2	343.1948	343.1947	344.2020	(M+H)+	-0.20	-0.07

ESI Mass spectrum of compound 1e

Compound 2a:

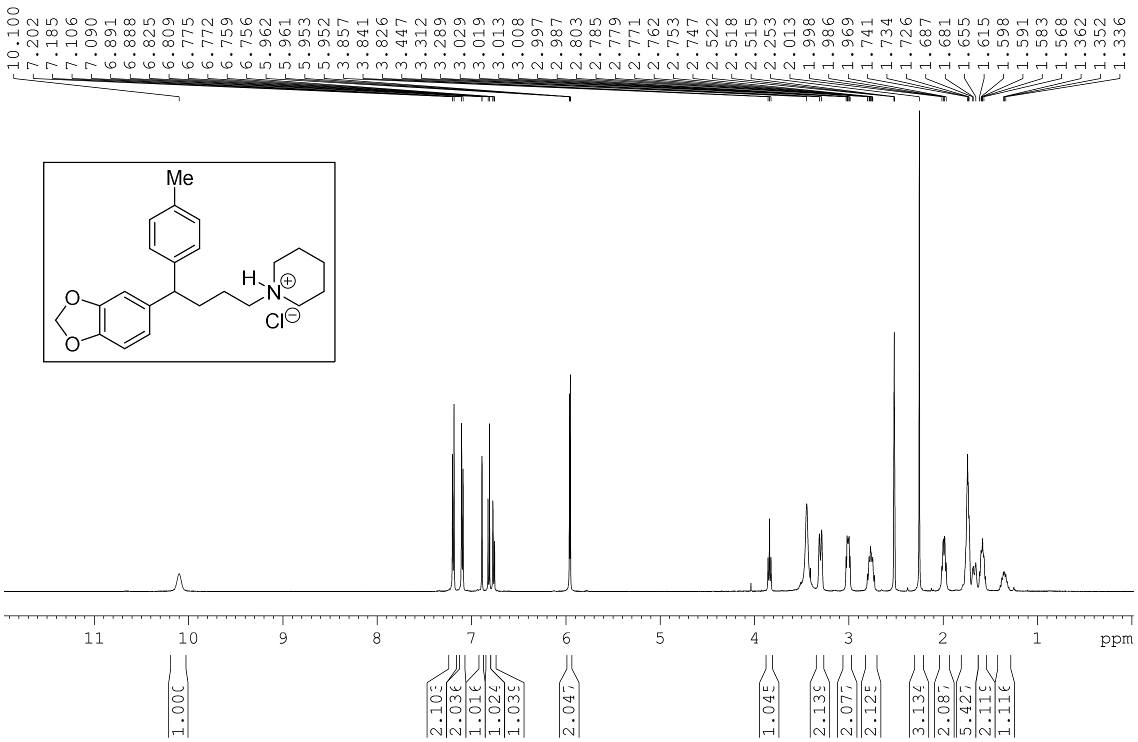

^{1}H NMR (DMSO-d_6, 500 MHz) spectrum of compound 2a

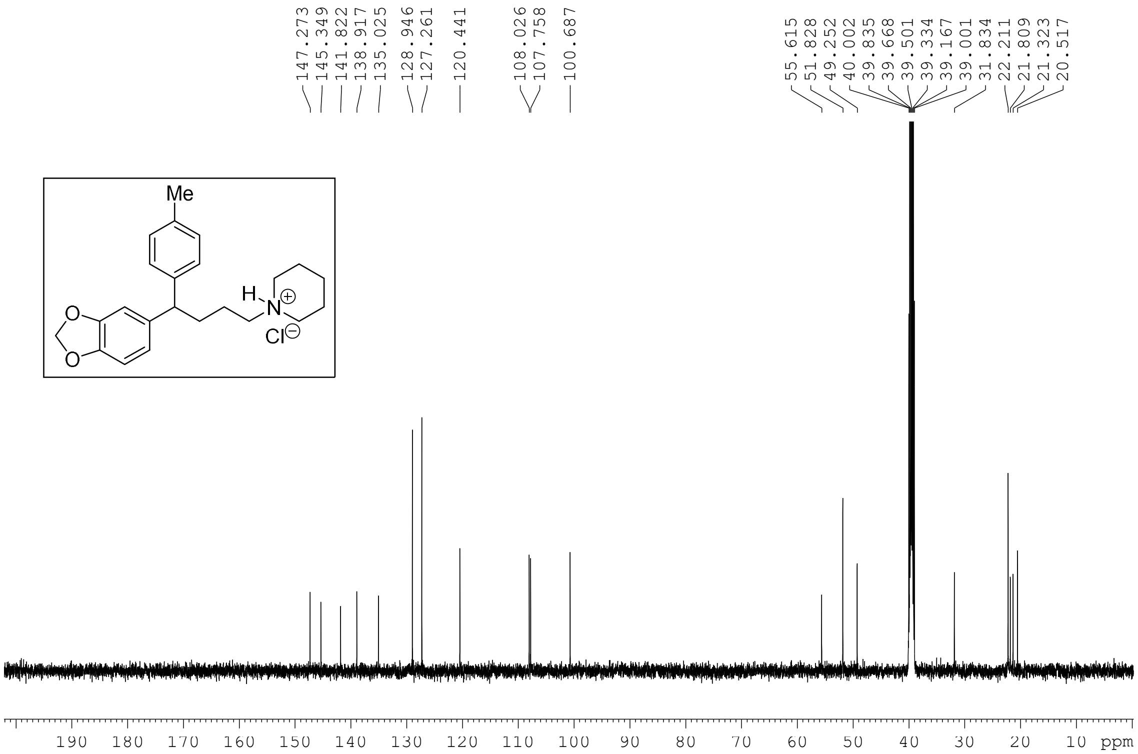

^{13}C NMR (DMSO-d_6, 125 MHz) spectrum of compound 2a

Compound 2b:

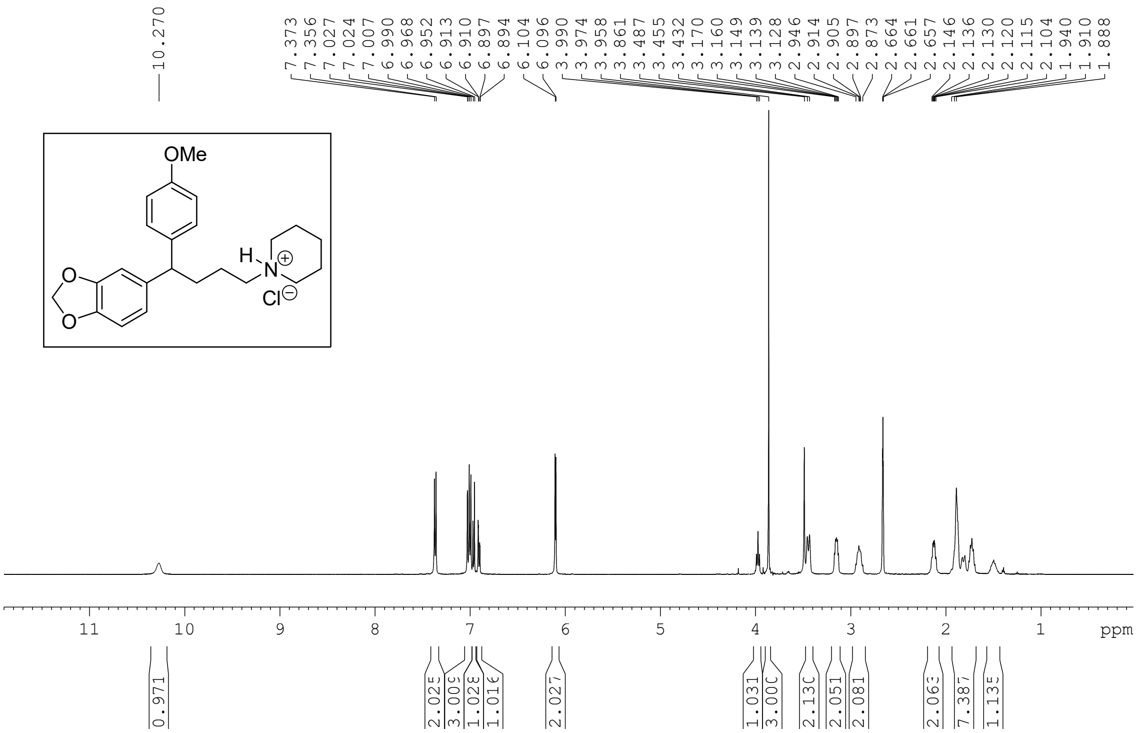

^{1}H NMR (DMSO-d_6, 500 MHz) spectrum of compound 2b

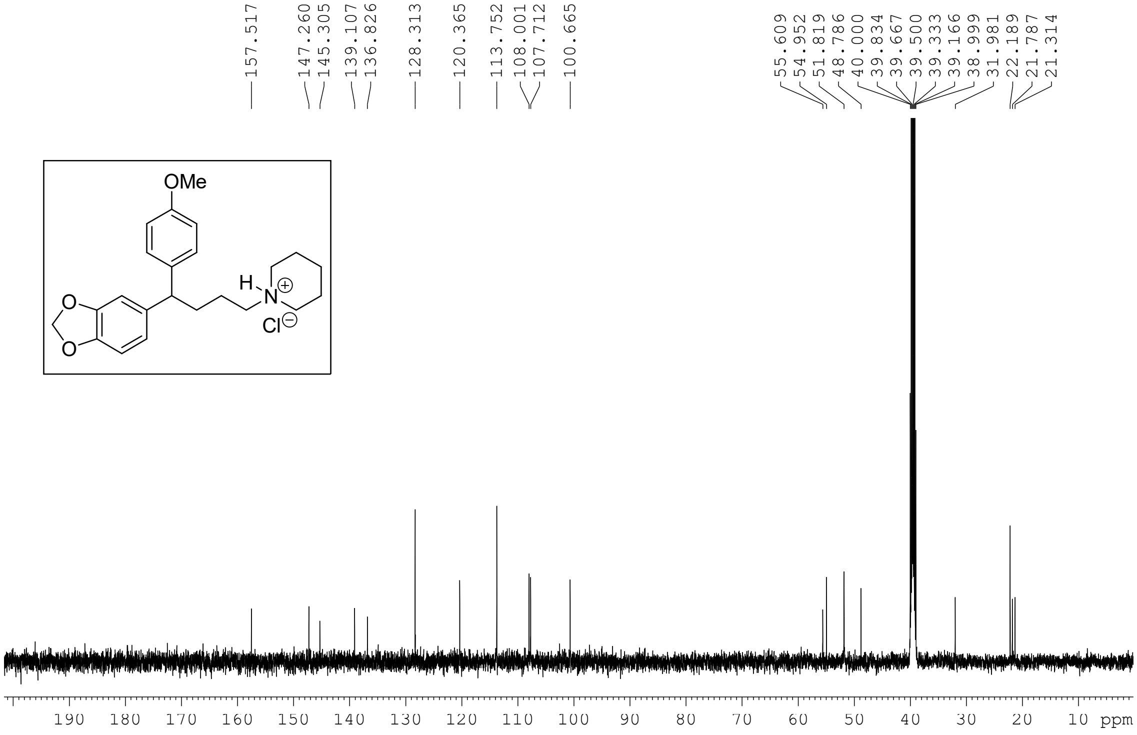

^{13}C NMR (DMSO-d_6, 125 MHz) spectrum of compound 2b

HRMS DST-FIST Funded, Department of Chemistry, IIT Madras

ESI Mass Report

**Name**	250719-22-IPS-PKT-2b	**Data File Path**	D:\MassHunter\Data\2019\JULY-2019\IPS\PKT-2b.d
**Sample ID**		**Acq. Time (Local)**	25-07-2019 10:24:31 (UTC+05:30)
**Instrument**	Instrument 1	**Method Path (Acq)**	D:\MassHunter\Methods\Direct Infusion_HPLC.m
**MS Type**	QTOF	**Version (Acq SW)**	6200 series TOF/6500 series Q-TOF B.08.00 (B8058.0)
**Inj. Vol. (ul)**	5	**IRM Status**	Success
**Position**	P1-F1	**Method Path (DA)**	D:\MassHunter\Methods\10.0\IIT-Target Screening_1.m
**Plate Pos.**		**Target Source Path**	
**Operator**		**Result Summary**	1 qualified (1 targets)

Compound Details

Cpd. 1: C23 H29 N O3

Compound Spectra (overlaid)

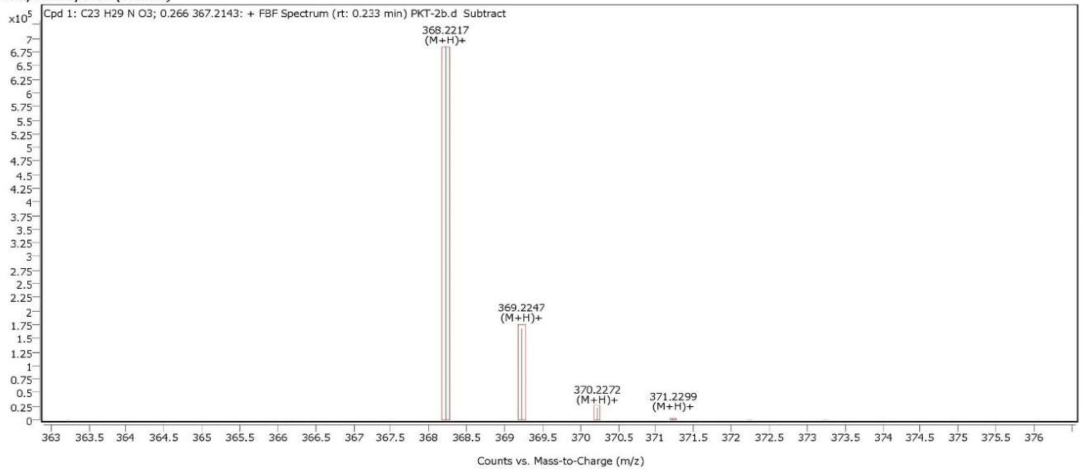

Compound ID Table

Cpd	Formula	Mass (Tgt)	Calc. Mass	Mass	Species	Diff(Tgt.ppm)	mDa
1	C23 H29 N O3	367.2147	367.2143	368.2217	(M+H)+	-1.09	-0.40

ESI Mass spectrum of compound 2b

Compound 2c:

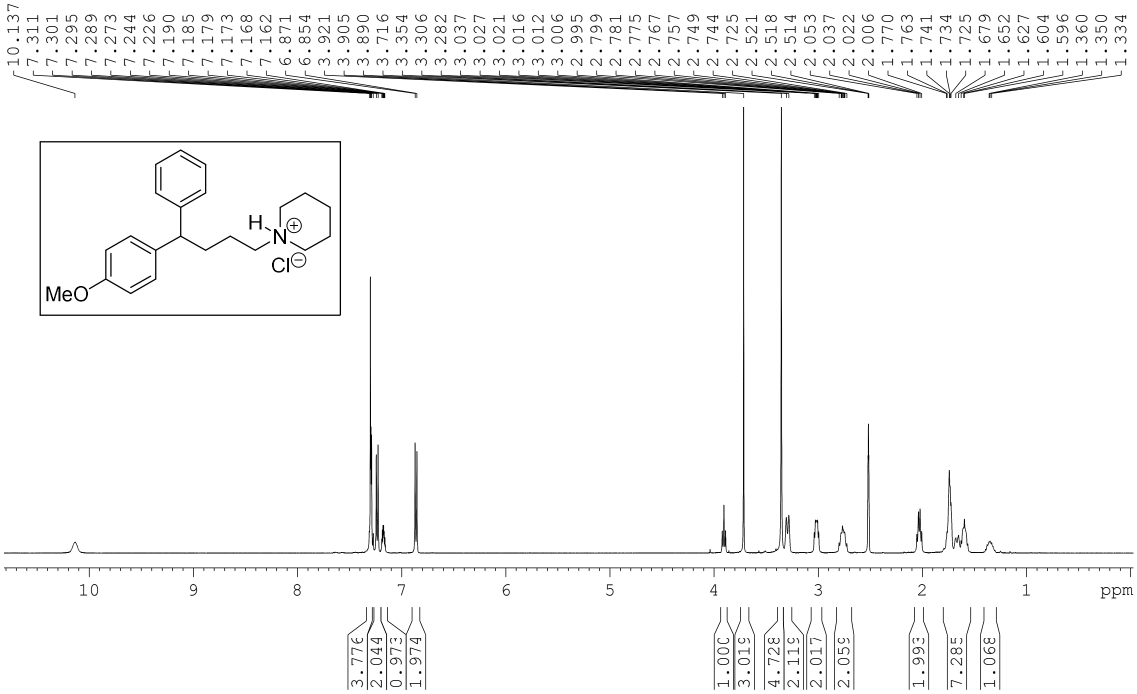

^{1}H NMR (DMSO-d_6, 500 MHz) spectrum of compound 2c

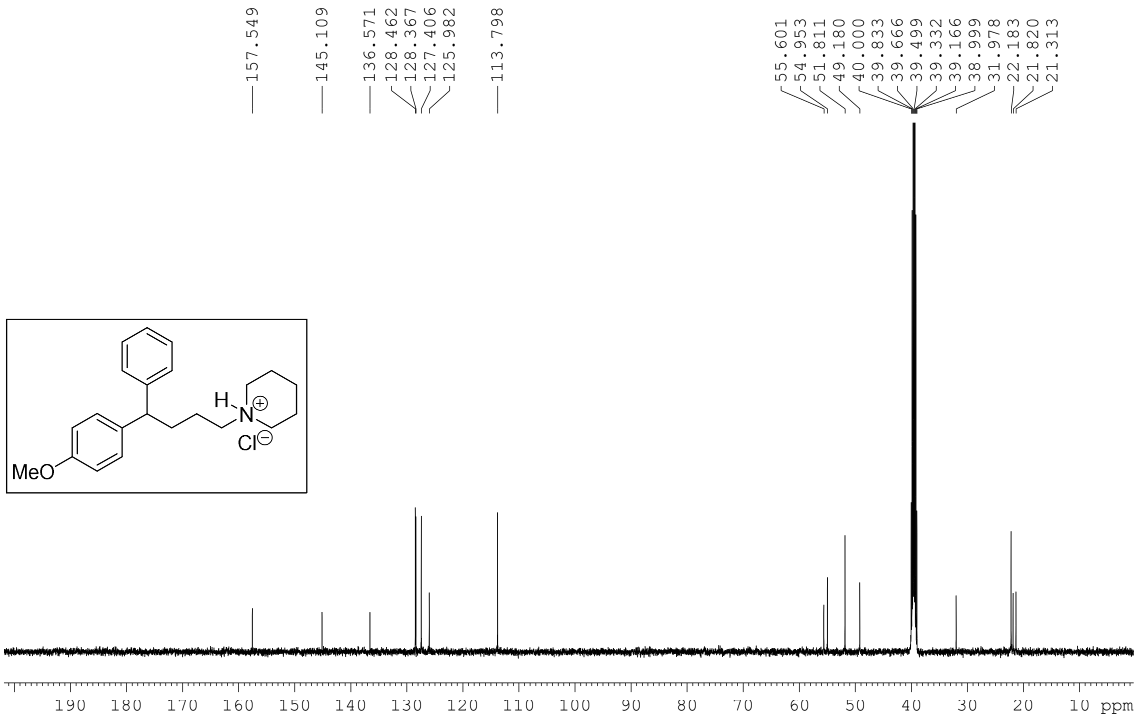

^{13}C NMR (DMSO-d_6, 125 MHz) spectrum of compound 2c

HRMS DST-FIST Funded, Department of Chemistry, IIT Madras

ESI Mass Report

**Name**	230719-29-IPS-PKT-E-53	**Data File Path**	D:\MassHunter\Data\2019\JULY-2019\IPS-PKT-E-53-2.d
**Sample ID**		**Acq. Time (Local)**	24-07-2019 08:53:24 (UTC+05:30)
**Instrument**	Instrument 1	**Method Path (Acq)**	D:\MassHunter\Methods\Direct Infusion_HPLC.m
**MS Type**	QTOF	**Version (Acq SW)**	6200 series TOF/6500 series Q-TOF B.08.00 (B8058.0)
**Inj. Vol. (ul)**	5	**IRM Status**	Success
**Position**	P1-C9	**Method Path (DA)**	D:\MassHunter\Methods\10.0\IIT-Target Screening_1.m
**Plate Pos.**		**Target Source Path**	
**Operator**		**Result Summary**	1 qualified (1 targets)

Compound Details

Cpd. 1: C22 H29 N O

Compound Spectra (overlaid)

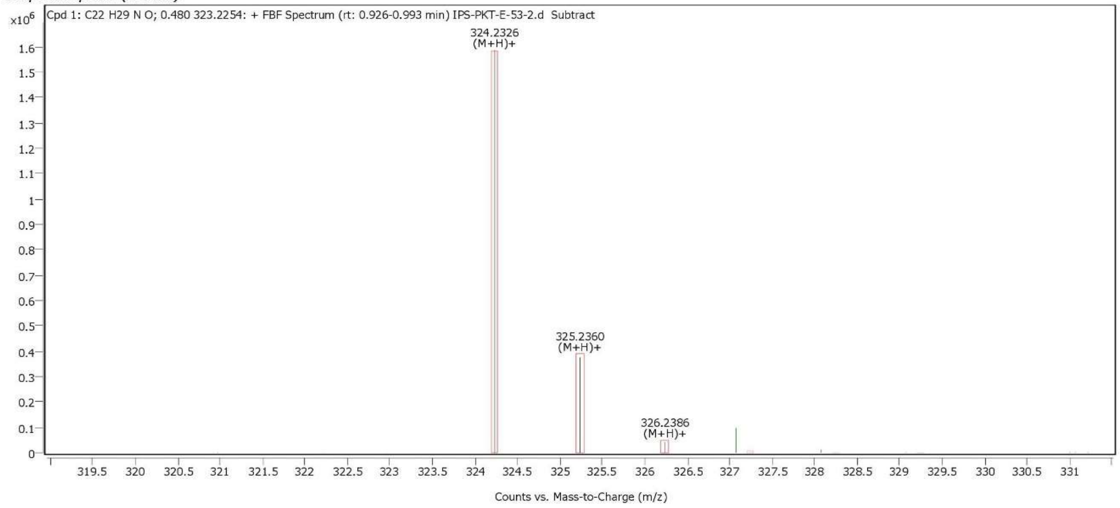

Compound ID Table

Cpd	Formula	Mass (Tgt)	Calc. Mass	Mass	Species	Diff(Tgt.ppm)	mDa
1	C22 H29 N O	323.2249	323.2254	324.2326	(M+H)+	1.39	0.45

ESI Mass spectrum of compound 2c

Compound 2d:

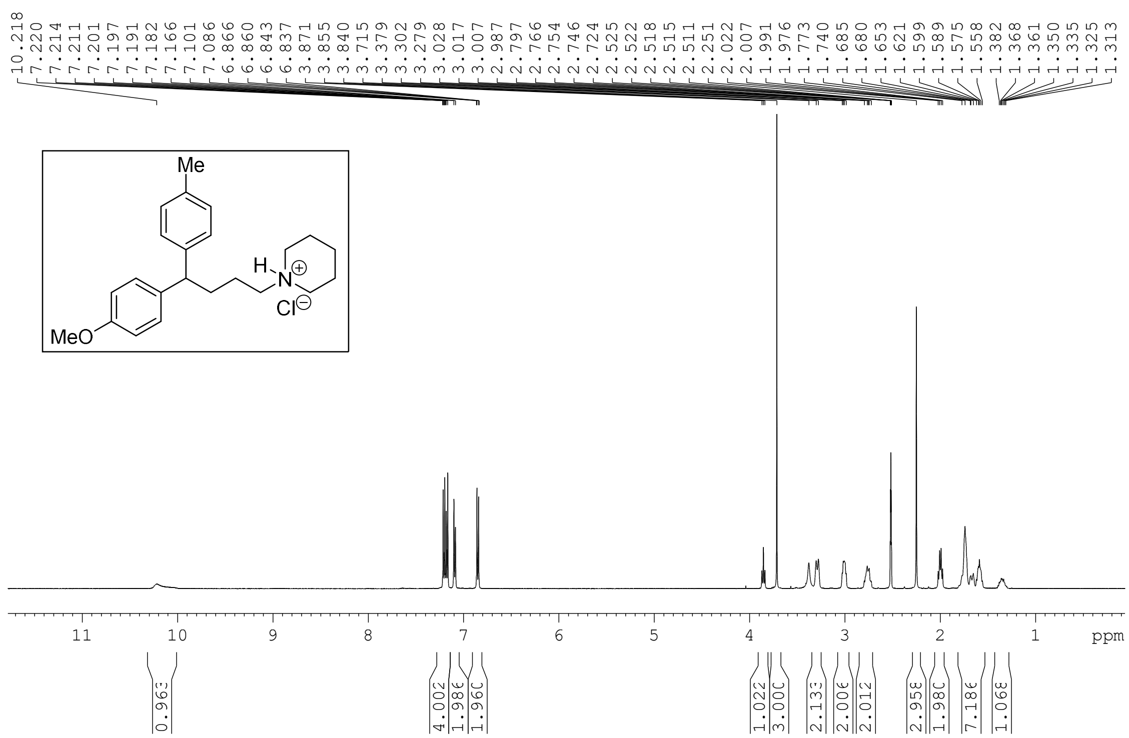

^{1}H NMR (DMSO-d_6, 500 MHz) spectrum of compound 2d

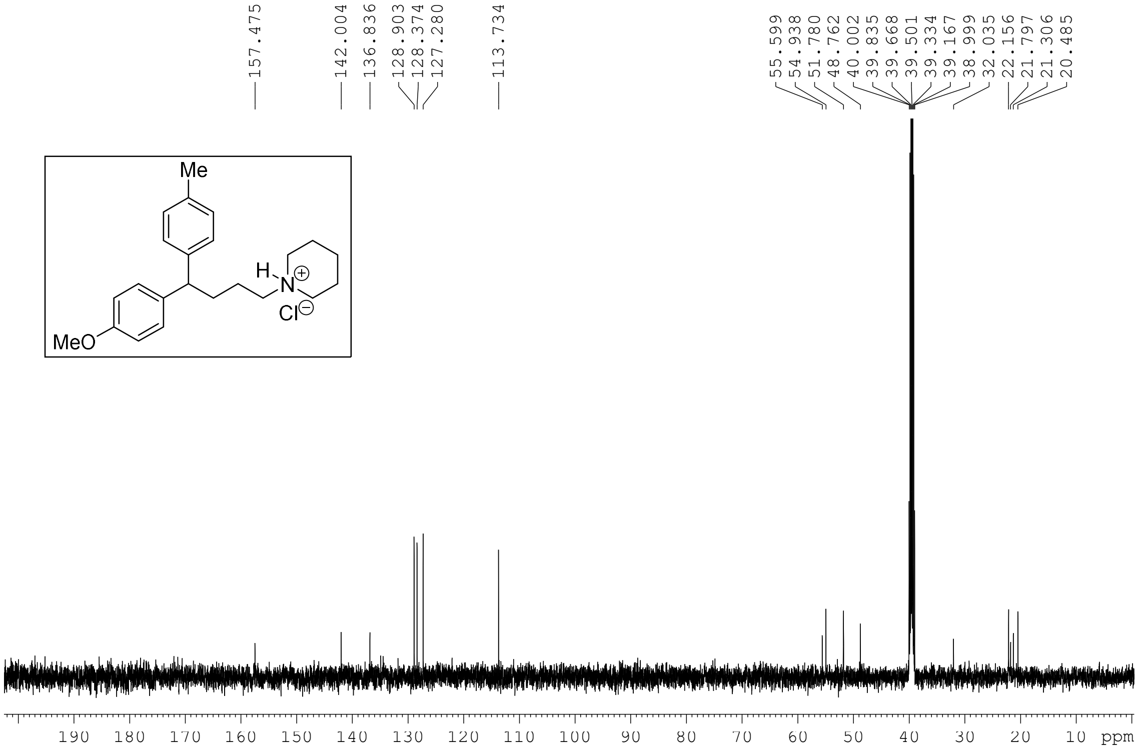

^{13}C NMR (DMSO-d_6, 125 MHz) spectrum of compound 2d

HRMS DST-FIST Funded, Department of Chemistry, IIT Madras

ESI Mass Report

**Name**	230719-29-IPS-PKT-E-63	**Data File Path**	D:\MassHunter\Data\2019\JULY-2019\IPS-PKT-E-63.d
**Sample ID**		**Acq. Time (Local)**	23-07-2019 19:21:11 (UTC+05:30)
**Instrument**	Instrument 1	**Method Path (Acq)**	D:\MassHunter\Methods\Direct Infusion_HPLC.m
**MS Type**	QTOF	**Version (Acq SW)**	6200 series TOF/6500 series Q-TOF B.08.00 (B8058.0)
**Inj. Vol. (ul)**	5	**IRM Status**	Success
**Position**	P1-C10	**Method Path (DA)**	D:\MassHunter\Methods\10.0\IIT-Target Screening_1.m
**Plate Pos.**		**Target Source Path**	
**Operator**		**Result Summary**	1 qualified (1 targets)

Compound Details

Cpd. 1: C23 H31 N O

Compound Spectra (overlaid)

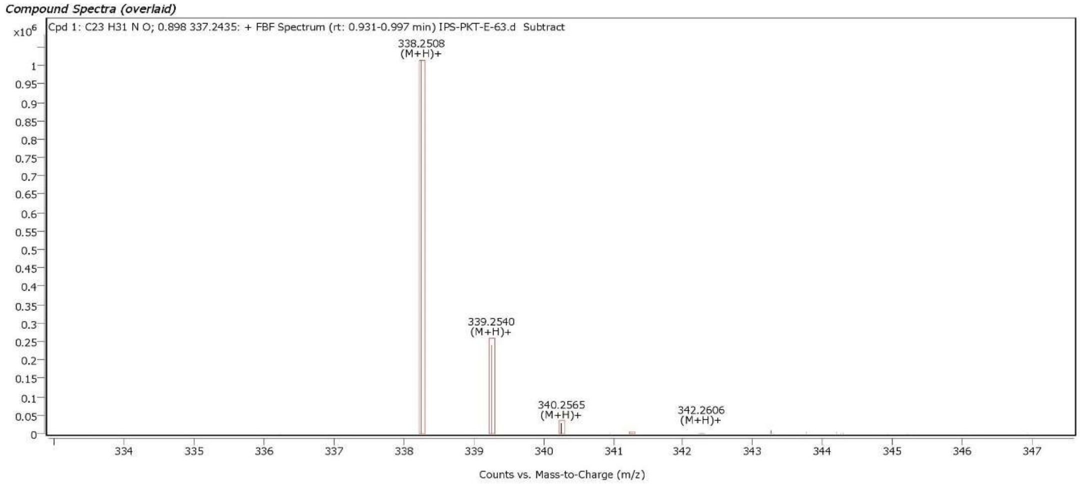

Compound ID Table

Cpd	Formula	Mass (Tgt)	Calc. Mass	Mass	Species	Diff(Tgt.ppm)	mDa
1	C23 H31 N O	337.2406	337.2435	338.2508	(M+H)+	8.73	2.94

ESI Mass spectrum of compound 2d

Compound 2e:

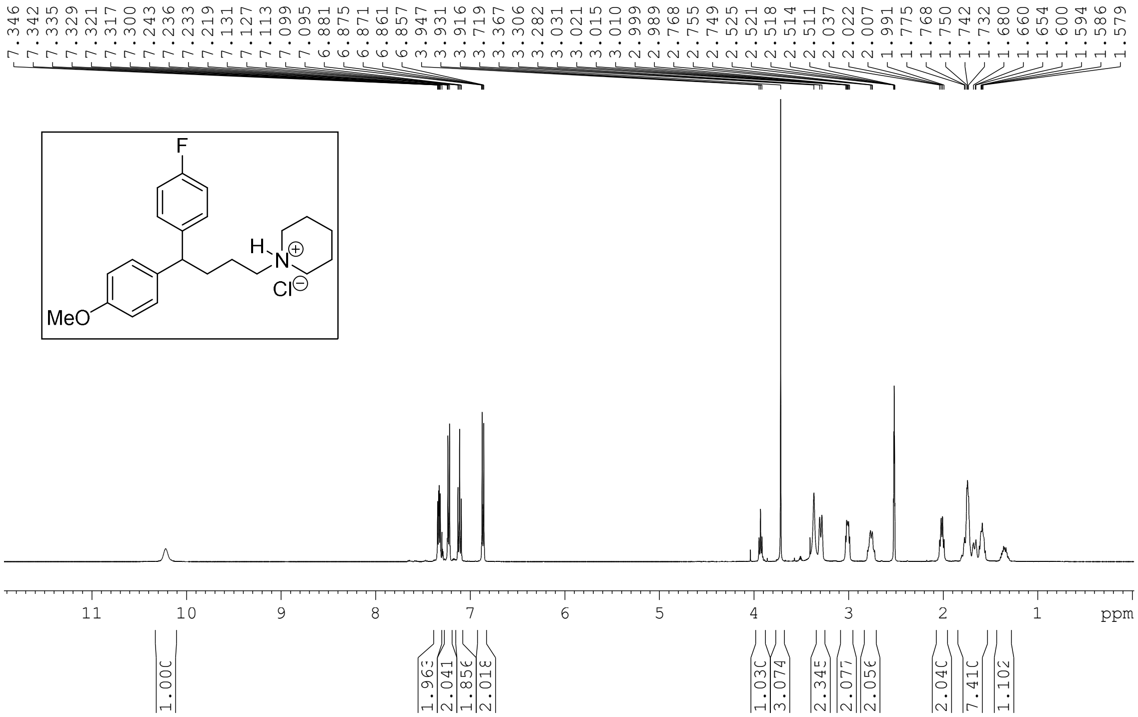

^{1}H NMR (DMSO-d_6, 500 MHz) spectrum of compound 2e

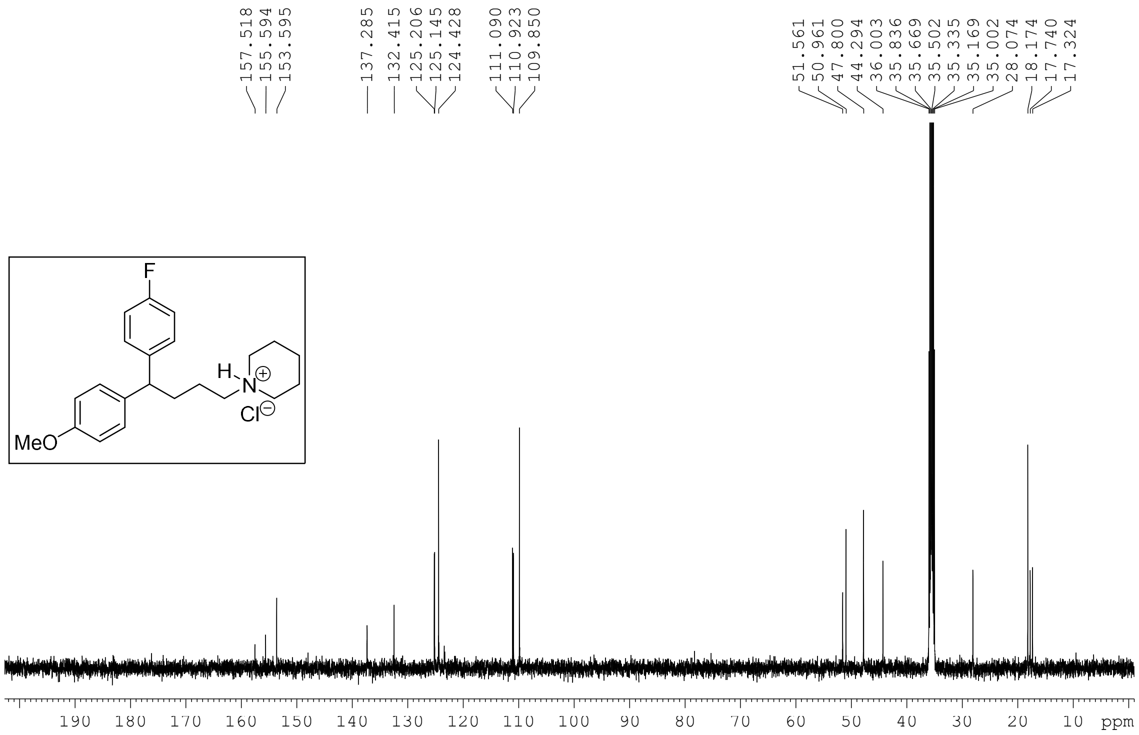

^{13}C NMR (DMSO-d_6, 125 MHz) spectrum of compound 2e

HRMS DST-FIST Funded, Department of Chemistry, IIT Madras

ESI Mass Report

**Name**	230719-29-IPS-PKT-E-69	**Data File Path**	D:\MassHunter\Data\2019\JULY-2019\IPS-PKT-E-69.d
**Sample ID**		**Acq. Time (Local)**	23-07-2019 18:06:29 (UTC+05:30)
**Instrument**	Instrument 1	**Method Path (Acq)**	D:\MassHunter\Methods\Direct Infusion_HPLC.m
**MS Type**	QTOF	**Version (Acq SW)**	6200 series TOF/6500 series Q-TOF B.08.00 (B8058.0)
**Inj. Vol. (ul)**	5	**IRM Status**	Success
**Position**	P1-C7	**Method Path (DA)**	D:\MassHunter\Methods\10.0\IIT-Target Screening_1.m
**Plate Pos.**		**Target Source Path**	
**Operator**		**Result Summary**	1 qualified (1 targets)

Compound Details

Cpd. 1: C22 H28 F N O

Compound Spectra (overlaid)

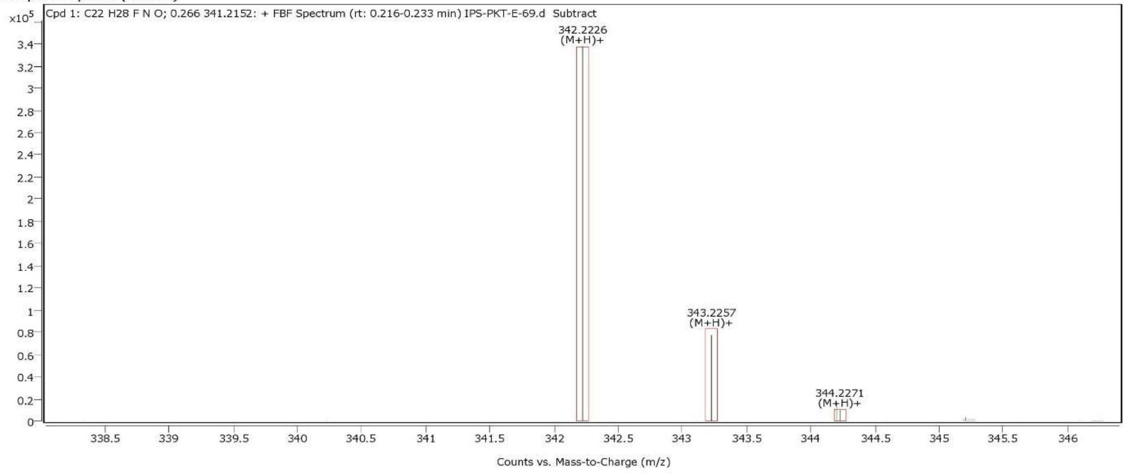

Compound ID Table

Cpd	Formula	Mass (Tgt)	Calc. Mass	Mass	Species	Diff(Tgt.ppm)	mDa
1	C22 H28 F N O	341.2155	341.2152	342.2226	(M+H)+	-0.81	-0.28

ESI Mass spectrum of compound 2e
